# Spatiotemporal cell junction assembly in human iPSC-CM models of arrhythmogenic cardiomyopathy

**DOI:** 10.1016/j.stemcr.2023.07.005

**Published:** 2023-08-17

**Authors:** Sean L. Kim, Michael A. Trembley, Keel Yong Lee, Suji Choi, Luke A. MacQueen, John F. Zimmerman, Lousanne H.C. de Wit, Kevin Shani, Douglas E. Henze, Daniel J. Drennan, Shaila A. Saifee, Li Jun Loh, Xujie Liu, Kevin Kit Parker, William T. Pu

**Affiliations:** 1 Disease Biophysics Group, John A. Paulson School of Engineering and Applied Sciences, Harvard University, Boston, MA 02134, USA; 2 Department of Cardiology, Boston Children’s Hospital, Boston, MA 02115, USA; 3 Department of Integrative Bioscience and Biotechnology, Sejong University, Seoul 05006, Republic of Korea; 4 Harvard Stem Cell Institute, Harvard University, Cambridge, MA 02138, USA; 5 Wyss Institute for Biologically Inspired Engineering, Harvard University, Boston, MA 02115, USA

**Keywords:** arrhythmogenic cardiomyopathy, human induced pluripotent stem cells, cardiac tissue engineering, disease modeling, cell pairs

## Abstract

Arrhythmogenic cardiomyopathy (ACM) is an inherited cardiac disorder that causes life-threatening arrhythmias and myocardial dysfunction. Pathogenic variants in Plakophilin-2 (*PKP2*), a desmosome component within specialized cardiac cell junctions, cause the majority of ACM cases. However, the molecular mechanisms by which *PKP2* variants induce disease phenotypes remain unclear. Here we built bioengineered platforms using genetically modified human induced pluripotent stem cell-derived cardiomyocytes to model the early spatiotemporal process of cardiomyocyte junction assembly *in vitro*. Heterozygosity for truncating variant *PKP2*^*R413X*^ reduced Wnt/β-catenin signaling, impaired myofibrillogenesis, delayed mechanical coupling, and reduced calcium wave velocity in engineered tissues. These abnormalities were ameliorated by SB216763, which activated Wnt/β-catenin signaling, improved cytoskeletal organization, restored cell junction integrity in cell pairs, and improved calcium wave velocity in engineered tissues. Together, these findings highlight the therapeutic potential of modulating Wnt/β-catenin signaling in a human model of ACM.

## Highlights

• Characterized the spatiotemporal assembly of cell junctions in hiPSC-CM cell pairs
• ACM pathogenic variant compromised cytoskeletal and cell junction self-assembly
• ACM pathogenic variant reduced calcium wave velocity in engineered cardiac tissues
• Wnt/β-catenin activation by SB216763 restored these *in vitro* ACM phenotypes

## Graphical abstract

**Figure:**
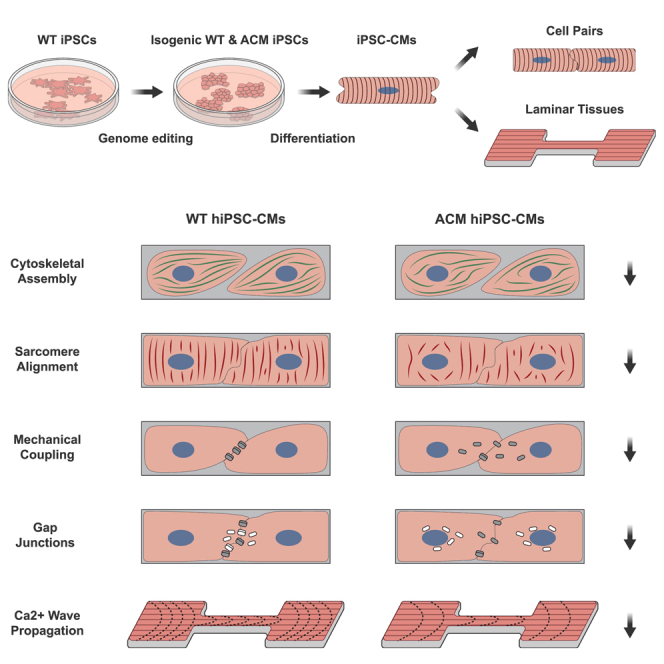

## Teaser

Pu et al. investigate the effect of a pathogenic arrhythmic cardiomyopathy variant on cell-cell junction structure and function. Using bioengineered tissues assembled from human induced pluripotent stem cell-derived cardiomyocytes, the team showed that the variant impaired cell-cell junction formation and slowed cardiac impulse propagation.

## Introduction

The cardiac intercalated disc is a specialized cell junction that mechanically and electrically couples cardiomyocytes. This coupling is enabled by the area composita, a hybrid adhesion and signaling complex, that includes adherens junctions, desmosomes, and gap junctions. Deleterious variants in desmosome genes cause arrhythmogenic cardiomyopathy (ACM), an inherited cardiomyopathy characterized by ventricular arrhythmias and progressive fibrofatty replacement of the myocardium, ultimately leading to heart failure (Austin et al., 2019). ACM affects an estimated 1 in 5,000 people, and more than one-half of ACM patients harbor genetic variants in plakophilin-2 (*PKP2*), a desmosome gene (Corrado et al., 2017; Gerull et al., 2004). Patient samples and animal models have shown that pathogenic *PKP2* variants induce misexpression and mislocalization of other area composita proteins, including plakoglobin and connexin-43 (CX43; official symbol *GJA1*), components of desmosome and gap junctions, respectively. This failure to properly assemble cell junctions is hypothesized to compromise the structural and electrical connectivity across cardiomyocytes and its functions in intracellular signaling, culminating in heart failure (Bhonsale et al., 2015; Cerrone et al., 2017; Cruz et al., 2015; Kirchner et al., 2012; Mazzanti et al., 2016; Oxford et al., 2007; Sato et al., 2009). Unfortunately, treatments for ACM are lacking, in part because of an incomplete understanding of the molecular events linking *PKP2* pathogenic variants to the dynamics of cell junction remodeling, arrhythmogenesis, and myocardial fibrofatty replacement.

At the molecular and cellular levels, mislocalization of plakoglobin is hypothesized to suppress Wnt/β-catenin signaling (Austin et al., 2019; Chelko et al., 2016; Garcia-Gras et al., 2006; Zhurinsky et al., 2000), an evolutionarily conserved pathway that regulates cardiomyocyte gene expression (Gessert and Kühl, 2010; Komiya and Habas, 2008). Previous studies have shown that SB216763, a small molecule that activates Wnt/β-catenin signaling by inhibiting glycogen synthase kinase 3 (GSK-3), restored cell junction integrity, and improved cardiac conduction and ejection fraction in mouse, rat, and zebrafish ACM models (Asimaki et al., 2014; Chelko et al., 2016; Padrón-Barthe et al., 2019). However, the cellular and junctional remodeling processes that occur with Wnt modulation have been difficult to track. Most ACM models including patient samples and animal models represent late-stage disease phenotypes resulting from extended periods of pathological remodeling with innate compensatory mechanisms. To facilitate dissection of the pathogenic role of ACM-causing variants and pathways within human cardiomyocytes, recent studies (Bliley et al., 2021; Inoue et al., 2022; Khudiakov et al., 2020; Kim et al., 2013; Zhang et al., 2021) have largely focused on using cardiomyocytes differentiated from human induced pluripotent stem cells (hiPSC-CMs).

hiPSC-CMs can recapitulate disease phenotypes (Thomas et al., 2022) and have been used to demonstrate aberrant metabolism, intracellular lipid accumulation, sarcomere assembly, calcium handling, contractility, and electrophysiology in ACM hiPSC-CMs (Austin et al., 2019; Bliley et al., 2021; Inoue et al., 2022; Khudiakov et al., 2020; Kim et al., 2013; Zhang et al., 2021). In this study, we developed a hiPSC-CM cell pair model to study the assembly of cell-cell junctions and their perturbation by a pathogenic *PKP2* nonsense variant, *PKP2 c*.*1237C>T*, p.R413X. We found that *PKP2*^*R413X/+*^ cells exhibited reduced Wnt/β-catenin signaling and altered cytoskeletal organization, which impaired the formation of cell-cell junctions. Stimulation of Wnt/β-catenin by SB216763 reversed the maladaptive structural and functional phenotypes, suggesting that Wnt/β-catenin signaling regulates cell junction dynamics via the cytoskeleton.

## Results

### Optimized cell pair platform to study hiPSC-CM cell-cell junction formation

The predominance of pathogenic sequence variants in desmosome genes implicates cell-cell junctions in ACM pathogenesis. The minimal cardiac functional unit that forms cell-cell junctions is a cell pair. To study the effect of ACM pathogenic variants on cell-cell junction formation, we optimized our previously described cell pair platform (Aratyn-Schaus et al., 2016; McCain et al., 2012a, 2012b). We micro-contact printed rectangular islands of extracellular matrix (ECM) proteins with a 14:1 length-to-width ratio, which models the dimensions of two human adult cardiomyocytes (average aspect ratio 7:1) connected end to end (Figure S1A). Guided by the area of unpatterned hiPSC-CMs (1,600 ± 101 μm^2^, mean ± SEM, Figures S1B and S1C), we used an ECM island area of 3,200 μm^2^ for cell pairs (Figure S1D). Initial experiments using substrates optimized for rat cardiomyocyte cell pairs (Aratyn-Schaus et al., 2016; McCain et al., 2012a, 2012b) resulted in low cell coverage. An ECM protein mixture of fibronectin and GelTrex (FN/GT) on soft polydimethylsiloxane (PDMS) substrates improved the consistency and adhesion of hiPSC-CMs by 73% compared with patterned substrates containing only fibronectin (Figures S1E–S1G). These optimizations achieved 64% cell coverage on the printed substrates (Figures S1F and S1G). Together, these data suggest that hiPSC-CMs require a more biomimetic ECM protein mixture to promote cell attachment and survival on engineered substrates *in vitro* in comparison with rat cardiomyocytes.

**Figure 1:**
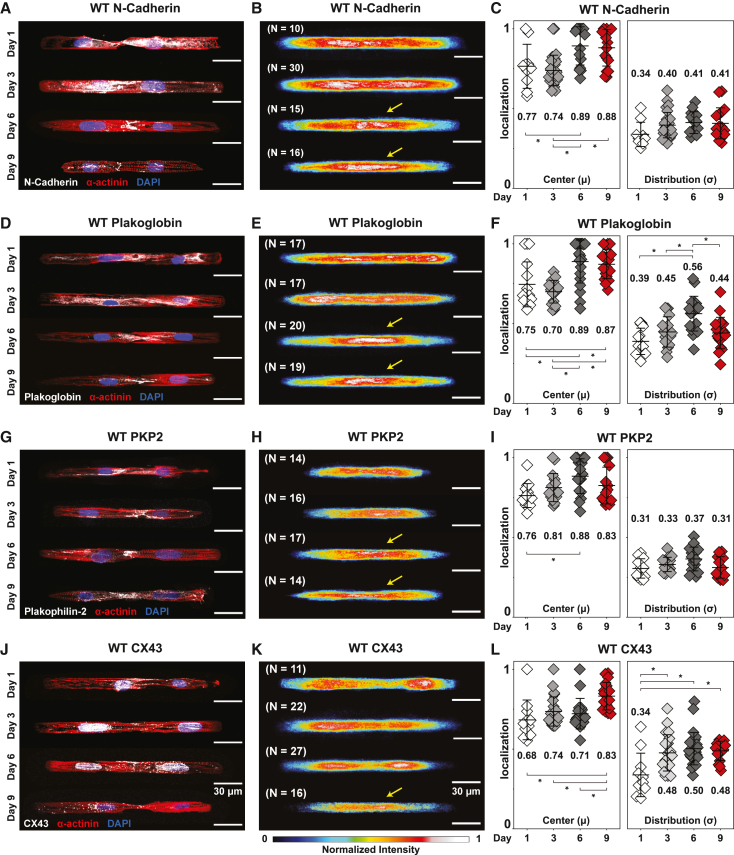
Spatiotemporal cell-cell junction assembly of WT hiPSC-CMs (A–L) See also Figures S1–S3. Immunofluorescence images, averaged heatmaps, and quantified protein localization of N-cadherin (A–C), plakoglobin (D–F), PKP2 (G–I), and CX43 (J–L). Representative images of WT hiPSC-CM cell pairs stained for nuclei (blue), α-actinin (red), and cell junction proteins (white). (B, E, H, K) Overlaid heatmaps represent the distribution of individual proteins averaged over all imaged cells for each time point. Yellow arrows indicate junctional localization. Quantified center and distribution of N-cadherin (C), plakoglobin (F), PKP2 (I), and CX43 (L). Mean ± SD. One-way ANOVA followed by Tukey’s honest significant difference (HSD) test: ^∗^p < 0.05. Cell pairs were aggregated from three to four independent differentiations.

Next, we examined the formation of cell junctions by hiPSC-CMs in our cell pair platform. hiPSC-CMs were allowed to mature in monolayer culture to post-differentiation day 30 and then replated on FN/GT patterned soft PDMS substrates. Formation of cell-cell junctions was then monitored over the subsequent 9 days (Figure S2A). We focused on N-cadherin, plakoglobin, PKP2, and CX43, components of the intercalated disc (Manring et al., 2018), and used immunostaining to monitor their distribution and localization during cell junction assembly. Consistently across cell pairs, N-cadherin, plakoglobin, and PKP2 staining localized at the cell-cell junction (Figures 1A–1I) by day 6, suggesting development of adhesive junctions comprising cadherins and desmosomes that mediate mechanical coupling. CX43 immunofluorescence localization at the cell-cell junction was observed at day 9 (Figures 1J–1L), suggesting that gap junction-mediated electrical coupling between cell pairs is established after mechanical coupling.

To summarize the immunostaining results across cell pairs and experimental batches, we took advantage of the reproducible shape and size of cell pairs to generate representative overlaid heatmaps of protein expression at each timepoint. For example, the superimposed immunofluorescence heatmaps showed that N-cadherin (Figure 1B), plakoglobin (Figure 1E), and PKP2 (Figure 1H) staining transition from a diffuse cytoplasmic distribution toward a distinct junctional localization over 6 days in culture. A few days later, CX43 staining consistently localized at cell-cell junctions (Figure 1K). Corresponding quantified values for the center (μ) and width/spread (σ) of each protein distribution (Figures S2B and S2C) further confirmed gradual localization of these junctional proteins to the cell-cell junction (Figures 1C, 1F, 1I, and 1L) over 9 days on the cell pair substrates *in vitro*.

The cytoskeletal network is another critical structure that forms via myofibrillogenesis during the early stages of cardiomyocyte development. Two cytoskeletal proteins, filamentous actin (F-actin) and sarcomere Z-disc protein α-actinin, align along the long and short axes, respectively, as cardiomyocytes develop and mature *in vitro*, and thus indicate the cell’s structural health or maturity. We quantified cell pair cytoskeletal and sarcomeric organization using the orientational order parameter (OOP) and Z-disc presence (Figures S2D–S2F), previously established metrics (Pasqualini et al., 2015; Sheehy et al., 2014; Wang et al., 2014). OOP describes the overall orientation of fibrillar structures based on their distribution, with 0 and 1 representing isotropic and anisotropic alignment, respectively, and Z-disc presence representing the overall fraction of Z-discs oriented in the cardiomyocyte short axis (Figure S2F). Since we standardized the cellular microenvironment, these metrics were similar across differentiation batches with variable plating efficiencies (Figures S2G and S2H).

Compared with unpatterned hiPSC-CMs on FN/GT-coated PDMS, patterned cell pairs exhibited greater F-actin and sarcomeric OOP (Figures S3A and S3B). Cytoskeletal organization was also well established by day 1, with only slight increases up to day 9. Sarcomeric α-actinin organization developed more slowly, reaching its maximal value at day 6. Since nuclear morphology reflects cytoskeletal tension (Bray et al., 2010; Lee et al., 2015), we quantified the nuclear aspect ratio to further assess cytoskeletal stresses. Compared with unpatterned hiPSC-CMs, cell pair hiPSC-CMs exhibited elongated nuclei (higher aspect ratio), with greater alignment between nuclear and cellular long axes (Figures S3C and S3D). Together, these results suggest that shape-controlled culture of hiPSC-CMs induced and standardized cytoskeletal and cell-cell junction formation.

### Impaired assembly of cell-cell junctions in *ACM* hiPSC-CM cell pairs

Truncating and missense variants in *PKP2* are the most frequent cause of ACM (Gerull et al., 2004). The consequences of *PKP2* pathogenic variants on spatiotemporal assembly of cell-cell junctions have yet to be shown in human cardiomyocytes or hiPSC-CMs. To address this question, we used the cell pair platform to compare cell-cell junction assembly in *PKP2* mutant and isogenic control hiPSC-CMs. Using CRISPR-Cas9-mediated genome editing (Wang et al., 2017), we created two iPSC lines (clones C93 and C98) heterozygous for *PKP2*^*R413X*^, an established pathogenic truncating variant (Figures S4A, S4B, and Table S1) (Syrris et al., 2006). Sanger sequencing did not show off-target mutagenesis at the three top predicted potentials, and digital karyotyping using Nanostring technology did not detect significant copy number variations compared with the parental line (Figures S4C and S4D). Genome-edited and control iPSCs expressed pluripotency markers (Figure S4E), and both genotypes exhibited comparable hiPSC-CM differentiation efficiencies (Figure S4F). Here these *PKP2*^*R413X/+*^ iPSCs are referred to as *ACM*; *ACM-C98* was used for the main experiments, and key results were validated using *ACM-C93* as indicated.

**Figure 2:**
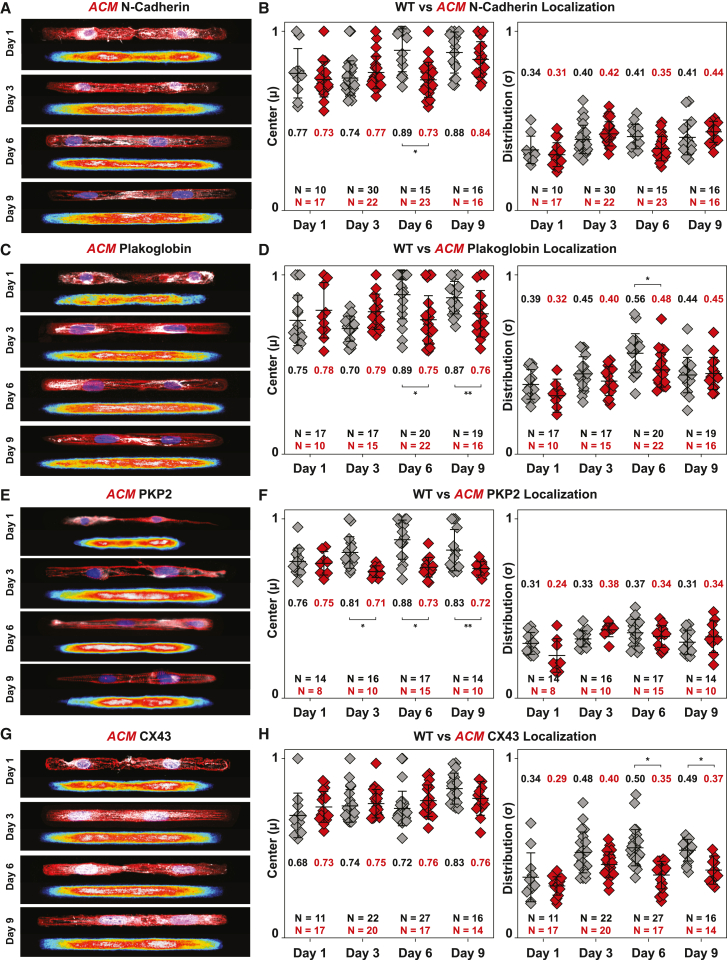
Compromised spatiotemporal assembly of cell-cell junctions in *ACM* hiPSC-CMs (A–H) See also Figure S4. Representative immunostained images and averaged heatmaps of *ACM* hiPSC-CM cell pairs stained for nuclei (blue), α-actinin (red), and cell junction proteins (white). Quantified centers and distributions of intercalated disc protein localization in isogenic WT control and *ACM* hiPSC-CM cell pairs for cell junction proteins are shown on the right. Mean ± SD. two-way ANOVA followed by Tukey’s multiple comparisons test: ^∗^p < 0.05, ^∗∗^p < 0.01. Cell pairs were aggregated from three to five independent differentiations.

*ACM* hiPSC-CMs cell pairs exhibited defective cell-cell junction assembly compared with isogenic controls. Isogenic control hiPSC-CM cell pairs consistently localized N-cadherin, plakoglobin, and PKP2 to cell-cell junctions by day 6, and CX43 by day 9 (Figure 1). In contrast, *ACM* cell pairs showed diffuse cytoplasmic distribution of N-cadherin until day 9 (Figures 2A and 2B), indicating delayed formation of adherens junctions. Furthermore, *ACM* cell pairs failed to properly localize plakoglobin, PKP2, and CX43 to cell-cell junctions during the 9-day culture period (Figures 2C–2H), indicating failure of desmosome and gap junction assembly. Plakoglobin stained diffusely throughout the cytoplasm, and PKP2 and CX43 showed aberrant peri-nuclear immunoreactivity. Quantitative analysis of protein localization over 9 days confirmed abnormal localization of plakoglobin (days 6 and 9), PKP2 (days 3, 6, and 9), and CX43 (days 6 and 9) (Figures 2D, 2F, and 2H).

**Figure 3:**
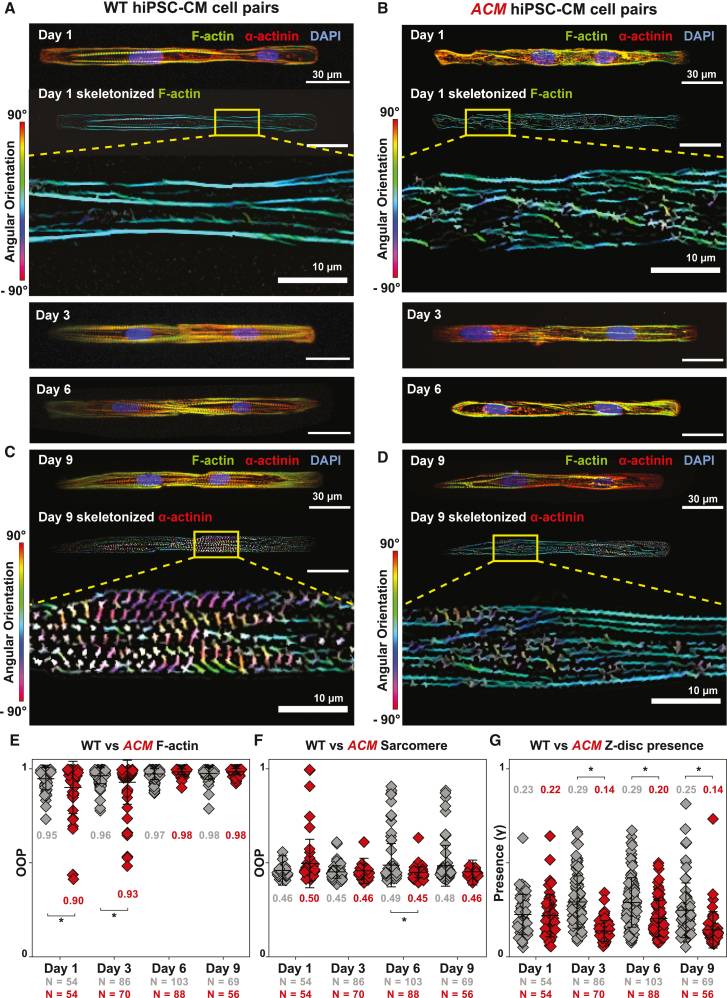
Compromised spatiotemporal assembly of the cytoskeletal filaments in *ACM* hiPSC-CMs (A and B) Representative images of isogenic WT (A) and ACM (B) hiPSC-CM cell pairs stained for F-actin (green), sarcomeric α-actinin (red), and DAPI (blue). Day 1 skeletonized F-actin images, pseudo-colored based on fibril angular orientation, are shown below with boxed region magnified in the inset. (C and D) Representative images of day 9 WT (C) and *ACM* (D) hiPSC-CM cell pairs with skeletonized α-actinin images below. Boxed regions are magnified in the inset. (E–G) Quantified values of cytoskeletal organization over 9 days in culture. ACM cell pairs showed slower F-actin alignment (E), comparable α-actinin alignment (F), and reduced Z-disc presence (G). Mean ± SD. Two-way ANOVA followed by Tukey’s multiple comparison test: ^∗^p < 0.05. Cell pairs were aggregated from four to five independent differentiations.

We then investigated the cytoskeletal organization in *ACM* cell pairs. Compared with isogenic controls, *ACM* cell pairs exhibited reduced F-actin organization and sarcomeric α-actinin formation (Figure 3). Quantitative analysis confirmed reduced F-actin alignment during the early cell pair assembly (days 1 and 3; Figure 3E) and subsequently reduced sarcomere organization (Z-disc presence at days 3, 6, and 9) (Figures 3F and 3G). Increased sarcomere organization in wild-type (WT) cell pairs on days 6 and 9 was associated with enhanced formation of cell-cell junctions, as indicated by junctional localization of intercalated disc proteins in WT compared with *ACM* cell pairs (Figure 2).

### SB216763 induces myofibrillogenesis and restores junctional integrity in *ACM* hiPSC-CM cell pairs

**Figure 4:**
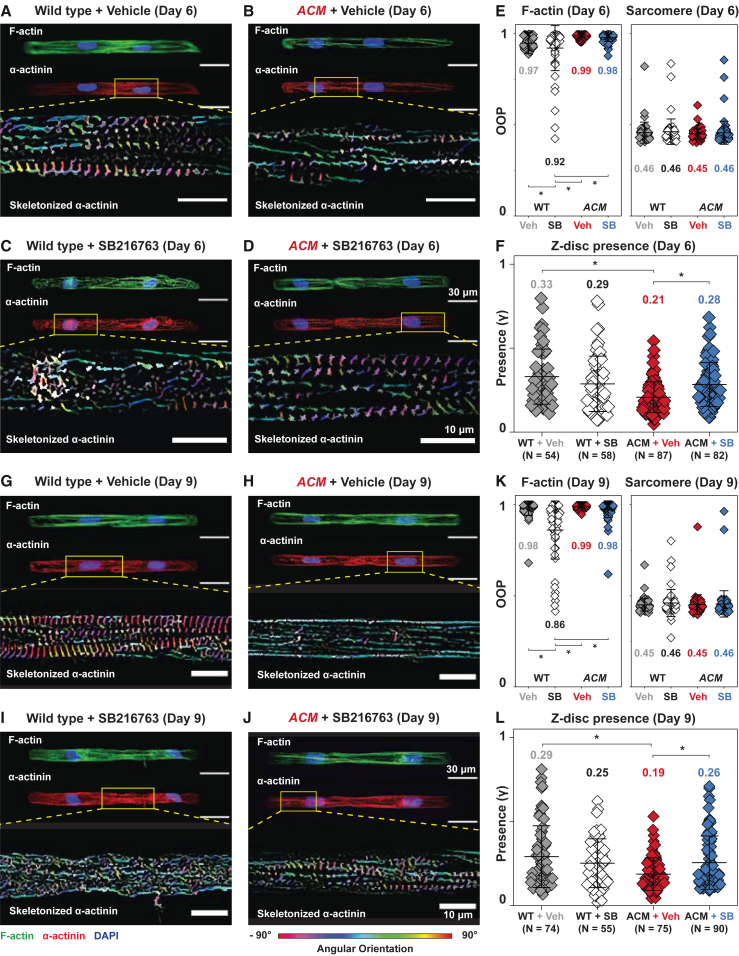
SB restores cytoskeletal integrity in day 6 and 9 *ACM* hiPSC-CMs (A–F) Day 6 WT and *ACM* hiPSC-CM cell pairs treated with DMSO (veh) or SB. Representative images of cell pairs stained for F-actin (green) and α-actinin (red), with corresponding skeletonized fibrils pseudo-colored based on angular orientation. (E) Quantified values for cytoskeletal organization (F-actin and sarcomere OOP) were unaffected, but modest improvement to sarcomere formation (z-disc presence) was observed in ACM + SB (F). (G–L) Day 9 WT and *ACM* hiPSC-CM cell pairs treated with veh or SB. Similar trends are observed with more pronounced significant improvements to sarcomere formation in ACM + SB were observed (L). Whereas SB improved cytoskeletal integrity in ACM cell pairs, it reduced these metrics (E, F, K, L) in WT cell pairs. Mean ± SD. Two-way ANOVA followed by Tukey’s multiple comparisons test: ^∗^p < 0.05. Cell pairs were aggregated from three to five independent differentiations.

**Figure 5:**
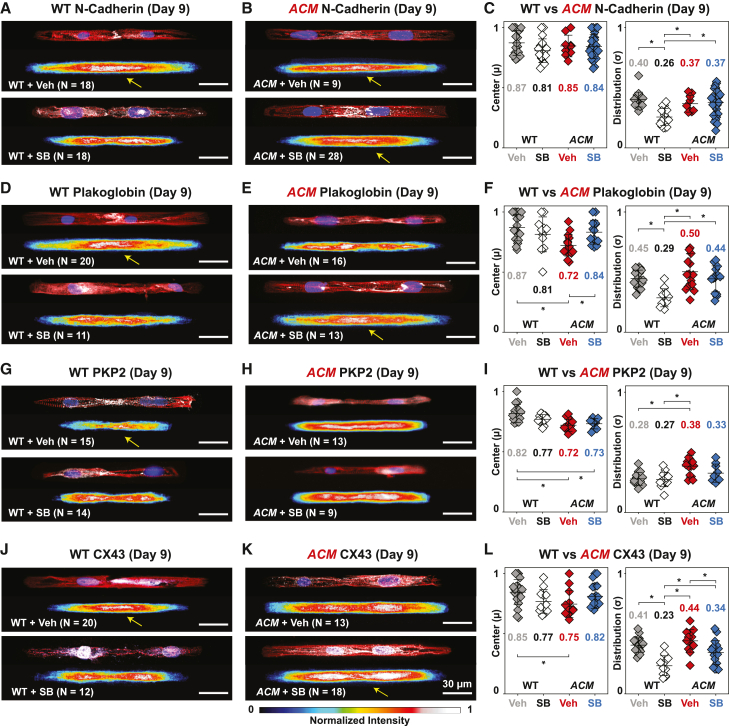
SB normalizes cell junction protein distribution in day 9 *ACM* hiPSC-CMs See also Figures S5 and S6. WT or ACM hiPSC-CM cell pairs treated with DMSO (veh) or SB and stained at day 9 for nuclei, α-actinin, and cell junction proteins. (A, B, D, E, G, H, J, and K). Representative images and corresponding averaged heatmaps. Yellow arrows indicate junctional localization. (C, F, I, and L) Quantitative analysis of cell junction protein localization center (left) and distribution (right) in WT or *ACM* hiPSC-CMs at day 9. Significantly impaired plakoglobin and CX43 localization in ACM + veh were restored by SB. N-cadherin and PKP2 were unaffected by SB in *ACM* hiPSC-CMs. Mean ± SD. Two-way ANOVA followed by Tukey’s multiple comparisons test: ^∗^p < 0.05.

Reduced Wnt/β-catenin signaling is hypothesized to contribute to ACM pathogenesis (Austin et al., 2019; Garcia-Gras et al., 2006). SB216763, a small molecule GSK-3 inhibitor and Wnt activator, restores junctional protein localization and normalizes electrical activity in animal and human cell models of ACM (Asimaki et al., 2014; Chelko et al., 2016). Here we asked if modulating the Wnt/β-catenin pathway via SB216763 would restore cytoskeletal organization and cell junction integrity in the hiPSC-CM cell pair model of ACM (Figures 4 and 5).

The effect of SB216763 on *ACM* and control cell pairs is shown in representative images and corresponding quantitative analysis at day 6 (Figures 4A–4F) and day 9 (Figures 4G–4L). At both time points, ACM + vehicle (veh) had fewer vertically oriented α-actinin fibrils (Z-disc presence) than WT + veh, and this deficiency was rescued by SB216763 (Figure 4). While SB216763 enhanced cytoskeletal organization of *ACM* cell pairs, it had the opposite effect in WT cell pairs: WT + SB216763 (SB) cell pairs had significantly lower actin alignment (F-actin OOP) compared with WT + veh cell pairs (Figures 4E and 4K).

Next, we examined the effects of SB on cell-cell junction formation. N-cadherin localization to cell-cell junctions in *ACM* cell pairs was not significantly affected by SB. Both ACM + veh and ACM + SB exhibited delayed N-cadherin junctional localization with cytoplasmic distribution on day 6 (Figures S5A and S5B) and junctional localization by day 9 (Figures 5A–5C). In contrast, SB treatment made plakoglobin in *ACM* comparable with WT by day 9 (Figures 5D and 5E). However, plakoglobin localization remained abnormal on day 6 (Figures S5C and S5D), suggesting partial rescue with continued abnormalities in desmosome assembly kinetics. PKP2 localization was also not affected by SB. Both ACM + veh and ACM + SB exhibited mislocalization of PKP2 on days 6 and 9 (Figures S5E, S5F, and 5G–5I). On day 9, CX43 exhibited junctional localization in WT + veh, whereas it was primarily perinuclear in ACM + veh cell pairs. After SB treatment, CX43 localization in ACM + SB became comparable with WT + veh. (Figures 5J–5L). We further validated the effects of SB on *PKP2* mutant cell-cell junctions by repeating the experiments on a second independent *ACM* clone, *ACM-C93*. Baseline defects and responses to SB were comparable between clones (Figure S6). Together these data indicate that SB ameliorates junctional complex assembly defects observed in *ACM* hiPSC-CM cell pairs.

Whereas SB ameliorated abnormal junctional protein localization in *ACM* cell pairs, it disrupted junctional localization in WT hiPSC-CM cell pairs. WT + SB showed reduced junctional localization of N-cadherin first on day 6 (Figures S5A and S5B). By day 9, N-cadherin, plakoglobin, and CX43 became mislocalized to the peri-nuclear region, indicated by lower values for centers and reduced distribution widths (Figures 5C, 5F, and 5L). Together with its effect on F-actin alignment (Figure 4), these data indicate that SB impaired WT cell pair cytoskeletal and junctional assembly.

**Figure 6:**
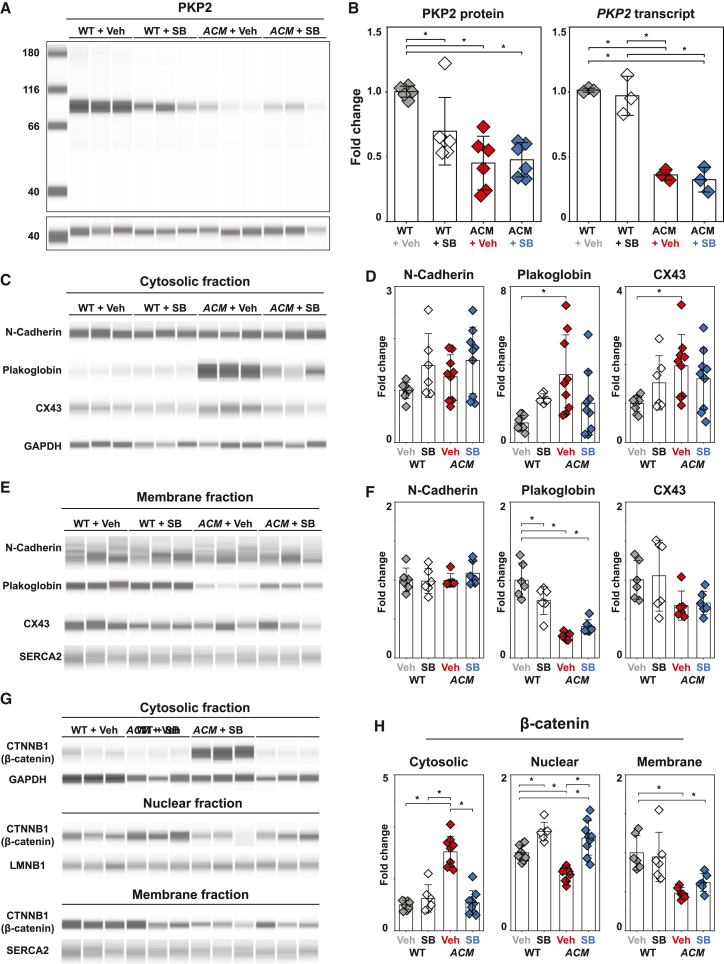
Abundance of proteins in cytoplasmic, nuclear, and membrane fractions of day 9 hiPSC-CMs (A and B) See also Figure S7. Capillary western analysis of PKP2 and GAPDH. Relative changes to PKP2 protein and transcript levels with SB treatment in WT and ACM hiPSC-CMs. Transcript levels were measured by qRT-PCR and normalized to *GAPDH*. (C–F) Capillary western analysis of cell junction proteins in cytosolic or membrane fractions. Relative levels of cell junction proteins were normalized to GAPDH (cytosolic fraction) or SERCA2 (membrane fraction) and expressed relative to WT + veh. (G and H) β-Catenin in cytosolic, nuclear, and membrane fractions was measured by capillary western. Relative β-catenin was normalized LMNB1 (nuclear fraction), GAPDH (cytosolic fraction), or SERCA2 (membrane fraction) and expressed relative to WT + veh. Mean ± SD. Two-way ANOVA followed by Tukey’s multiple comparisons test: ^∗^p < 0.05. Data represent three technical replicates from two to three independent differentiations.

To gain further insight into the molecular mechanisms underlying the observed structural changes, we used subcellular fractionation and capillary western blotting to quantify changes in protein levels or localization. Because of the amount of protein needed for these studies, monolayer cultures were used. The PKP2 protein level was significantly reduced in ACM hiPSC-CMs (Figure 6A), indicating that the R413X allele impairs protein expression. If translated, *PKP2*^*R413X*^ would produce a 45-kDa peptide. We did not observe a truncated PKP2 protein (Figures 6A and 6B), suggesting that the PKP2^R413X^ protein is not synthesized or is unstable. Quantification of *PKP2* mRNA demonstrated reduced transcript levels in *PKP2*^*R413X/+*^ cells, suggesting the mutant allele undergoes nonsense-mediated RNA decay. Together, these data suggest that phenotypic defects in *PKP2*^*R413X/+*^ hiPSC-CMs are caused by a haploinsufficiency of PKP2. SB did not alter PKP2 transcript or protein levels, indicating that it affects downstream processes rather than expression of the mutant allele.

Next, we examined the level of junctional proteins (Figures 6C–6F). N-cadherin expression in either cytosolic or membrane fractions was not significantly altered by *PKP2* genotype or SB treatment (Figures 6D and 6F left). In veh-treated cells, plakoglobin was significantly decreased in the membrane fraction and increased in the cytosolic fraction of ACM compared with WT (Figures 6D and 6F middle), consistent with impaired desmosome assembly and plakoglobin membrane localization. SB did not increase plakoglobin in the membrane fraction in ACM monolayers. This result contrasts with improved junctional plakoglobin localization observed in ACM cell pairs by immunostaining (Figure 5F), perhaps reflecting differences in cell junction assembly in cell pairs and monolayers. CX43 was significantly elevated in the cytosolic fraction of ACM cells (Figure 6D right), suggesting impaired trafficking or membrane localization. Correspondingly, CX43 levels were lower in the ACM membrane fraction (Figure 6F right), although this difference was not statistically significant because of within-group variation. While SB improved CX43 localization at the junction of ACM cell pairs, we did not detect a significant effect on membrane or cytosolic fractions of monolayers, again suggesting differences in cell junction assembly between cell pairs and monolayers.

SB enhances canonical Wnt signaling by stabilizing cytoplasmic or nuclear β-catenin, the effector of Wnt signaling (Zhurinsky et al., 2000). Nuclear β-catenin was significantly decreased in *ACM* hiPSC-CMs compared with isogenic controls, consistent with reduced canonical Wnt signaling (Figures 6G and 6H). Cytoplasmic β-catenin was elevated in *ACM* hiPSC-CMs compared with WT, suggesting impaired nuclear localization. SB treatment of *ACM* hiPSC-CMs significantly reduced cytosolic and significantly increased nuclear β-catenin, confirming activation and normalization of Wnt/β-catenin signaling. Increased nuclear β-catenin was also observed in WT hiPSC-CMs. Consistent with prior studies (Asimaki et al., 2014; Chelko et al., 2016), these data indicate that SB increases canonical Wnt signaling in ACM hiPSC-CMs, which likely contributes to improved cell-cell junction assembly in ACM cell pairs. We also note increased canonical Wnt signaling in WT hiPSC-CMs, which may impair their maturation and cell-cell junction assembly.

### SB rescued calcium wave velocity in *ACM* hiPSC-CM tissues

**Figure 7:**
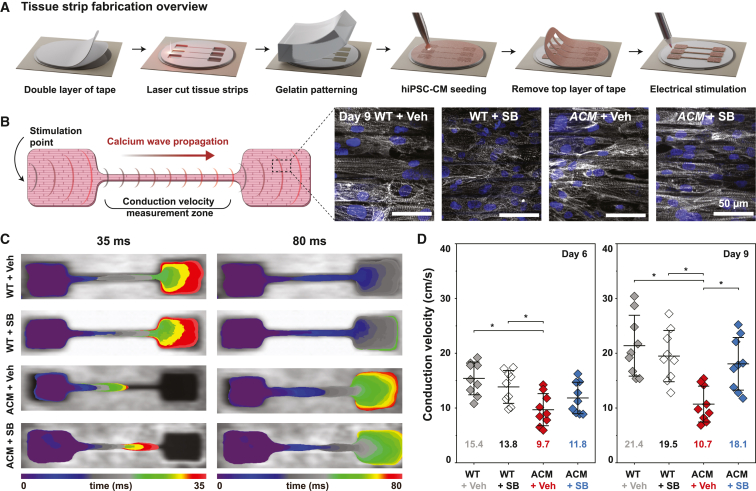
Ca^2+^ wave propagation velocity is reduced in *ACM* tissues and restored by SB (A) Schematic illustration of engineered tissue fabrication. (B) Design of tissue constructs for Ca^2+^ optical mapping experiments. Representative images of anisotropic tissues stained for sarcomeric α-actinin and nuclei demonstrate iPSC-CM alignment by micro-molded gelatin substrate. (C) Calcium wavefront isochrone maps for day 9 WT + veh, WT + SB, ACM + veh, and ACM + SB tissues at two representative time points (35 and 80 ms). (D) Ca^2+^ wave propagation velocities of WT and *ACM* tissues at day 6 and day 9, with and without SB treatment. Mean ± SD. Two-way ANOVA followed by Tukey’s multiple comparisons test: ^∗^p < 0.05.

A hallmark of ACM, particularly in its early concealed phase, is arrhythmia out of proportion to clinically apparent structural remodeling (Austin et al., 2019). To model arrhythmogenesis in ACM, we engineered tissues using isogenic WT and *ACM* hiPSC-CMs to quantify changes to cardiac propagation *in vitro*. We engineered biomimetic tissues with micro-molded ridges to promote hiPSC-CM alignment (Lee et al., 2022). An elongated, narrow neck in the tissues (Figures 7A and 7B) was designed to establish a favorable electrical source-sink relationship and enhance Ca^2+^ wave propagation into the neck. *ACM* or isogenic WT control hiPSC-CMs seeded on these substrates were grown to confluence for 6 to 9 days to allow for cell junction formation (Figure S7). We electrically paced the tissues by point stimulation at the larger base and recorded Ca^2+^ wave propagations through the neck using a Ca^2+^-sensitive fluorescent dye, X-Rhod-1. Ca^2+^ wave velocities of WT hiPSC-CM tissues averaged 15.4 ± 3.0 cm/s at day 6 and 21.4 ± 5.6 cm/s at day 9 (Figures 7C and 7D; Movie S1). These velocities are substantially higher than similar monolayer tissues without geometric constraints (Park et al., 2019) and comparable with velocities recorded from three-dimensional engineered heart tissue (Chang et al., 2022; Zhang and Pu, 2018). In comparison, *ACM* tissues exhibited significantly slower average Ca^2+^ wave propagation velocities at both day 6 (7 ± 2.9 cm/s) and day 9 (10.7 ± 3.3 cm/s) (Figure 7D; Movie S1).

We investigated the effect of SB on Ca^2+^ wave propagation in control and *ACM* tissues. SB did not significantly affect the propagation velocity of control tissues at either day 6 or 9 (Figure 7D). In contrast, SB dramatically increased Ca^2+^ wave velocities of *ACM* tissues at day 9 to 18.1 ± 4.8 cm/s, a velocity comparable with controls (Figure 7D). This increase in propagation velocity of *ACM* tissues was not observed on day 6, consistent with our observations in cell pairs that SB significantly changed cell junction localization on day 9, but not day 6.

We analyzed the effects of SB on cell junction protein localization in engineered tissues. Immunostaining with cell junction markers demonstrated that SB improved plakoglobin and CX43 localization in *ACM* tissues from a diffuse cytoplasmic distribution toward a more distinct localization around cell borders (Figures S7D and S7F), suggesting that it has similar effects on cell junction protein localization in *ACM* tissues and cell pairs. Together, these results indicate that SB remodeled cell-cell junctions of *ACM* hiPSC-CMs and markedly improved Ca^2+^ propagation velocity of *ACM* tissues.

## Discussion

In this study, we evaluated the effects of an established *ACM* pathogenic variant (*PKP2*^*R413X/+*^) on cell-cell junctions in engineered cardiac tissues. *PKP2*^*R413X/+*^ cells expressed decreased PKP2 protein, which impaired sarcomere and cytoskeletal assembly, consistent with prior studies (Inoue et al., 2022; Zhang et al., 2021). Our findings further suggest that *PKP2*^*R413X/+*^ impairs formation of cell-cell junctions in human iPSC-CM models and that these changes are mitigated by SB’s activation of Wnt/β-catenin signaling.

We leveraged the accessibility of hiPSC-CMs to monitor the spatiotemporal assembly of human cardiomyocyte junctions and their derangement by a pathogenic ACM variant. Cells organize their cytoskeletal architecture in response to geometric cues in the ECM (Grosberg et al., 2011). As the cellular architecture develops and cell-cell interactions dominate cell-ECM interactions (McCain et al., 2012a), the directionality of stresses within the cell changes, affecting nuclear morphologies (Bray et al., 2010; Lee et al., 2015). Cytoskeletal alignment also mediates the formation and localization of adherens junctions between cells, thus regulating desmosome and gap junction localization (Peters et al., 1994; Saffitz and Kléber, 2004; Vite and Radice, 2014). In healthy hiPSC-CM cell pairs, ECM-induced actin alignment occurred within 24 h, nuclear elongation, and adherens junction (N-cadherin) localization at cell junctions by day 6, and gap junction (CX43) localization by day 9. These processes were all disrupted in *PKP2*^*R413X/+*^ hiPSC-CMs, starting with delayed actin alignment, then reduced sarcomere and cell junction formation, and ultimately mislocalization of plakoglobin and CX43. The alterations to junctions observed at day 9 in ACM cell pairs, characterized by reduced plakoglobin and CX43 along with preserved N-cadherin, mirror steady-state observations in human ACM hearts (Asimaki et al., 2009; Austin et al., 2019).

ACM sequence variants have been associated with reduced Wnt/β-catenin signaling (Garcia-Gras et al., 2006; Zhurinsky et al., 2000). Therapeutic intervention through increased Wnt/β-catenin signaling, via inhibition of GSK-3, ameliorated ACM phenotypes in zebrafish, rat cultured cardiomyocyte, and mouse models (Asimaki et al., 2014; Chelko et al., 2016). Likewise, we observed reduced Wnt/β-catenin signaling in *PKP2*^*R413X/+*^ mutant human iPSC-CMs, and this was ameliorated with SB treatment. Specifically, SB induced myofibrillogenesis, restored mechanical and electrical coupling in *PKP2* mutant cell pairs, and improved Ca^2+^ wave velocity in *PKP2* mutant tissues. Our findings suggest that aberrant myofibrillogenesis and reduced Wnt/β-catenin signaling contribute to maladaptive cell junction formation and abnormalities in excitation-contraction coupling.

Arrhythmias in ACM are likely influenced by multiple factors, including cell death, fibrofatty myocardial replacement (Austin et al., 2019), aberrant intracellular Ca^2+^ homeostasis (Cerrone et al., 2017), and altered cardiac sodium currents (Khudiakov et al., 2020; Sato et al., 2009). Our data demonstrate that ACM strongly reduces Ca^2+^ wave propagation, which would increase myocardial vulnerability to re-entrant arrhythmias. Reduced Ca^2+^ wave propagation was likely caused by impaired formation of cell-cell junctions and mis-localization of CX43. Previous studies have directly correlated CX43 immunofluorescence to cell-to-cell conductance across cardiomyocytes (McCain et al., 2012b). Treating *PKP2*^*R413X/+*^ hiPSC-CMs with SB improved CX43 localization and restored Ca^2+^ wave velocities in *PKP2*^*R413X/+*^ tissues, changes that would be anticipated to reduce the myocardial substrate’s vulnerability to arrhythmia.

In contrast, we observed that GSK-3 inhibition and hyperactivation of Wnt/β-catenin via SB impaired the structural maturation of healthy hiPSC-CM cell pairs. Hyperactivation of Wnt/β-catenin initially disrupted cytoskeletal organization and mechanical coupling, followed by mislocalization of gap junctions in WT hiPSC-CMs. Regulation of Wnt/β-catenin signaling is critical for the differentiation of cardiac myocytes, and activation of this pathway in differentiated WT hiPSC-CMs promoted de-differentiation and proliferation (Buikema et al., 2020). However, in engineered tissues, we did not observe a deleterious effect of SB on cytoskeletal organization and cell coupling. This might suggest that cues in assembled tissues stabilize cell junctions and antagonize the de-differentiation effect observed in isolated cells (Buikema et al., 2020) or cell pairs. Consistent with this interpretation, SB did not adversely affect WT zebrafish or mice (Asimaki et al., 2014; Chelko et al., 2016). However, the deleterious effects that we observed on WT hiPSC-CMs raise concerns about cardiac effects of long-term exposure to SB. These add to existing concerns about oncogenic risks (Chelko et al., 2019; Zhan et al., 2017) and indicate that further studies of this class of compounds are required to understand their therapeutic mechanisms and potential adverse consequences in mature, *de facto* human cardiomyocytes. It is likely that other consequences of GSK-3 inhibition by SB also contribute. GSK-3 plays an integral role in cytoskeletal remodeling, and its perturbation modulated focal adhesion dynamics (Kobayashi et al., 2006), and actin and microtubule organization (Hajka et al., 2021).

In summary, our results demonstrate that *PKP2*^*R413X/+*^ reduces Wnt/β-catenin signaling and perturbs cytoskeletal organization in human iPSC-CMs, leading to abnormal cell-cell junctions and impaired Ca^2+^ wave propagation. Furthermore, SB activated Wnt/β-catenin signaling, normalized cell-cell junctions, and rescued Ca^2+^ wave propagation in human *PKP2*^*R413X/+*^ hiPSC-CM tissues, independent of changes to PKP2. These findings demonstrate our capacity to correlate structure-function relationships across spatial scales from genetic disease-causing variant to cell pairs and tissues, and to leverage the accessibility of hiPSC-CMs to analyze cardiomyocyte junction assembly *in vitro*. Future studies are required to determine the extent to which our findings for *PKP2*^*R413X/+*^ extend to other variants in *PKP2* and other ACM genes.

## Experimental procedures

Detailed experimental procedures are provided in the supplemental experimental procedures.

### Resource availability

Requests for resources and reagents will be fulfilled by the corresponding authors.

#### Corresponding author

Further information and requests for resources, reagents, and materials should be directed to and will be fulfilled by corresponding authors Kevin Kit Parker (kkparker@g.harvard.edu) and William T. Pu (william.pu@cardio.chboston.edu)

### hiPSC-CM differentiation, culture, and micropatterning

The reference cell line WTC-Cas9 was derived from WTC-11 hiPSCs by inserting a doxycycline-inducible SpCas9 transgene (WTC-Cas9). The pathogenic *PKP2*^*R413X/+*^ (*PKP2 c*.*1237C>T*) variant was introduced by CRISPR-Cas9 genome editing (Wang et al., 2017). Oligo sequences used are provided in Table S1. Cardiomyocytes were differentiated from iPSCs using WNT modulation (Lian et al., 2013) (Figure S2A).

PDMS stamps, created by photolithography (array of 14:1 rectangles 211 μm × 15 μm) were used to microcontact print ECM (fibronectin, Geltrex, or 1:1 fibronectin:Geltrex) onto PDMS-coated glass coverslips Figure S1). PDMS stamps (25-mm wide ridges, 4-mm grooves, and 5-mm groove depth) were used to micromold gelatin on a glass coverslip within a laser-cut acrylic mold, by modification of previously published protocols (Lee et al., 2022).

### Ca^2+^ optical mapping and propagation velocity calculation

Samples were loaded with 2 μM X-Rhod-1 AM (Invitrogen, X14210). Relative cytoplasmic Ca^2+^ was imaged using a modified tandem-lens macroscope as described previously (Park et al., 2019) under 1 Hz electrical point stimulation. Data were analyzed using MATLAB (MathWorks) and the MiCAM imaging software (Scimedia) (Lee et al., 2022).

### Immunostaining and data analysis

Samples were immunostained and imaged on a spinning disk confocal microscope (Olympus IX83, Andor spinning disk). Images were analyzed to quantify OOP and cytoskeletal alignment using previously published methods (Pasqualini et al., 2015; Sheehy et al., 2014; Wang et al., 2014), with slight modifications. Image preprocessing was performed with ImageJ/FIJI using the tubeness and OrientationJ plugins. Preprocessed images were then analyzed using MATLAB scripts (Pasqualini et al., 2015).

Protein localization analysis and heatmaps were performed using automated ImageJ/FIJI and MATLAB image processing scripts (Kim, 2022). Acquired cell pairs expressed sarcomeric α-actinin, a cardiomyocyte marker, covered most of the patterned area, and contained two distinct nuclei. Cell junction markers were not used to decide to include or exclude a cell pair. Cells with nuclei <50 μm apart and horizontal cytoskeletal filaments spanning across both nuclei were excluded as likely binucleated cells.

### Statistical analysis

Bar graphs indicate mean ± SD. Boxplots represent the interquartile range and median (box and center line) and 1.5 times the interquartile range (whiskers). Statistical analysis across calculated values were conducted using one-way ANOVA or two-way ANOVA followed by Tukey’s honest significant difference test or test for multiple comparisons using OriginPro (ver 2023, OriginLab Corporation). Statistically significant p values (p < 0.05 and p < 0.1) are indicated within the graphs where appropriate.

## Author contributions

S.L.K., M.A.T., K.Y.L., L.A.M., K.K.P., and W.T.P. designed research; S.L.K., M.A.T., and K.Y.L. performed research; S.C., J.F.Z., L.D.W., K.S., D.E.H., L.J.L., and X.L. contributed new reagents/analytic tools; S.L.K., M.A.T., and K.Y.L., analyzed data; and S.L.K., M.A.T., K.K.P., and W.T.P. wrote the paper.

## Data Availability

Analysis code generated and used in this study are available at https://doi.org/10.5281/ZENODO.7120682.

## Supplemental information

Document S1. Figures S1–S7 and Table S1 and Supplemental experimental procedures

Table S2. PKP2 protein and transcript raw data

Table S3. Fractionated western blot raw data

Supplementary movie S1. Ca^2+^ wave propagations in engineered tissues

Document S2. Article plus supplemental information
