# Supplementary material for: Spatiotemporal cell junction assembly in human iPSC-CM models of arrhythmogenic cardiomyopathy
Source: Stem Cell Reports. 2023 Aug 17;18(9):1811–26. doi: 10.1016/j.stemcr.2023.07.005 (PMC10545490; doi:10.1016/j.stemcr.2023.07.005)
Supplement: Document S1. Figures S1–S7 and Table S1 and Supplemental experimental procedures [file mmc1.pdf]

Stem Cell Reports, Volume *18*

Supplemental Information

Spatiotemporal cell junction assembly in human iPSC-CM models of arrhythmogenic cardiomyopathy

Sean L. Kim, Michael A. Trembley, Keel Yong Lee, Suji Choi, Luke A. MacQueen, John F. Zimmerman, Lousanne H.C. de Wit, Kevin Shani, Douglas E. Henze, Daniel J. Drennan, Shaila A. Saifee, Li Jun Loh, Xujie Liu, Kevin Kit Parker, and William T. Pu

Supplemental Materials

Supplemental Figures

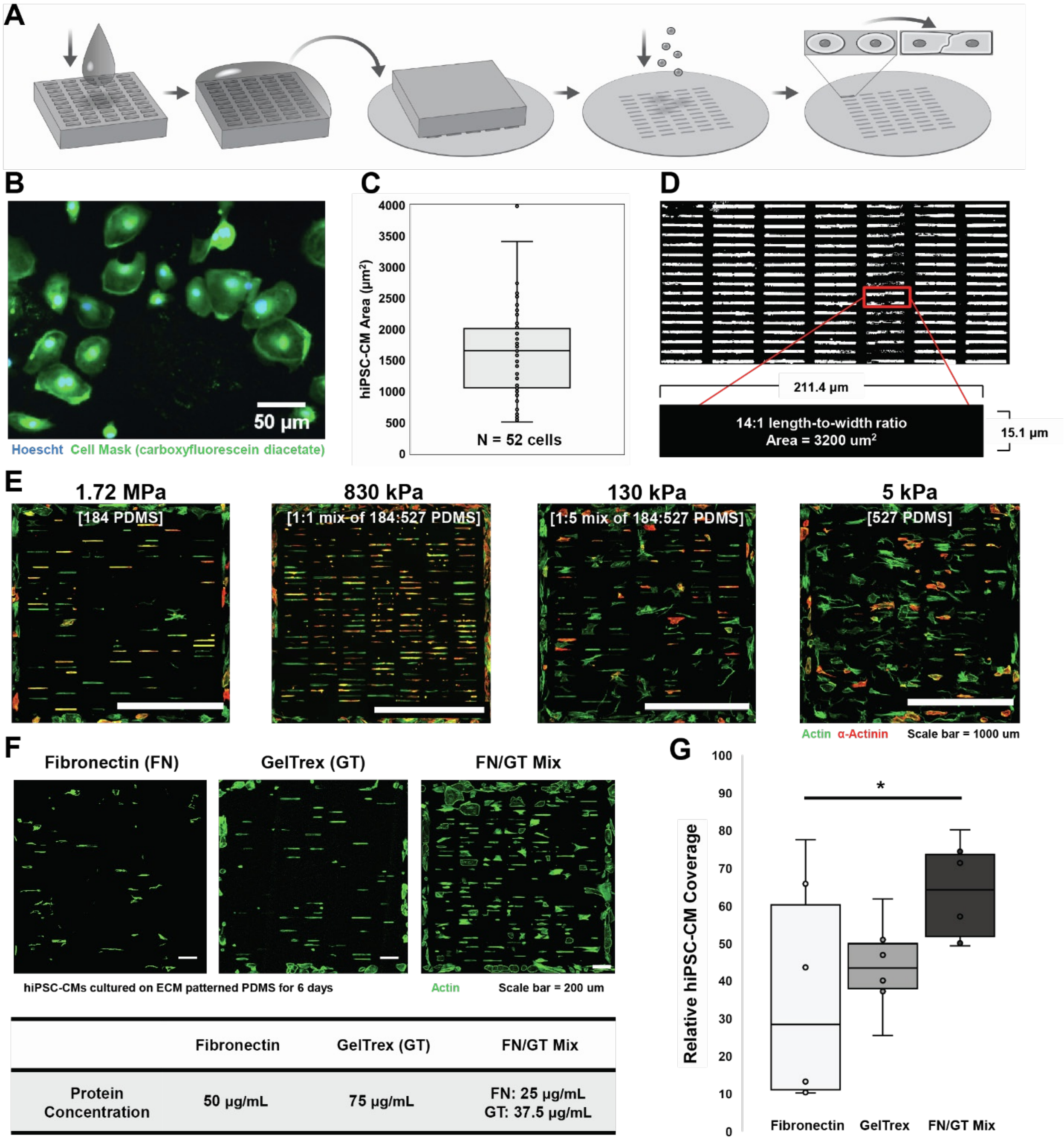

Figure S1. Shape-controlled cell pair substrates for hiPSC-CMs. Related to Fig. 1. (A) Graphical overview of the process of microcontact printing to induce cell pair formation. ECM protein coated PDMS stamps are patterned onto PDMS coverslips containing and array of 14:1 aspect ratio rectangles. (B) Representative image of WT hiPSC-CMs grown on PDMS coated coverslips for 6 days post differentiation, stained with a cell mask and Hoescht for size analysis. (C) hiPSC-CM cell area analysis shows an average size of 1600 µm^2. N = 52 cells across 6 samples. (D) Representative image of a microcontact printed substrate stained with fibronectin with dimensions from CAD (computer-aided designs) images. (E) Representative coverage images using 4 different combinations of 184 PDMS and 527 PDMS with varying substrate stiffnesses. (F) Representative fluorescent images of hiPSC-CMs on microcontact printed substrates with various extracellular matrix proteins and their concentrations. (G) Cell coverage calculated as the total fluorescent actin area, relative to theoretical fibronectin printed values from the CAD design. N = 6 coverslips; > 100 field of views; 2 batches; *$P < 0.05$ by one-way ANOVA.

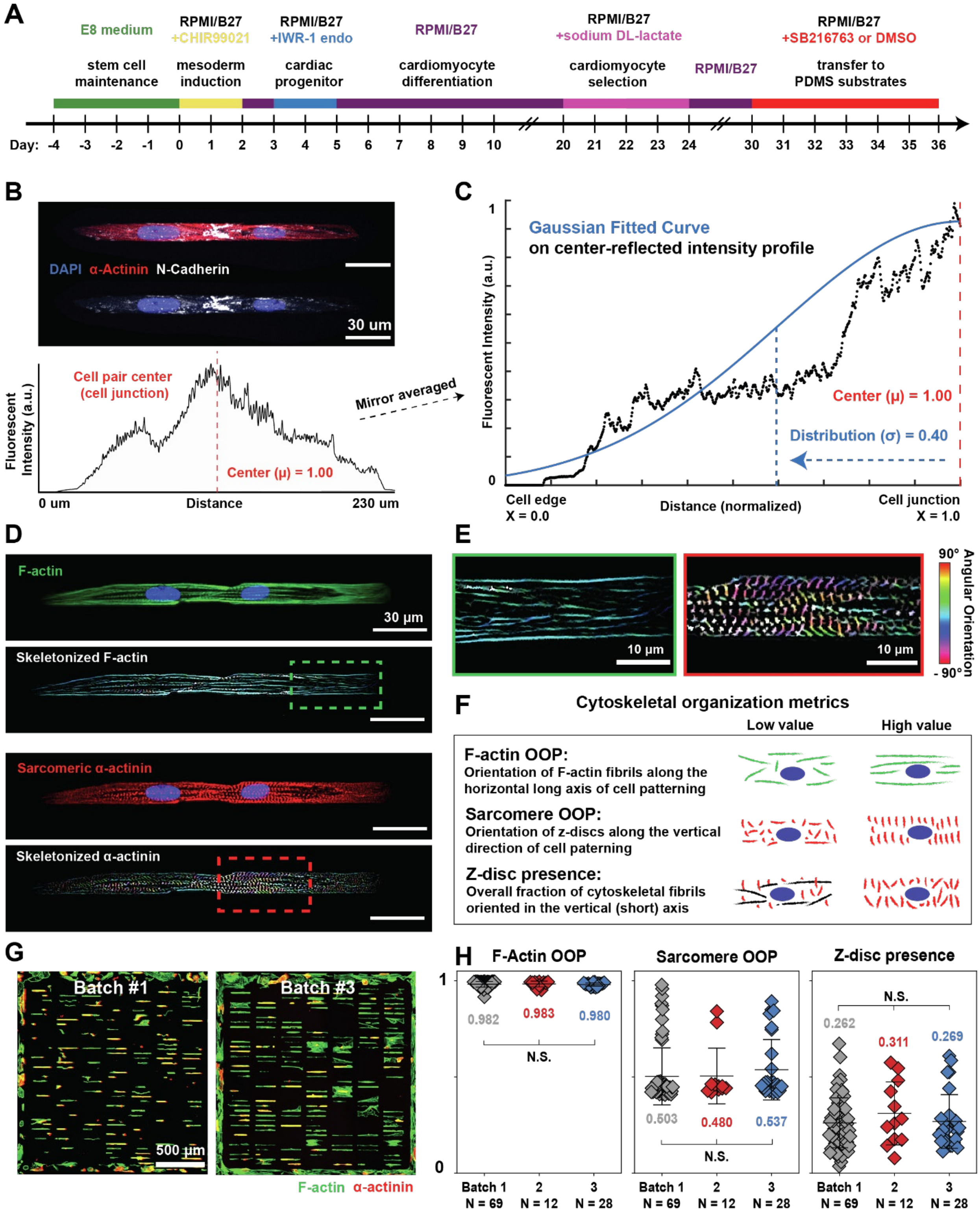

Figure S2. Quantitative metrics for assessing hiPSC-CM cell pair structural quality. Related to Fig. 1. (A) Schematic representation of the iPSC to iPSC-CM differentiation timeline and cell culture conditions. (B) Representative image of a cell pair immunostained for α-actinin (red), N-cadherin (white), and Nuclei (DAPI, blue). Graph below indicates the corresponding fluorescent intensity plotted across the cell pair with distance on the X-axis. (C) To calculate cell junction protein localization center and distribution, the normalized intensity profile for each cell pair was averaged across each cell pair. A Gaussian curve was fitted on the intensity profile to calculate the peak center (μ) and width (σ), defined as the Gaussian standard deviation. (D-E) Representative image of a hiPSC-CM cell pair immunostained for F-actin (green), α-actinin (red), and nuclei (DAPI, blue). Computed

skeletonized F-actin or α-actinin images are shown below, pseudo-colored based on angular fibril orientation. (F) Cartoon depicting cytoskeleton organization metrics. (G) Representative images illustrate relative coverages of two different WT cell pair patches. (H) Cytoskeletal organization metrics F-actin OOP, sarcomere OOP, and Z-disc presence calculated per batch for day 6 cell pairs. Cell pair cytoskeletal organization was consistent regardless of number of cells attached onto a substrate across multiple batches. Mean ± SD with statistically significant P-values (*$P<0.05$) indicated by one-way ANOVA followed by Tukey's HSD test.

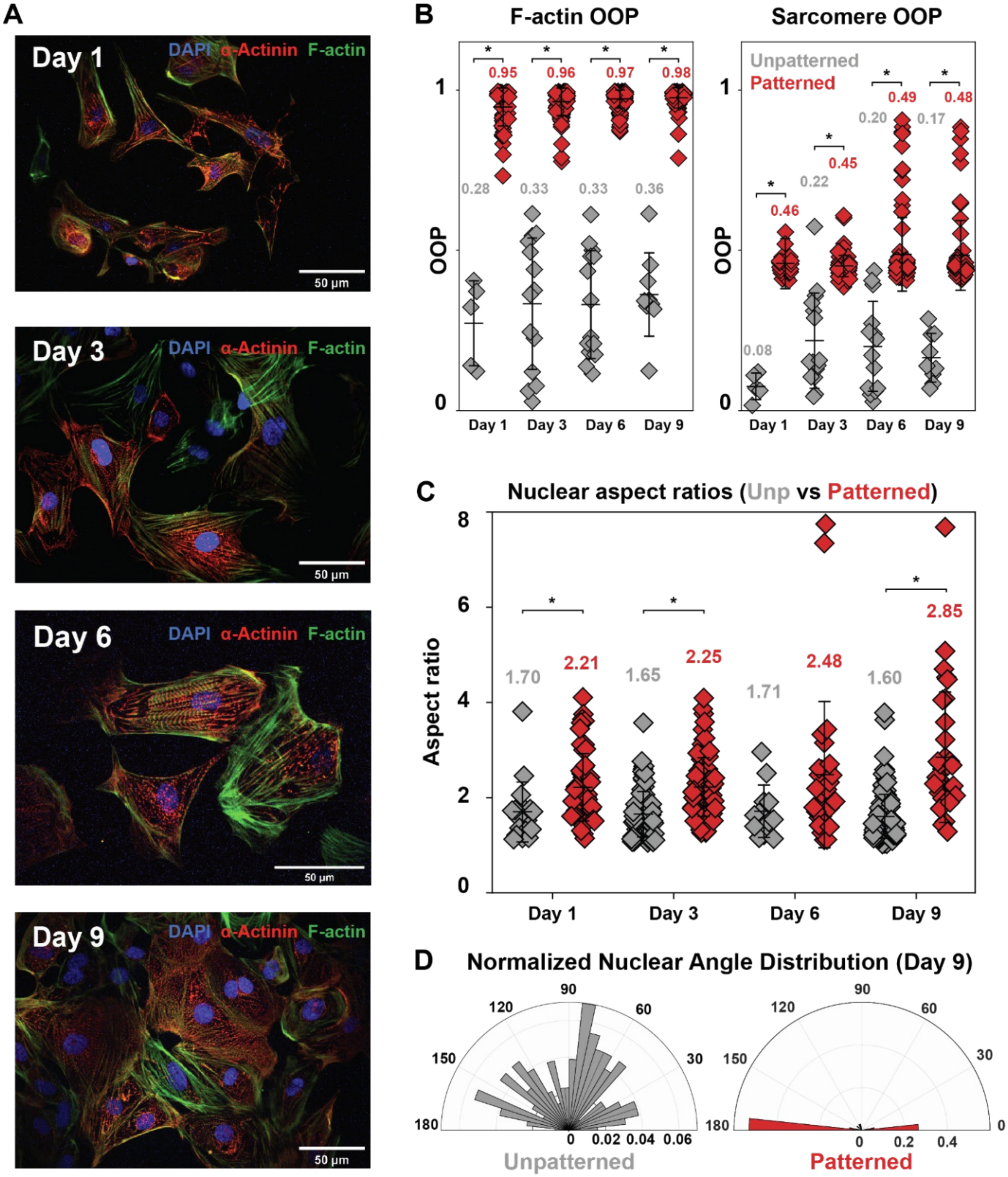

Figure S3. Pleomorphic structure of unpatterned hiPSC-CMs. Related to Fig. 1. (A) Representative unpatterned hiPSC-CMs were immunostained for F-actin (green), α-actinin (red), and nuclei (DAPI, blue) at day 1, 3, 6, or 9 on PDMS substrates. Scale bar, 50 μm. (B) F-actin and sarcomeric α-actinin OOP values of WT hiPSC-CMs without shape constraint (unpatterned, grey) or with constraint into cell pairs (patterned, red; WT data from figure 3). Patterned cell pairs had significantly greater organization at all time points examined. (C) Nuclear aspect ratios of pleomorphic unpatterned hiPSC-CMs and shape-controlled hiPSC-CM pairs over 9 days. (D) Nuclei of patterned cell pairs were aligned with the horizontal axis (the cell long axis), whereas nuclei of unpatterned cells did not exhibit a predominant nuclear orientation. Nuclear angle distribution was measured at day 9. Plots show mean ± SD with statistically significant P-values (*$P<0.05$) indicated by two-way ANOVA followed by Tukey's multiple comparisons test. N = 5, 15, 14, 9 field of views for UNP and 54, 86, 103, 69 cell pairs for patterned cells on days 1, 3, 6, and 9.

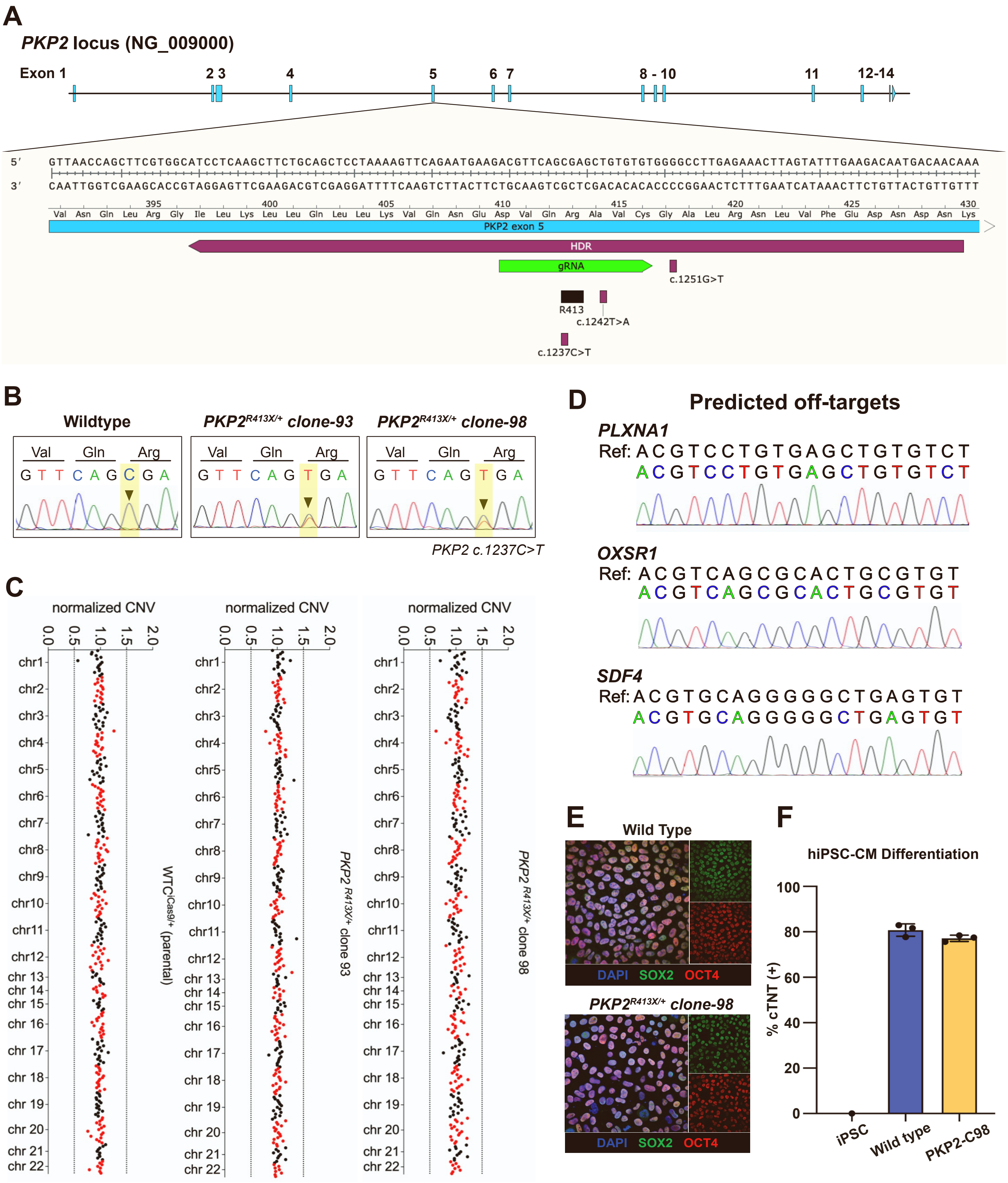

A
PKP2 locus (NG_009000)
Exon 1
2 3
4
5
6 7
8 - 10
11
12-14
PKP2 exon 5
HDR
gRNA
c.1251G>T
R413
c.1242T>A
c.1237C>T
B
Wildtype
PKP2R413X/+ clone-93
PKP2R413X/+ clone-98
Val Gln Arg
PKP2 c.1237C>T
C
normalized CNV
WTCiCas9/+ (parental)
PKP2 R413X/+ clone 93
PKP2 R413X/+ clone 98
D
Predicted off-targets
PLXNA1
Ref: ACGTCCTGTGAGCTGTGTCT
ACGTCCTGTGAGCTGTGTCT
OXSR1
Ref: ACGTCAGCGCACTGCGTGT
ACGTCAGCGCACTGCGTGT
SDF4
Ref: ACGTGCAGGGGGCTGAGTGT
ACGTGCAGGGGGCTGAGTGT
E
Wild Type
PKP2R413X/+ clone-98
DAPI SOX2 OCT4
F
hiPSC-CM Differentiation
% cTNT (+)
iPSC
Wild type
PKP2-C98

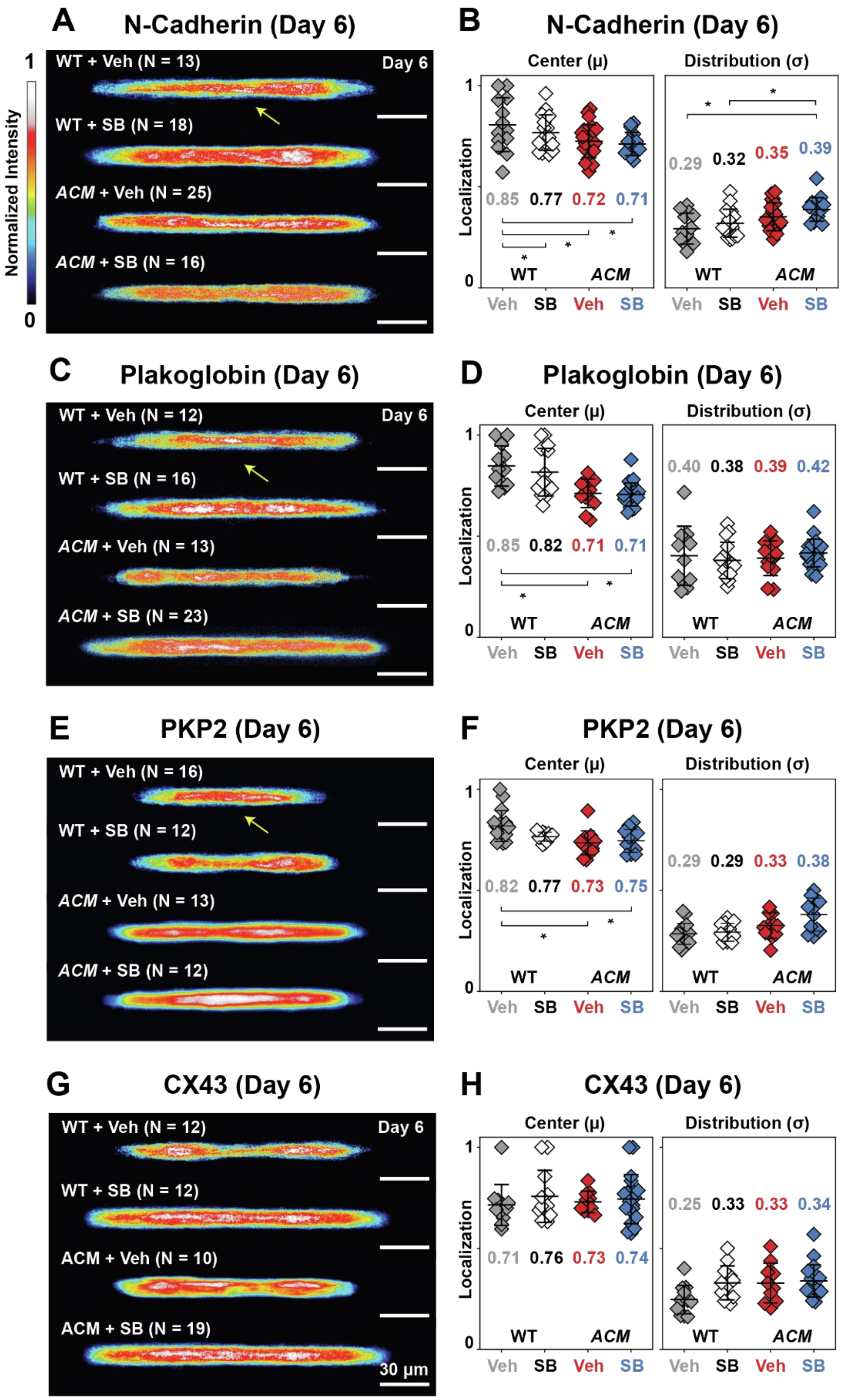

Figure S5. Effect of SB216763 treatment on WT and *ACM* hiPSC-CM pairs on day 6. Related to Fig. 5. WT or *ACM* hiPSC-CM pairs were treated with SB216763 or DMSO for 6 days. (A, C, E, G) Average localization heatmaps for the indicated junctional proteins on day 6. Yellow arrows indicate junctional localization and N, number of cell pairs analyzed over 3-5 independent differentiation batches. Quantitative analysis of N-cadherin (B), plakoglobin (E), and CX43 (F) localization at day 6 of culture with or without SB216763. Plots show mean ± SD. Statistically significant P-values (*P<0.05, by two-way ANOVA including Tukey's multiple comparisons test) are indicated.

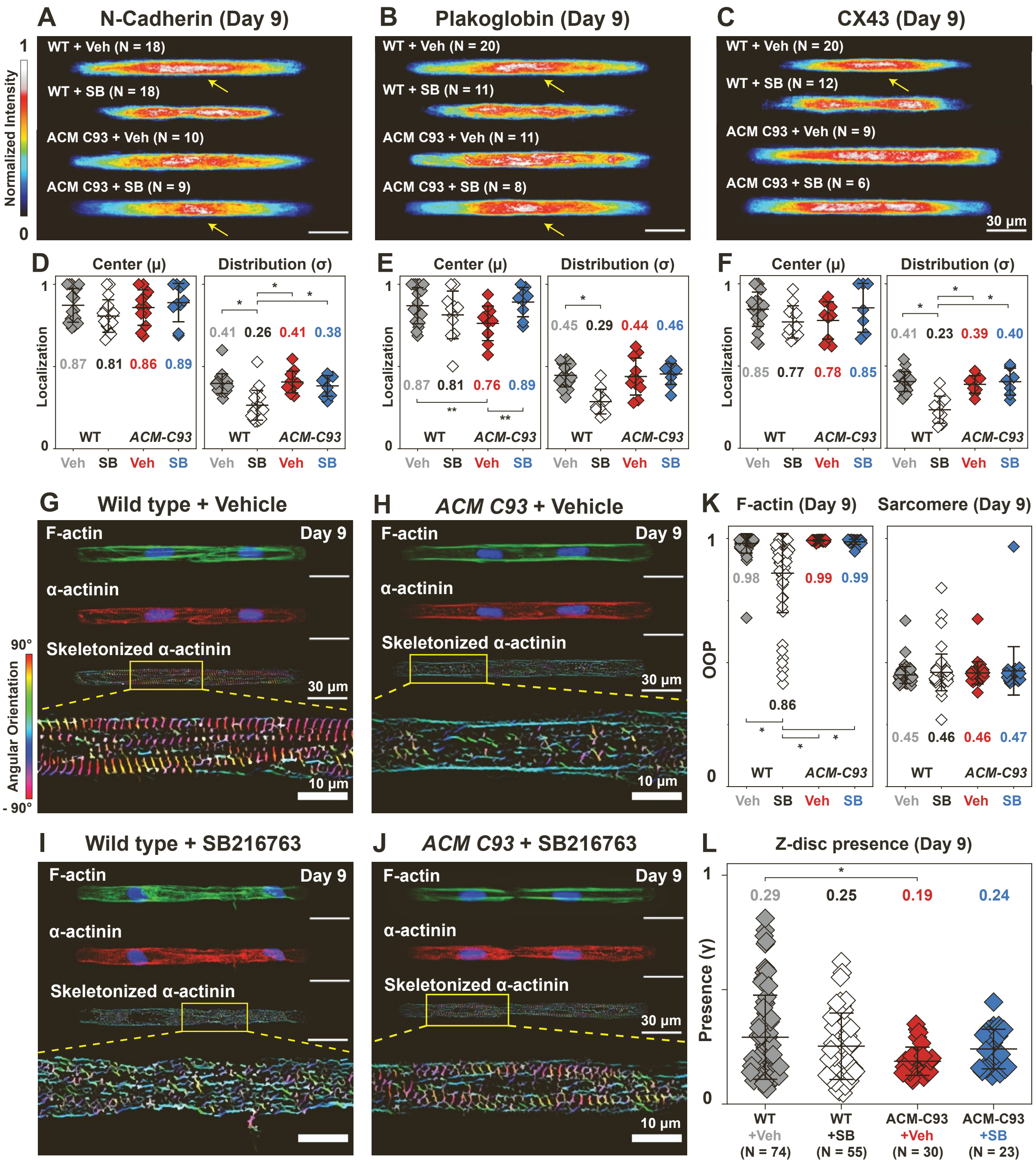

Figure S6. Analysis of hiPSC-CM pairs from an independent *PKP2*$^{R413X/+}$ ACM hiPSC line (clone 93). Related to Fig. 5. To validate observations made in the main *ACM* line (*PKP2*$^{R413X/+}$ clone 98), we studied a second line, clone 93. Measurements were made on day 9. WT data is from Figure 4 and 5. (A-C) Average localization heatmaps for the indicated junctional proteins. N, number of cell pairs analyzed. (D-F) Quantitative analysis of N-cadherin (D), Plakoglobin (E), and CX43 (F) localization. (G-J) Representative images of cell pairs stained for F-actin (green) and α-actinin (red). Images below show corresponding skeletonized fibrils, pseudo-colored based on angular orientation. (K-M) Quantified cytoskeletal structural metrics. Consistent with findings for *ACM clone 98*, SB216763 improved sarcomere OOP and Z-disc presence in *ACM clone 93*. Graphs show mean ± SD. Statistically significant P-values (*P<0.05 by two-way ANOVA including Tukey's multiple comparisons test) are indicated.

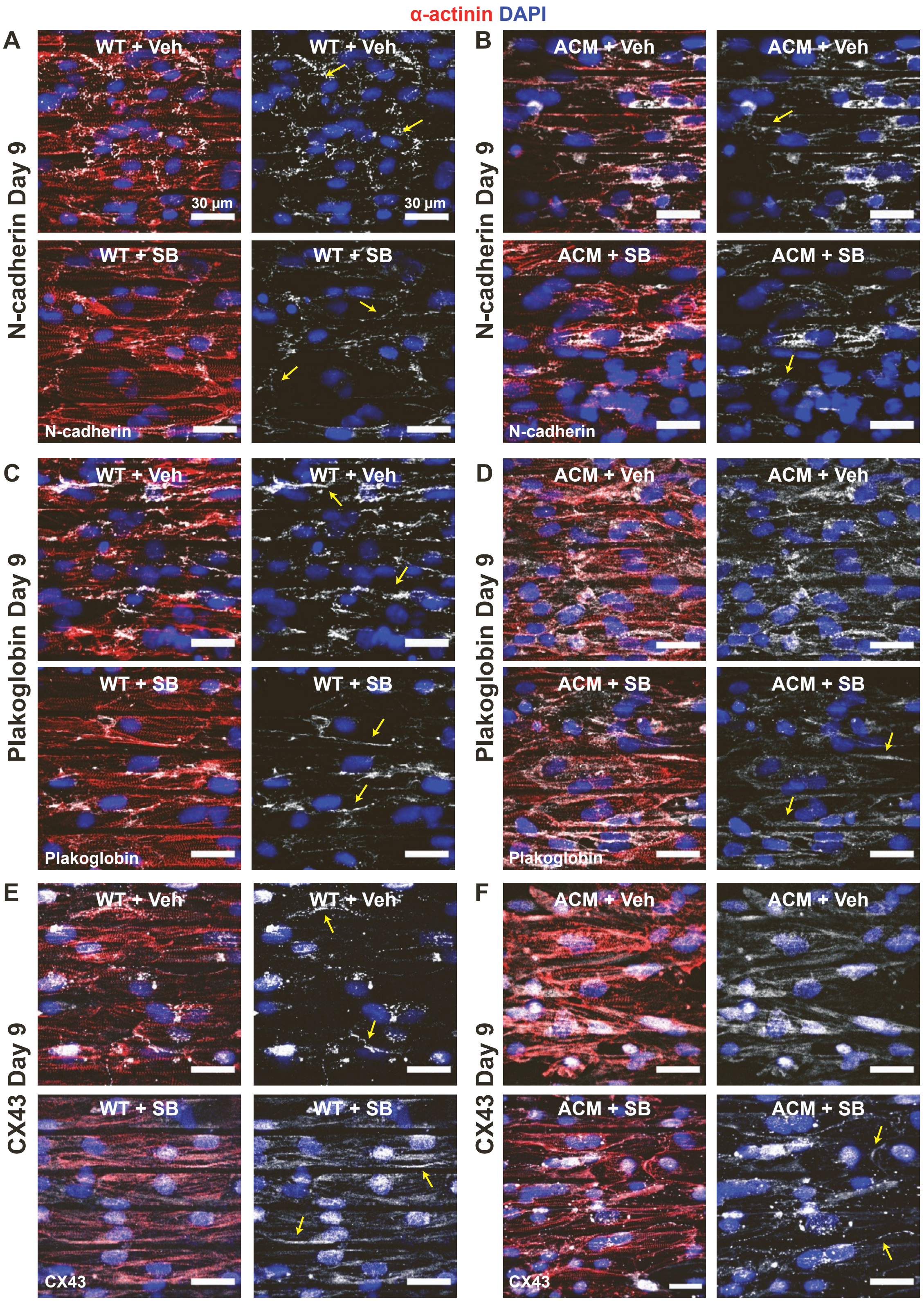

75 **Figure S7. hiPSC-CM tissues immunostained for junctional proteins. Related to Fig. 7.** (A-F) Representative images of day 9 hiPSC-CM tissues immunostained for sarcomeric α-actinin (red), nuclei (DAPI blue), N-cadherin (white) in WT (A) and *ACM* (B), plakoglobin (white) in WT (C) and *ACM* (D), CX43 (white) in WT (E) and *ACM* (F) with vehicle (upper panels) and SB216763 treatment (lower panels). Scale bar represents 30 µm, and yellow arrows indicate distinct plaques of protein expression on cell edges.

Target	Purpose	Direction	Sequence
PKP2 c.1237C>T	guide RNA	--	5'TTCTAATACGACTCACTATAGACGTTCAGCGA GCTGTGTGT GTTTTAGAGCTAGA-3'
PKP2 c.1237C>T	donor template	--	5'TGTTGTCATTGTCTTCAAATACTAAGTTTCTCA AGGCTCCACACACTGCTCACTGAACGTCTTCAT TCTGAACTTTTAGGAGCTGCAGAAGCTTGAGGAT-3'
PKP2^{R413}	genotyping	For	5'-GAAAAAGAGATGATGTAAGGCATCTGG-3'
		Rev	5'-GTTGTGCTACACATAGTTGTTGATGA-3'
OXSR1	off-target	For	5'-TTGGTCGCCATGCTTAGTGT-3'
		Rev	5'-CCTCGCCTCCTGCTCCTC-3'
PLXNA1	off-target	For	5'-CAGGGTCACCAGATCTGCC-3'
		Rev	5'-CTGGGCGGATGGGGAGAC-3'
SDF4	off-target	For	5'-AGCAGACAGGGAGGGGTG-3'
		Rev	5'-GGCACTGCCTCTGGGTGT-3'

Table S1. Nuclei acid sequences used for gene editing, genotyping, and analysis of off-targets. Related to Fig. 2. *PKP2*^{R413X} is caused by a single nucleotide polymorphism at *PKP2 c.1237C>T.* The underlined sequence in the guide RNA represents the target sequence for gene editing which is flanked by the T7 promoter (5'-) and the SpCas9 scaffold (-3') sequence. The donor template is designed antisense to the *PKP2* locus and harbors three mutations: (1) the pathogenic *PKP2 c.1237C>T* mutation (red), (2) a silent mutation at *PKP2 c.1242T>A* (blue) to create a unique BstαI restriction enzyme site, and (3) a silent mutation at *PKP2 c.1251G>A* to ablate the guide RNA protospacer motif (green). *PKP2*^{R413X} primers amplify a 400bp fragment of *PKP2* exon 5 flanking the region targeted for gene editing. Primers for *OXSR1, PLXNA1,* and *SDF4* amplify 300-400bp fragments at predicted off target sites.

Supplemental Experimental Procedures

hiPSC-CM differentiation and culture

WTC-11 hiPSCs were obtained from the Coriell Institute (cat# GM252526) and genetically modified to express a doxycycline-inducible SpCas9 transgene in the AAVS1 safe harbor locus (called WTC-Cas9). In brief, the WTC-Cas9 line was created by transfecting pAAVS1-PDi-CRISPRn (Addgene #73500) and pSpCas9(BB)-2A-GFP (PX458) (Addgene #48138) in WTC-11 hiPSC and selecting for GFP positive clones with successful transgene integration. hiPSCs were maintained in Essential 8 (E8) medium (A1517001, Thermo Fisher). Isogenic hiPSCs harboring the pathogenic *PKP2*$^{R413X/+}$ (*PKP2 c.1237C>T*) variant were engineered using the CRISPR-Cas9 genome editing system as described previously [1]. In brief, a guide RNA (gRNA) was designed to target exon 5 of the human *PKP2* locus (Table S1) and *in vitro* transcribed using the EnGen sgRNA synthesis Kit (NEB, E3322S). A 100-nt donor template was then designed (Table S1) anti-sense to the gRNA and containing three sequence alterations: (1) the pathogenic *PKP2 c.1237C>T* variant, (2) a silent change at *PKP2 c.1242T>A* to create a unique BstαI restriction site for positive clone screening and (3) a silent variant at *PKP2 c.1251G>T* to prevent cleavage by the gRNA. At 50% confluency, Cas9 expression was induced in WTC-Cas9 hiPSCs by supplementing culture media with 2 µg/ml doxycycline (Sigma, D3072) for 18 hours. WTC-Cas9 hiPSCs were then electroporated with 5 µg purified gRNA and 5 µg donor template using an Amaxa Nucleofector system (Lonza). At 48 hours post-transfection, cells were sparsely seeded on a 10 cm plate for colony expansion and selection. Positive clones were verified using Sanger and amplicon sequencing. Potential gRNA off-targets were predicted using CHOPCHOP (https://chopchop.cbu.uib.no) [2] and three of the top predicted exon-coding off-targets were assessed by Sanger sequencing (Table S1). hiPSC pluripotency was verified by expression of OCT4 and SOX2. Karyotypes were assessed by copy number variation using the nCounter Human Karyotype Panel (NanoString Technologies).

To induce cardiomyocyte differentiation, we used WNT modulation [3] (Figure S2 A). hiPSCs were grown to 40-50% confluency. To induce differentiation, on differentiation day 0 (dd0) hiPSCs were cultured in RPMI 1640 medium (61870127, Thermo Fisher) supplemented with B27 minus Insulin (A1895601, Thermo Fisher) and 6 µM CHIR99021 (72054, StemCell Technologies). Cells were switched to RPMI/B27 and then RPMI/B27 supplemented with 5 µM IWR-1 endo (72564, StemCell Technologies) on dd2 and dd3, respectively. On dd5, cells were switched to RPMI/B27. On dd20, hiPSC-CMs underwent negative selection using 5 mM sodium DL-lactate (L7900, Sigma-Aldrich) for 2 – 4 days. After 30 days of differentiation, cells were dissociated by incubating with Accutase (7920, StemCell Technologies) for 30 minutes at 37°C. Cells were resuspended and pooled in equal volumes of Cardiomyocyte Support Medium (5027, StemCell Technologies), centrifuged, and resuspended for counting. Cells were then seeded onto PDMS coated shape-controlled substrates. For experiments involving SB216763 treatment, hiPSC-CMs were seeded and cultured in RPMI/B27 supplemented with 2.5 µM SB216763 or vehicle (DMSO) daily for 6 or 9 days.

PDMS substrate fabrication and cell pair microcontact printing

Microcontact printing for inducing cell pair formation has been reported previously [4–6]. Here we modified and optimized the protocols to tailor them for hiPSC-CMs (Figure S1). Photolithography masks with the desired pattern (array of 14:1 rectangles 211 µm x 15 µm for cell pairs and an array of 25 µm x 4 µm lines for engineered tissues) were drawn up in computer-aided design (CAD) format and converted to photomasks (Outputcity). A thin layer of SU-8 3005 photoresist was spin coated onto silicon wafers (Wafer World) and selectively polymerized by UV-light through the photomasks. The exposed wafers were developed using propylene glycol methyl ether acetate (PGMEA, Sigma). Polydimethylsiloxane (PDMS, Sylgard 184; Dow Corning) was cast and cured on the silicon wafers to create PDMS stamps for microcontact printing. PDMS coated coverslips were prepared by spin coating a 1:1 mix of 184 PDMS (10:1 base to curing agent) and 527 PDMS (1:1 part A and part B) onto glass coverslips. PDMS coated coverslips were treated for 8 mins in a UV Ozone cleaner (Jelight) immediately before microcontact printing with PDMS stamps incubated for an hour with fibronectin (FN) (50 µg/mL, BD Biosciences), LDEV-free GelTrex (GT) (1:200 dilution from stock, A1413302, Thermo Fisher), or a 1:1 mixture of these solutions. PDMS stamps upon contact were then removed, and the coverslips were incubated with room temperature 1% (w/v) Pluronic F-127 (BASF) for 10 minutes to prevent cell adhesion on unpatterned regions (Figure S1 D). After washing 3 times with phosphate-buffered saline (PBS, Invitrogen), coverslips were ready for cell plating. Fully dissociated hiPSC-CMs were then seeded at a density of 1E5 cells/ml in a 12 well plate.

Engineered tissue fabrication

Micro-molded gelatin tissues were fabricated by modification of previously published protocols for fabrication of gelatin muscular thin films [7]. A double layer of laboratory tape (General-Purpose Laboratory Labeling Tape, VWR) was affixed to an 18 mm square glass coverslip (Corning) or 1 mm thick Clear Scratch- and UV-Resistant Acrylic Sheet (McMaster-Carr) and an array of three tissue outlines (Figure 5 A) were laser cut with an Epilog Mini laser. The tissue chips were removed with tweezers, submerged in bleach for 10 minutes and washed with 70% ethanol for 30 min to remove any remaining residue. The chips were then washed with PBS three times and dried. A solution of 20% w/v gelatin (Sigma Aldrich) and 8% w/v microbial transglutaminase (MTG, Ajinomoto) were prepared with PBS and dissolved for 30 min at 65°C or 37°C, respectively. 5 ml of each solution (final concentration 10% w/v or 4% w/v total, respectively) were mixed and a 300 μl aliquot of the mixture was pipetted onto each tissue chip. PDMS stamps containing lines of 25 mm wide ridges, 4 mm grooves, and 5 mm groove depth were placed on top of the gelatin mixture aliquot and lightly pressed down with a 200 g scale calibration weight. These tissue chips were stored overnight at room temperature to cure, inside a glass container to prevent full dehydration. Then the calibration weights were removed, and the tissue chips were immersed in PBS for 30 min for rehydration and easy removal of PDMS stamps. These tissue chips were then submerged in 70% ethanol for 10 minutes under the UV light. The chips were then rinsed 3 times with PBS again and coated with a 1:100 dilution of GelTrex at 37°C for 1 hr or 4°C overnight. Fully dissociated hiPSC-CMs were then seeded at a density of 1E6 cells/ml for a total of 2E6 cells per well in a 12 well plate.

Ca^{2+} optical mapping and propagation velocity calculation

To prepare tissue substrates for Ca^{2+} optical mapping, day 6 and day 9 samples were incubated with 2 μM X-Rhod-1 AM (Invitrogen, X14210) in RPMI/B27 for 60 minutes at 37°C followed by dye-free media for an additional 15 minutes at 37°C. Relative cytoplasmic Ca^{2+} was imaged using a modified tandem-lens macroscope as described previously [8]. Videos were acquired at 400 frames per second using a high-speed camera (MiCAM Ultima, Scimedia) through a plan APO 0.63x or 1x objective, a collimator (Lumencor) and a 200-mW mercury lamp for epifluoresence illumination (X-Cite exacte, Lumen Dynamics). A filter set (excitation filter: 580/14 nm, dichroic mirror: 593 nm cut-off, emission filter 641/75, Semrock) was used for imaging. Propagation velocities were calculated based on post-processed data using MATLAB (MathWorks) and the MiCAM imaging software (Scimedia) [7]. A spatial filter of 3 x 3 pixels was applied to improve the signal-to-noise ratio, and the activation time of each pixel was calculated by the derivative of the fluorescent intensity (the average maximum upstroke slope) over a 10 second recording window. The propagating velocity was quantified in the narrow 3 to 5 mm region of the tissue while electrically pacing the tissues at 1 Hz with an IonOptix myopacer (10V amplitude, 10 ms pulse width) using two platinum electrodes (Sigma-Aldrich) with 1 mm spacing.

Immunostaining of hiPSC-CM cell pairs and tissues

Substrates were washed with PBS several times before fixation with 4% (v/v) paraformaldehyde with 0.05% (v/v) Triton X-100 in PBS for 10 minutes. To block non-specific binding, samples were incubated with 5% (w/v) bovine serum albumin (BSA) in PBS for 30 minutes. Samples were then incubated with primary antibodies against fibronectin (1:500; ab2413 abcam), sarcomeric α-actinin (1:500; ab9465 abcam), N-cadherin (1:500; ab76057 abcam), γ-catenin (1:500; MAB2083 Millipore), PKP2 (1:500; MABT394 Millipore), or CX43 (1:500; C6219 Sigma), in 1% (w/v) BSA in PBS overnight at 4°C. Alexa Fluor 488, 546, or 633 secondary antibodies (1:200 Invitrogen), DAPI (1:200 Invitrogen), and Alexa Fluor 488 or 633 conjugated Phalloidin (1:200 Invitrogen) were further incubated at room temperature for an hour and washed with PBS before mounting onto microscope slides with Prolong Gold anti-fade agent (Invitrogen). Slides were dried overnight and stored at 4°C until imaging on a spinning disk confocal microscope (Olympus IX83, Andor spinning disk).

Image extraction and Orientational Order (OOP) analysis

Image analysis to quantify OOP and cytoskeletal alignment were conducted with slight modifications to previously published methods [9–11]. Cytoskeletal actin and sarcomere OOP represent the overall angular alignment of processed fibrils, and Z-disc presence represents the fraction of cytoskeletal elements oriented in the orthogonal direction to the actin cytoskeleton (Figure S3 E). Preprocessing of images were performed with ImageJ/FIJI using the tubeness and OrientationJ plugins. Preprocessed images including the orientations of filamentous structures were then collated and the respective calculations conducted through MATLAB (Mathworks) [9].

Intercalated disc junctional localization metrics analysis

Image analysis for creating heatmaps and calculating protein localization were conducted through automated image processing scripts on ImageJ/FIJI and MATLAB [12]. Acquired cell pairs expressed sarcomeric α-actinin, a cardiomyocyte marker, covered most of the patterned area, and contained two distinct nuclei. Cell junction markers were not used to decide to include or exclude a cell pair. Suspected binucleated cells were excluded based on the criteria of nuclei being less than 50 µm apart, with horizontal cytoskeletal filaments spanning across both nuclei. Cell pairs were first automatically rotated and centered based on their fluorescent actin channel. For creating averaged heatmaps, auto-contrasting was applied across stacks of cell pairs and converted into a composite that depicted the average localization and distribution of a select protein (Figure S2 C). Sum Z-projections were then applied and pseudo-colored using a Royal lookup table where white represents completely saturated pixels. For calculating numerical quality metrics ranging from 0 to 1, individual fluorescent profiles of each channel were exported and analyzed separately. The 2D fluorescent intensity profile of the cell pair was split into a profile for each cell and averaged, with distance 0 representing the cell edge and 1 representing the cell junction (Figure S3 A and B). A Gaussian curve was then fitted on top of the averaged per cell intensity profile to extract values for the center of the curve and one standard deviation (the distribution/width). Calculated values for intensity center and distribution were tabulated and graphed.

Capillary westerns with subcellular fractionation for protein localization

Two million hiPSC-CMs were cultured in monolayers and treated with vehicle (DMSO) or 2.5 µM SB216763. On day 9, cells were harvested using Accutase and sub-cellular fractionation was performed using the Subcellular Protein Fractionation Kit (Thermo #78840). Protein samples (400 ng/lane) were analyzed by capillary western (ProteinSimple Wes device). Primary antibodies used were against N-cadherin (Invitrogen#33-3900, 1:100); plakoglobin (Millipore #MAB2083, 1:200); PKP2 (Millipore #MABT394, 1:300); CX43 (Abcam #ab11370, 1:400); GAPDH (Thermo# PA1-16777, 1:400); β-catenin (Abcam #ab32572, 1:400); Lamin B1 (Cell signaling #12586S, 1:1000); SERCA2 (Cell Signaling Technology #9580S, 1:400). Blots were normalized with respect to loading controls, GAPDH for cytosolic, LMNB1 for nuclear, and SERCA2 for membrane fractions. N-cadherin (CDH2) undergoes a series of post-translational modifications in its way to the cell membrane. Both 80 kDa and 100 kDa bands represent CDH2 still undergoing post-translational modification. Mean intensity values for both bands were combined before normalization to respective GAPDH bands.

Quantitative RT-PCR

hiPSC-CMs were seeded into 48-well plates at 100,000 cells per well and maintained for one week in RPMI/B27 medium. First-strand cDNA synthesis was then performed directly from cells using the FastLane Cell cDNA kit (215011, Qiagen) per manufacturer's instructions. Quantitative RT-PCR (qRT-PCR) was performed using Power SYBR Green PCR Master Mix (4368708, ThermoFisher) per manufacturer's instructions on a BioRad CFX384 qPCR Real-Time PCR machine. Relative levels of PKP2 transcripts were determined using the comparative C(T) method [13] and normalized to levels of GAPDH. Gene-specific primers used for qRT-PCR are found in Table S1.

Statistical analysis

Bar graphs indicate mean ± SD. Box plots represent the interquartile range and median (box and center line) and 1.5 times the interquartile range (whiskers). Statistical analysis across calculated values were conducted using one-way ANOVA or two-way ANOVA followed by Tukey's HSD test or test for multiple comparisons. Statistically significant P-values ($P<0.05$ and $P<0.1$) are indicated within the graphs where appropriate.

Supplemental References

1. Wang G, Yang L, Grishin D, et al. Efficient, footprint-free human iPSC genome editing by consolidation of Cas9/CRISPR and piggyBac technologies. *Nat Protoc*. 2017;12(1):88-103. doi:10.1038/nprot.2016.152
2. Geiss GK, Bumgarner RE, Birditt B, et al. Direct multiplexed measurement of gene expression with color-coded probe pairs. *Nat Biotechnol*. 2008;26(3):317-325. doi:10.1038/nbt1385
3. Lian X, Zhang J, Azarin SM, et al. Directed cardiomyocyte differentiation from human pluripotent stem cells by modulating Wnt/β-catenin signaling under fully defined conditions. *Nat Protoc*. 2013;8(1):162-175. doi:10.1038/nprot.2012.150
4. McCain ML, Lee H, Aratyn-Schaus Y, Kléber AG, Parker KK. Cooperative coupling of cell-matrix and cell-cell adhesions in cardiac muscle. *Proc Natl Acad Sci U S A*. 2012;109(25):9881-9886. doi:10.1073/pnas.1203007109
5. Aratyn-Schaus Y, Pasqualini FS, Yuan H, et al. Coupling primary and stem cell-derived cardiomyocytes in an in vitro model of cardiac cell therapy. *J Cell Biol*. 2016;212(4):389-397. doi:10.1083/jcb.201508026
6. McCain ML, Desplantez T, Geisse NA, et al. Cell-to-cell coupling in engineered pairs of rat ventricular cardiomyocytes: relation between Cx43 immunofluorescence and intercellular electrical conductance. *Am J Physiol - Hear Circ Physiol*. 2012;302(2). doi:10.1152/ajpheart.01218.2010
7. Lee KY, Park SJ, Matthews DG, et al. An autonomously swimming biohybrid fish designed with human cardiac biophysics. *Science (80-)*. 2022;375(6581):639-647. doi:10.1126/science.abh0474
8. Park SJ, Zhang D, Qi Y, et al. Insights into the Pathogenesis of Catecholaminergic Polymorphic Ventricular Tachycardia from Engineered Human Heart Tissue. *Circulation*. 2019;140(5):390-404. doi:10.1161/CIRCULATIONAHA.119.039711
9. Pasqualini FS, Sheehy SP, Agarwal A, Aratyn-Schaus Y, Parker KK. Structural phenotyping of stem cell-derived cardiomyocytes. *Stem Cell Reports*. 2015;4(3):340-347. doi:10.1016/j.stemcr.2015.01.020
10. Sheehy SP, Pasqualini F, Grosberg A, Park SJ, Aratyn-Schaus Y, Parker KK. Quality metrics for stem cell-derived cardiac myocytes. *Stem Cell Reports*. 2014;2(3):282-294. doi:10.1016/j.stemcr.2014.01.015
11. Wang G, McCain ML, Yang L, et al. Modeling the mitochondrial cardiomyopathy of Barth syndrome with induced pluripotent stem cell and heart-on-chip technologies. *Nat Med*. 2014;20(6):616-623. doi:10.1038/nm.3545
12. Kim S. Cell pair heatmap overlay and intensity profile extraction/fitting. Published online September 28, 2022. doi:10.5281/ZENODO.7120682
13. Schmittgen TD, Livak KJ. Analyzing real-time PCR data by the comparative CT method. *Nat Protoc*. 2008;3(6):1101-1108. doi:10.1038/nprot.2008.73
