# Supplementary material for: Spatiotemporal cell junction assembly in human iPSC-CM models of arrhythmogenic cardiomyopathy
Source: Stem Cell Reports. 2023 Aug 17;18(9):1811–26. doi: 10.1016/j.stemcr.2023.07.005 (PMC10545490; doi:10.1016/j.stemcr.2023.07.005)
Supplement: Document S2. Article plus supplemental information [file mmc5.pdf]

Stem Cell Reports
Article
OPEN ACCESS

Spatiotemporal cell junction assembly in human iPSC-CM models of arrhythmogenic cardiomyopathy

Sean L. Kim,[1,2] Michael A. Trembley,[2] Keel Yong Lee,[1,3] Suji Choi,[1] Luke A. MacQueen,[1] John F. Zimmerman,[1] Lousanne H.C. de Wit,[1] Kevin Shani,[1,2] Douglas E. Henze,[1] Daniel J. Drennan,[1] Shaila A. Saifee,[2] Li Jun Loh,[2] Xujie Liu,[2] Kevin Kit Parker,[1,2,4,5,*] and William T. Pu[2,4,*]
[1]Disease Biophysics Group, John A. Paulson School of Engineering and Applied Sciences, Harvard University, Boston, MA 02134, USA
[2]Department of Cardiology, Boston Children's Hospital, Boston, MA 02115, USA
[3]Department of Integrative Bioscience and Biotechnology, Sejong University, Seoul 05006, Republic of Korea
[4]Harvard Stem Cell Institute, Harvard University, Cambridge, MA 02138, USA
[5]Wyss Institute for Biologically Inspired Engineering, Harvard University, Boston, MA 02115, USA
*Correspondence: kkparker@g.harvard.edu (K.K.P.), william.pu@cardio.chboston.edu (W.T.P.)
https://doi.org/10.1016/j.stemcr.2023.07.005

SUMMARY

Arrhythmogenic cardiomyopathy (ACM) is an inherited cardiac disorder that causes life-threatening arrhythmias and myocardial dysfunction. Pathogenic variants in Plakophilin-2 (*PKP2*), a desmosome component within specialized cardiac cell junctions, cause the majority of ACM cases. However, the molecular mechanisms by which *PKP2* variants induce disease phenotypes remain unclear. Here we built bioengineered platforms using genetically modified human induced pluripotent stem cell-derived cardiomyocytes to model the early spatiotemporal process of cardiomyocyte junction assembly *in vitro*. Heterozygosity for truncating variant $PKP2^{R413X}$ reduced Wnt/β-catenin signaling, impaired myofibrillogenesis, delayed mechanical coupling, and reduced calcium wave velocity in engineered tissues. These abnormalities were ameliorated by SB216763, which activated Wnt/β-catenin signaling, improved cytoskeletal organization, restored cell junction integrity in cell pairs, and improved calcium wave velocity in engineered tissues. Together, these findings highlight the therapeutic potential of modulating Wnt/β-catenin signaling in a human model of ACM.

INTRODUCTION

The cardiac intercalated disc is a specialized cell junction that mechanically and electrically couples cardiomyocytes. This coupling is enabled by the area composita, a hybrid adhesion and signaling complex, that includes adherens junctions, desmosomes, and gap junctions. Deleterious variants in desmosome genes cause arrhythmogenic cardiomyopathy (ACM), an inherited cardiomyopathy characterized by ventricular arrhythmias and progressive fibrofatty replacement of the myocardium, ultimately leading to heart failure (Austin et al., 2019). ACM affects an estimated 1 in 5,000 people, and more than one-half of ACM patients harbor genetic variants in plakophilin-2 (*PKP2*), a desmosome gene (Corrado et al., 2017; Gerull et al., 2004). Patient samples and animal models have shown that pathogenic *PKP2* variants induce misexpression and mislocalization of other area composita proteins, including plakoglobin and connexin-43 (CX43; official symbol *GJA1*), components of desmosome and gap junctions, respectively. This failure to properly assemble cell junctions is hypothesized to compromise the structural and electrical connectivity across cardiomyocytes and its functions in intracellular signaling, culminating in heart failure (Bhonsale et al., 2015; Cerrone et al., 2017; Cruz et al., 2015; Kirchner et al., 2012; Mazzanti et al., 2016; Oxford et al., 2007; Sato et al., 2009). Unfortunately, treatments for ACM are lacking, in part because of an incomplete understanding of the molecular events linking *PKP2* pathogenic variants to the dynamics of cell junction remodeling, arrhythmogenesis, and myocardial fibrofatty replacement.

At the molecular and cellular levels, mislocalization of plakoglobin is hypothesized to suppress Wnt/β-catenin signaling (Austin et al., 2019; Chelko et al., 2016; Garcia-Gras et al., 2006; Zhurinsky et al., 2000), an evolutionarily conserved pathway that regulates cardiomyocyte gene expression (Gessert and Kühl, 2010; Komiya and Habas, 2008). Previous studies have shown that SB216763, a small molecule that activates Wnt/β-catenin signaling by inhibiting glycogen synthase kinase 3 (GSK-3), restored cell junction integrity, and improved cardiac conduction and ejection fraction in mouse, rat, and zebrafish ACM models (Asimaki et al., 2014; Chelko et al., 2016; Padrón-Barthe et al., 2019). However, the cellular and junctional remodeling processes that occur with Wnt modulation have been difficult to track. Most ACM models including patient samples and animal models represent late-stage disease phenotypes resulting from extended periods of pathological remodeling with innate compensatory mechanisms. To facilitate dissection of the pathogenic role of ACM-causing variants and pathways within human cardiomyocytes, recent studies (Bliley et al., 2021; Inoue et al., 2022; Khudiakov et al., 2020; Kim

© 2023 The Author(s).
This is an open access article under the CC BY license (http://creativecommons.org/licenses/by/4.0/).

et al., 2013; Zhang et al., 2021) have largely focused on using cardiomyocytes differentiated from human induced pluripotent stem cells (hiPSC-CMs).

hiPSC-CMs can recapitulate disease phenotypes (Thomas et al., 2022) and have been used to demonstrate aberrant metabolism, intracellular lipid accumulation, sarcomere assembly, calcium handling, contractility, and electrophysiology in ACM hiPSC-CMs (Austin et al., 2019; Bliley et al., 2021; Inoue et al., 2022; Khudiakov et al., 2020; Kim et al., 2013; Zhang et al., 2021). In this study, we developed a hiPSC-CM cell pair model to study the assembly of cell-cell junctions and their perturbation by a pathogenic *PKP2* nonsense variant, *PKP2 c.1237C>T*, p.R413X. We found that $PKP2^{R413X/+}$ cells exhibited reduced Wnt/β-catenin signaling and altered cytoskeletal organization, which impaired the formation of cell-cell junctions. Stimulation of Wnt/β-catenin by SB216763 reversed the maladaptive structural and functional phenotypes, suggesting that Wnt/β-catenin signaling regulates cell junction dynamics via the cytoskeleton.

RESULTS

Optimized cell pair platform to study hiPSC-CM cell-cell junction formation

The predominance of pathogenic sequence variants in desmosome genes implicates cell-cell junctions in ACM pathogenesis. The minimal cardiac functional unit that forms cell-cell junctions is a cell pair. To study the effect of ACM pathogenic variants on cell-cell junction formation, we optimized our previously described cell pair platform (Aratyn-Schaus et al., 2016; McCain et al., 2012a, 2012b). We micro-contact printed rectangular islands of extracellular matrix (ECM) proteins with a 14:1 length-to-width ratio, which models the dimensions of two human adult cardiomyocytes (average aspect ratio 7:1) connected end to end (Figure S1A). Guided by the area of unpatterned hiPSC-CMs (1,600 ± 101 μm^2, mean ± SEM, Figures S1B and S1C), we used an ECM island area of 3,200 μm^2 for cell pairs (Figure S1D). Initial experiments using substrates optimized for rat cardiomyocyte cell pairs (Aratyn-Schaus et al., 2016; McCain et al., 2012a, 2012b) resulted in low cell coverage. An ECM protein mixture of fibronectin and GelTrex (FN/GT) on soft polydimethylsiloxane (PDMS) substrates improved the consistency and adhesion of hiPSC-CMs by 73% compared with patterned substrates containing only fibronectin (Figures S1E–S1G). These optimizations achieved 64% cell coverage on the printed substrates (Figures S1F and S1G). Together, these data suggest that hiPSC-CMs require a more biomimetic ECM protein mixture to promote cell attachment and survival on engineered substrates *in vitro* in comparison with rat cardiomyocytes.

Next, we examined the formation of cell junctions by hiPSC-CMs in our cell pair platform. hiPSC-CMs were allowed to mature in monolayer culture to post-differentiation day 30 and then replated on FN/GT patterned soft PDMS substrates. Formation of cell-cell junctions was then monitored over the subsequent 9 days (Figure S2A). We focused on N-cadherin, plakoglobin, PKP2, and CX43, components of the intercalated disc (Manring et al., 2018), and used immunostaining to monitor their distribution and localization during cell junction assembly. Consistently across cell pairs, N-cadherin, plakoglobin, and PKP2 staining localized at the cell-cell junction (Figures 1A–1I) by day 6, suggesting development of adhesive junctions comprising cadherins and desmosomes that mediate mechanical coupling. CX43 immunofluorescence localization at the cell-cell junction was observed at day 9 (Figures 1J–1L), suggesting that gap junction-mediated electrical coupling between cell pairs is established after mechanical coupling.

To summarize the immunostaining results across cell pairs and experimental batches, we took advantage of the reproducible shape and size of cell pairs to generate representative overlaid heatmaps of protein expression at each timepoint. For example, the superimposed immunofluorescence heatmaps showed that N-cadherin (Figure 1B), plakoglobin (Figure 1E), and PKP2 (Figure 1H) staining transition from a diffuse cytoplasmic distribution toward a distinct junctional localization over 6 days in culture. A few days later, CX43 staining consistently localized at cell-cell junctions (Figure 1K). Corresponding quantified values for the center (μ) and width/spread (σ) of each protein distribution (Figures S2B and S2C) further confirmed gradual localization of these junctional proteins to the cell-cell junction (Figures 1C, 1F, 1I, and 1L) over 9 days on the cell pair substrates *in vitro*.

The cytoskeletal network is another critical structure that forms via myofibrillogenesis during the early stages of cardiomyocyte development. Two cytoskeletal proteins, filamentous actin (F-actin) and sarcomere Z-disc protein α-actinin, align along the long and short axes, respectively, as cardiomyocytes develop and mature *in vitro*, and thus indicate the cell's structural health or maturity. We quantified cell pair cytoskeletal and sarcomeric organization using the orientational order parameter (OOP) and Z-disc presence (Figures S2D–S2F), previously established metrics (Pasqualini et al., 2015; Sheehy et al., 2014; Wang et al., 2014). OOP describes the overall orientation of fibrillar structures based on their distribution, with 0 and 1 representing isotropic and anisotropic alignment, respectively, and Z-disc presence representing the overall fraction of Z-discs oriented in the cardiomyocyte short axis (Figure S2F). Since we standardized the cellular microenvironment, these metrics were similar across differentiation

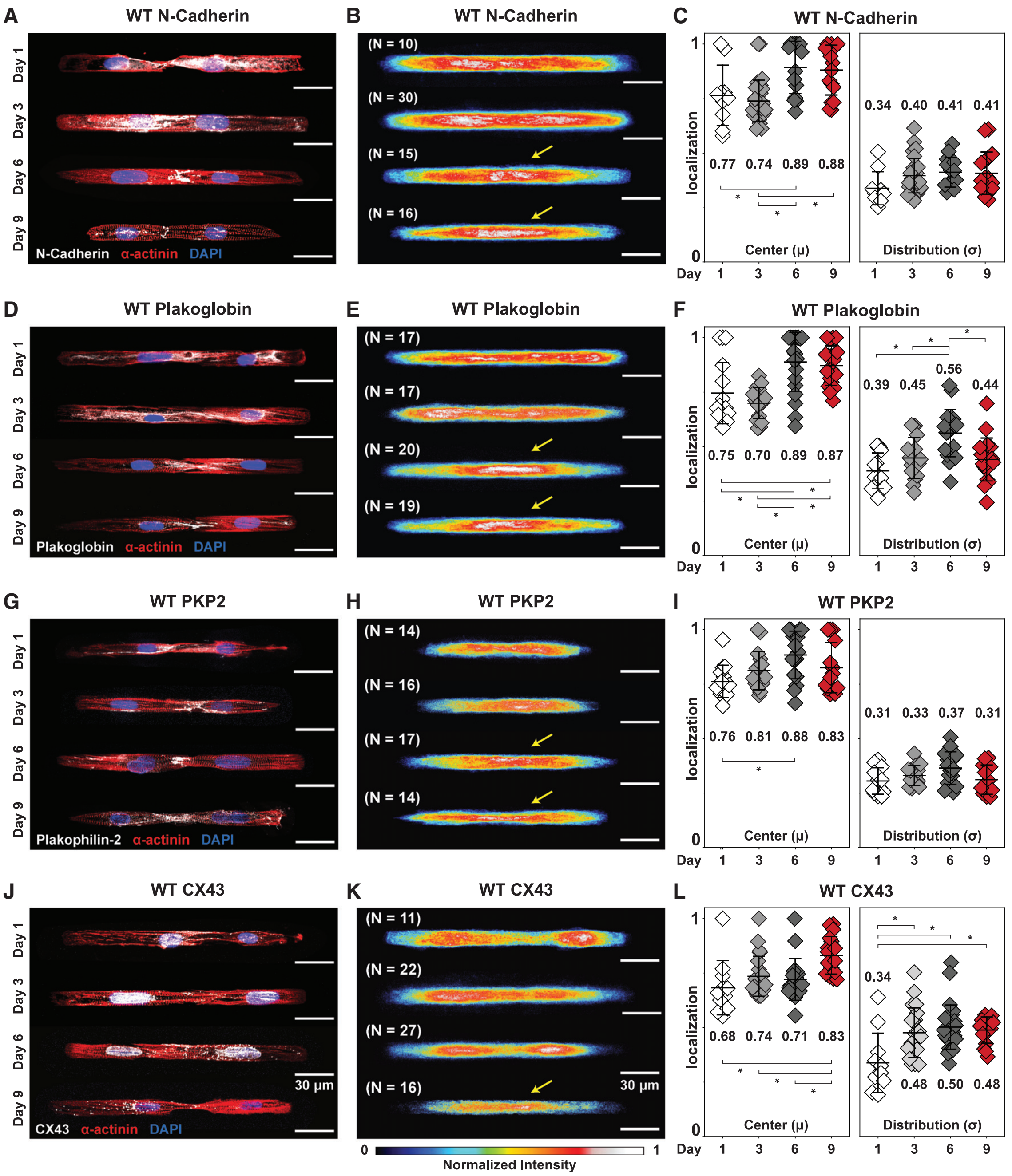

Figure 1. Spatiotemporal cell-cell junction assembly of WT hiPSC-CMs

(A–L) See also Figures S1–S3. Immunofluorescence images, averaged heatmaps, and quantified protein localization of N-cadherin (A–C), plakoglobin (D–F), PKP2 (G–I), and CX43 (J–L). Representative images of WT hiPSC-CM cell pairs stained for nuclei (blue), α-actinin (red), and cell junction proteins (white).

(B, E, H, K) Overlaid heatmaps represent the distribution of individual proteins averaged over all imaged cells for each time point. Yellow arrows indicate junctional localization. Quantified center and distribution of N-cadherin (C), plakoglobin (F), PKP2 (I), and CX43 (L). Mean ± SD. One-way ANOVA followed by Tukey's honest significant difference (HSD) test: $^*p < 0.05$. Cell pairs were aggregated from three to four independent differentiations.

batches with variable plating efficiencies (Figures S2G and S2H).

Compared with unpatterned hiPSC-CMs on FN/GT-coated PDMS, patterned cell pairs exhibited greater F-actin and sarcomeric OOP (Figures S3A and S3B). Cytoskeletal organization was also well established by day 1, with only slight increases up to day 9. Sarcomeric α-actinin organization developed more slowly, reaching its maximal value at day 6. Since nuclear morphology reflects cytoskeletal tension (Bray et al., 2010; Lee et al., 2015), we quantified the nuclear aspect ratio to further assess cytoskeletal stresses. Compared with unpatterned hiPSC-CMs, cell pair hiPSC-CMs exhibited elongated nuclei (higher aspect ratio), with greater alignment between nuclear and cellular long axes (Figures S3C and S3D). Together, these results suggest that shape-controlled culture of hiPSC-CMs induced and standardized cytoskeletal and cell-cell junction formation.

Impaired assembly of cell-cell junctions in *ACM* hiPSC-CM cell pairs

Truncating and missense variants in *PKP2* are the most frequent cause of ACM (Gerull et al., 2004). The consequences of *PKP2* pathogenic variants on spatiotemporal assembly of cell-cell junctions have yet to be shown in human cardiomyocytes or hiPSC-CMs. To address this question, we used the cell pair platform to compare cell-cell junction assembly in *PKP2* mutant and isogenic control hiPSC-CMs. Using CRISPR-Cas9-mediated genome editing (Wang et al., 2017), we created two iPSC lines (clones C93 and C98) heterozygous for $PKP2^{R413X}$, an established pathogenic truncating variant (Figures S4A, S4B, and Table S1) (Syrris et al., 2006). Sanger sequencing did not show off-target mutagenesis at the three top predicted potentials, and digital karyotyping using Nanostring technology did not detect significant copy number variations compared with the parental line (Figures S4C and S4D). Genome-edited and control iPSCs expressed pluripotency markers (Figure S4E), and both genotypes exhibited comparable hiPSC-CM differentiation efficiencies (Figure S4F). Here these $PKP2^{R413X/+}$ iPSCs are referred to as *ACM*; *ACM-C98* was used for the main experiments, and key results were validated using *ACM-C93* as indicated.

ACM hiPSC-CMs cell pairs exhibited defective cell-cell junction assembly compared with isogenic controls. Isogenic control hiPSC-CM cell pairs consistently localized N-cadherin, plakoglobin, and PKP2 to cell-cell junctions by day 6, and CX43 by day 9 (Figure 1). In contrast, *ACM* cell pairs showed diffuse cytoplasmic distribution of N-cadherin until day 9 (Figures 2A and 2B), indicating delayed formation of adherens junctions. Furthermore, *ACM* cell pairs failed to properly localize plakoglobin, PKP2, and CX43 to cell-cell junctions during the 9-day culture period (Figures 2C–2H), indicating failure of desmosome and gap junction assembly. Plakoglobin stained diffusely throughout the cytoplasm, and PKP2 and CX43 showed aberrant peri-nuclear immunoreactivity. Quantitative analysis of protein localization over 9 days confirmed abnormal localization of plakoglobin (days 6 and 9), PKP2 (days 3, 6, and 9), and CX43 (days 6 and 9) (Figures 2D, 2F, and 2H).

We then investigated the cytoskeletal organization in *ACM* cell pairs. Compared with isogenic controls, *ACM* cell pairs exhibited reduced F-actin organization and sarcomeric α-actinin formation (Figure 3). Quantitative analysis confirmed reduced F-actin alignment during the early cell pair assembly (days 1 and 3; Figure 3E) and subsequently reduced sarcomere organization (Z-disc presence at days 3, 6, and 9) (Figures 3F and 3G). Increased sarcomere organization in wild-type (WT) cell pairs on days 6 and 9 was associated with enhanced formation of cell-cell junctions, as indicated by junctional localization of intercalated disc proteins in WT compared with *ACM* cell pairs (Figure 2).

SB216763 induces myofibrillogenesis and restores junctional integrity in *ACM* hiPSC-CM cell pairs

Reduced Wnt/β-catenin signaling is hypothesized to contribute to ACM pathogenesis (Austin et al., 2019; Garcia-Gras et al., 2006). SB216763, a small molecule GSK-3 inhibitor and Wnt activator, restores junctional protein localization and normalizes electrical activity in animal and human cell models of ACM (Asimaki et al., 2014; Chelko et al., 2016). Here we asked if modulating the Wnt/β-catenin pathway via SB216763 would restore cytoskeletal organization and cell junction integrity in the hiPSC-CM cell pair model of ACM (Figures 4 and 5).

The effect of SB216763 on *ACM* and control cell pairs is shown in representative images and corresponding quantitative analysis at day 6 (Figures 4A–4F) and day 9 (Figures 4G–4L). At both time points, ACM + vehicle (veh) had fewer vertically oriented α-actinin fibrils (Z-disc presence) than WT + veh, and this deficiency was rescued by SB216763 (Figure 4). While SB216763 enhanced cytoskeletal organization of *ACM* cell pairs, it had the opposite effect in WT cell pairs: WT + SB216763 (SB) cell pairs had significantly lower actin alignment (F-actin OOP) compared with WT + veh cell pairs (Figures 4E and 4K).

Next, we examined the effects of SB on cell-cell junction formation. N-cadherin localization to cell-cell junctions in *ACM* cell pairs was not significantly affected by SB. Both ACM + veh and ACM + SB exhibited delayed N-cadherin junctional localization with cytoplasmic distribution on day 6 (Figures S5A and S5B) and junctional localization by day 9 (Figures 5A–5C). In contrast, SB treatment made plakoglobin in *ACM* comparable with WT by day 9 (Figures 5D and 5E). However, plakoglobin localization remained abnormal on day 6 (Figures S5C and S5D), suggesting partial rescue

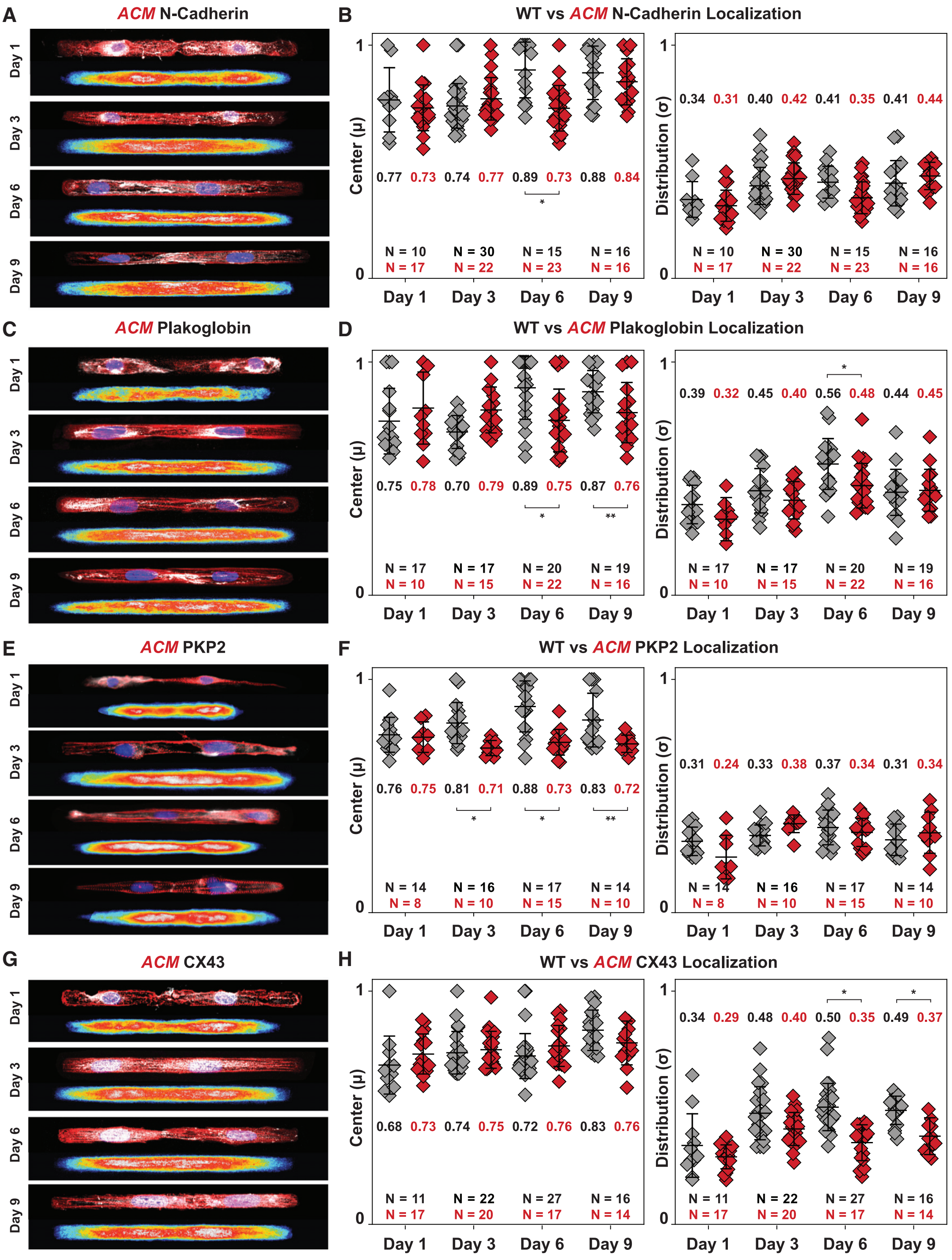

Figure 2. Compromised spatiotemporal assembly of cell-cell junctions in *ACM* hiPSC-CMs
(A–H) See also Figure S4. Representative immunostained images and averaged heatmaps of *ACM* hiPSC-CM cell pairs stained for nuclei (blue), α-actinin (red), and cell junction proteins (white). Quantified centers and distributions of intercalated disc protein localization in isogenic WT control and *ACM* hiPSC-CM cell pairs for cell junction proteins are shown on the right. Mean ± SD. two-way ANOVA followed by Tukey's multiple comparisons test: $^{*}p < 0.05$, $^{**}p < 0.01$. Cell pairs were aggregated from three to five independent differentiations.

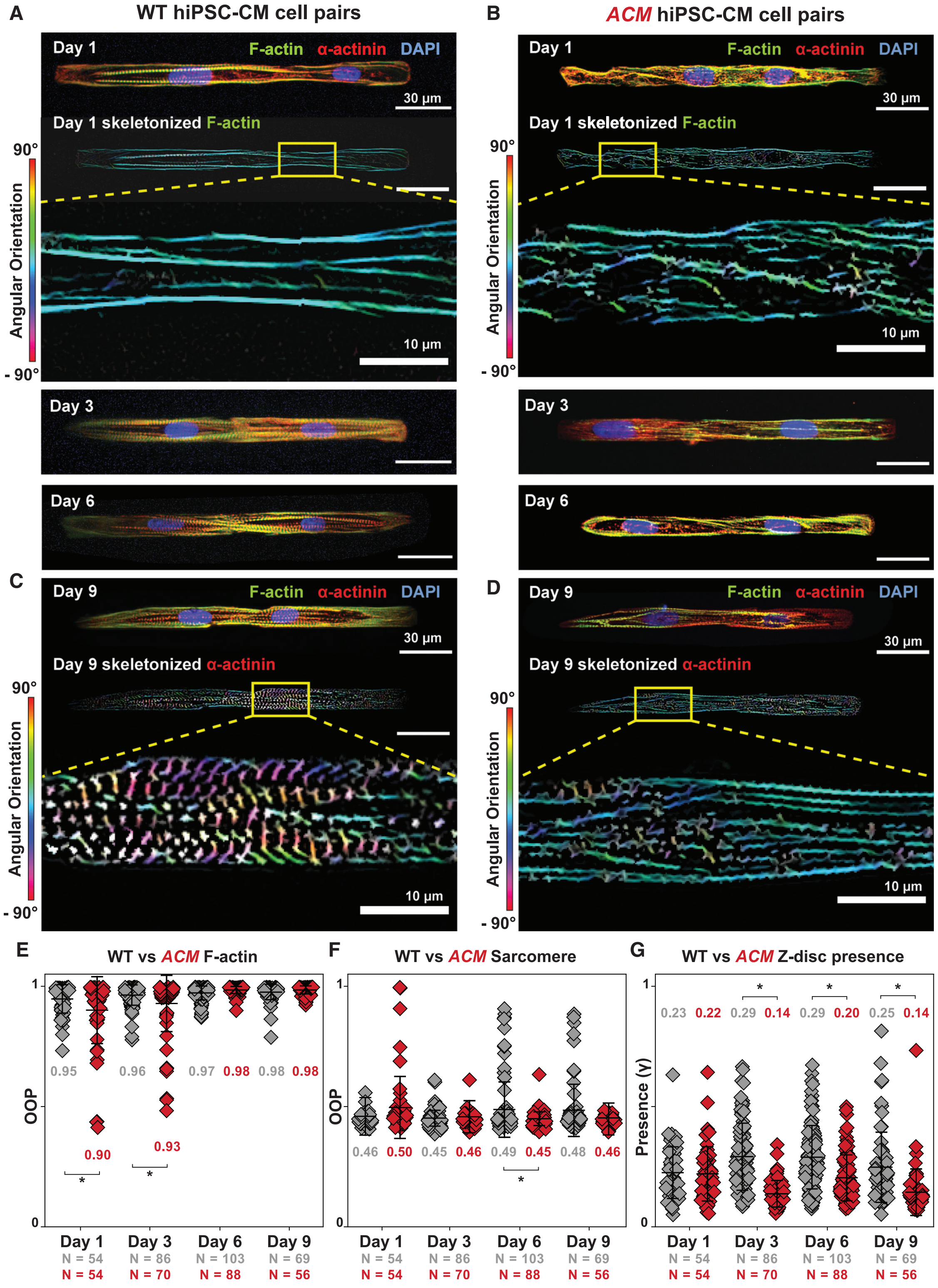

Figure 3. Compromised spatiotemporal assembly of the cytoskeletal filaments in *ACM* hiPSC-CMs

(A and B) Representative images of isogenic WT (A) and ACM (B) hiPSC-CM cell pairs stained for F-actin (green), sarcomeric α-actinin (red), and DAPI (blue). Day 1 skeletonized F-actin images, pseudo-colored based on fibril angular orientation, are shown below with boxed region magnified in the inset.

(legend continued on next page)

with continued abnormalities in desmosome assembly kinetics. PKP2 localization was also not affected by SB. Both ACM + veh and ACM + SB exhibited mislocalization of PKP2 on days 6 and 9 (Figures S5E, S5F, and 5G–5I). On day 9, CX43 exhibited junctional localization in WT + veh, whereas it was primarily perinuclear in ACM + veh cell pairs. After SB treatment, CX43 localization in ACM + SB became comparable with WT + veh. (Figures 5J–5L). We further validated the effects of SB on *PKP2* mutant cell-cell junctions by repeating the experiments on a second independent *ACM* clone, *ACM-C93*. Baseline defects and responses to SB were comparable between clones (Figure S6). Together these data indicate that SB ameliorates junctional complex assembly defects observed in *ACM* hiPSC-CM cell pairs.

Whereas SB ameliorated abnormal junctional protein localization in *ACM* cell pairs, it disrupted junctional localization in WT hiPSC-CM cell pairs. WT + SB showed reduced junctional localization of N-cadherin first on day 6 (Figures S5A and S5B). By day 9, N-cadherin, plakoglobin, and CX43 became mislocalized to the peri-nuclear region, indicated by lower values for centers and reduced distribution widths (Figures 5C, 5F, and 5L). Together with its effect on F-actin alignment (Figure 4), these data indicate that SB impaired WT cell pair cytoskeletal and junctional assembly.

To gain further insight into the molecular mechanisms underlying the observed structural changes, we used subcellular fractionation and capillary western blotting to quantify changes in protein levels or localization. Because of the amount of protein needed for these studies, monolayer cultures were used. The PKP2 protein level was significantly reduced in ACM hiPSC-CMs (Figure 6A), indicating that the R413X allele impairs protein expression. If translated, $PKP2^{R413X}$ would produce a 45-kDa peptide. We did not observe a truncated PKP2 protein (Figures 6A and 6B), suggesting that the $PKP2^{R413X}$ protein is not synthesized or is unstable. Quantification of *PKP2* mRNA demonstrated reduced transcript levels in $PKP2^{R413X/+}$ cells, suggesting the mutant allele undergoes nonsense-mediated RNA decay. Together, these data suggest that phenotypic defects in $PKP2^{R413X/+}$ hiPSC-CMs are caused by a haploinsufficiency of PKP2. SB did not alter PKP2 transcript or protein levels, indicating that it affects downstream processes rather than expression of the mutant allele.

Next, we examined the level of junctional proteins (Figures 6C–6F). N-cadherin expression in either cytosolic or membrane fractions was not significantly altered by *PKP2* genotype or SB treatment (Figures 6D and 6F left). In veh-treated cells, plakoglobin was significantly decreased in the membrane fraction and increased in the cytosolic fraction of ACM compared with WT (Figures 6D and 6F middle), consistent with impaired desmosome assembly and plakoglobin membrane localization. SB did not increase plakoglobin in the membrane fraction in ACM monolayers. This result contrasts with improved junctional plakoglobin localization observed in ACM cell pairs by immunostaining (Figure 5F), perhaps reflecting differences in cell junction assembly in cell pairs and monolayers. CX43 was significantly elevated in the cytosolic fraction of ACM cells (Figure 6D right), suggesting impaired trafficking or membrane localization. Correspondingly, CX43 levels were lower in the ACM membrane fraction (Figure 6F right), although this difference was not statistically significant because of within-group variation. While SB improved CX43 localization at the junction of ACM cell pairs, we did not detect a significant effect on membrane or cytosolic fractions of monolayers, again suggesting differences in cell junction assembly between cell pairs and monolayers.

SB enhances canonical Wnt signaling by stabilizing cytoplasmic or nuclear β-catenin, the effector of Wnt signaling (Zhurinsky et al., 2000). Nuclear β-catenin was significantly decreased in *ACM* hiPSC-CMs compared with isogenic controls, consistent with reduced canonical Wnt signaling (Figures 6G and 6H). Cytoplasmic β-catenin was elevated in *ACM* hiPSC-CMs compared with WT, suggesting impaired nuclear localization. SB treatment of *ACM* hiPSC-CMs significantly reduced cytosolic and significantly increased nuclear β-catenin, confirming activation and normalization of Wnt/β-catenin signaling. Increased nuclear β-catenin was also observed in WT hiPSC-CMs. Consistent with prior studies (Asimaki et al., 2014; Chelko et al., 2016), these data indicate that SB increases canonical Wnt signaling in ACM hiPSC-CMs, which likely contributes to improved cell-cell junction assembly in ACM cell pairs. We also note increased canonical Wnt signaling in WT hiPSC-CMs, which may impair their maturation and cell-cell junction assembly.

SB rescued calcium wave velocity in *ACM* hiPSC-CM tissues

A hallmark of ACM, particularly in its early concealed phase, is arrhythmia out of proportion to clinically apparent structural remodeling (Austin et al., 2019). To model arrhythmogenesis in ACM, we engineered tissues using isogenic WT and *ACM* hiPSC-CMs to quantify changes to cardiac propagation *in vitro*. We engineered biomimetic tissues with micro-molded

(C and D) Representative images of day 9 WT (C) and *ACM* (D) hiPSC-CM cell pairs with skeletonized α-actinin images below. Boxed regions are magnified in the inset.

(E–G) Quantified values of cytoskeletal organization over 9 days in culture. ACM cell pairs showed slower F-actin alignment (E), comparable α-actinin alignment (F), and reduced Z-disc presence (G). Mean ± SD. Two-way ANOVA followed by Tukey's multiple comparison test: $^{*}p < 0.05$. Cell pairs were aggregated from four to five independent differentiations.

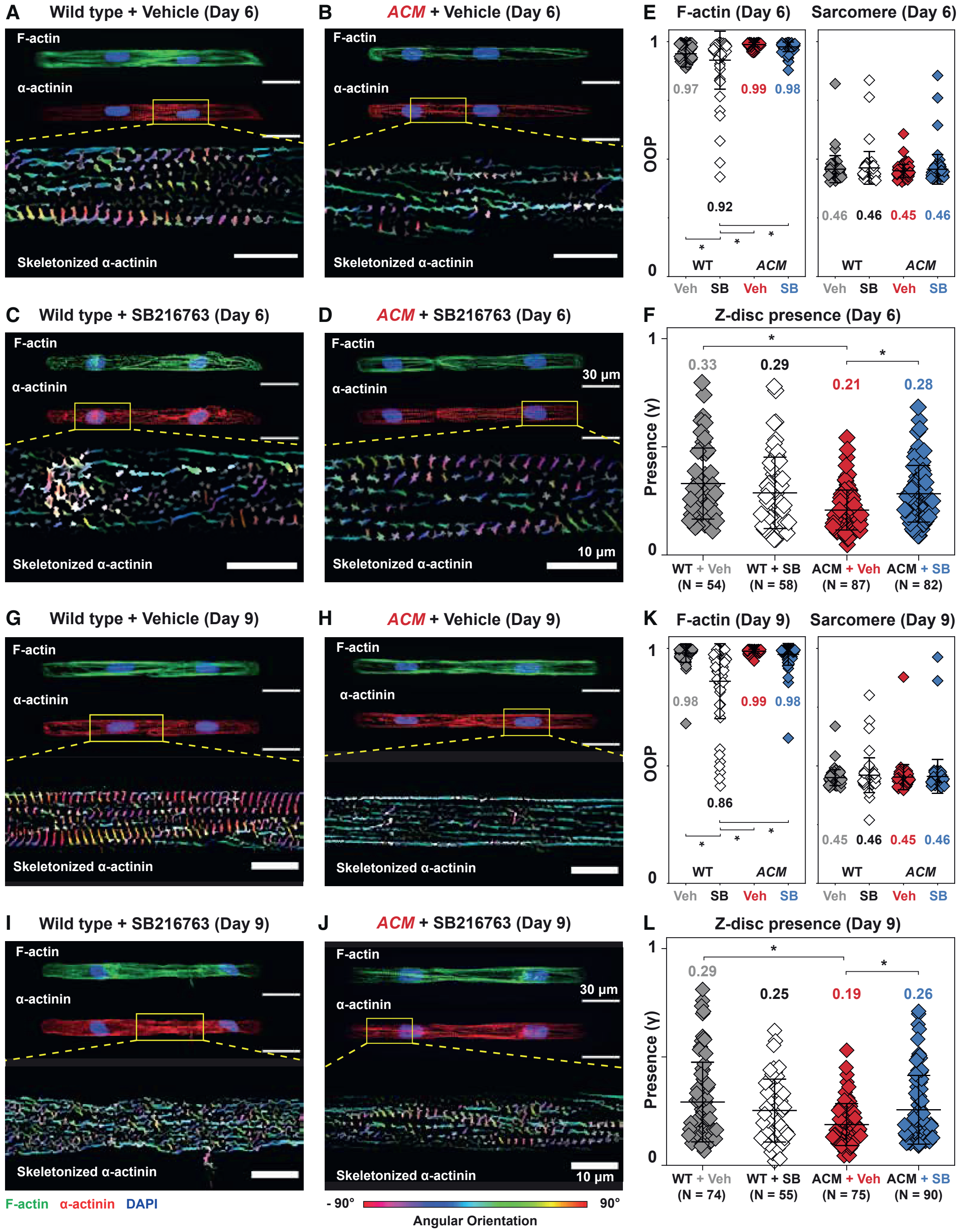

Figure 4. SB restores cytoskeletal integrity in day 6 and 9 *ACM* hiPSC-CMs

(A–F) Day 6 WT and *ACM* hiPSC-CM cell pairs treated with DMSO (veh) or SB. Representative images of cell pairs stained for F-actin (green) and α-actinin (red), with corresponding skeletonized fibrils pseudo-colored based on angular orientation.

(E) Quantified values for cytoskeletal organization (F-actin and sarcomere OOP) were unaffected, but modest improvement to sarcomere formation (z-disc presence) was observed in ACM + SB (F).

(legend continued on next page)

ridges to promote hiPSC-CM alignment (Lee et al., 2022). An elongated, narrow neck in the tissues (Figures 7A and 7B) was designed to establish a favorable electrical source-sink relationship and enhance Ca^{2+} wave propagation into the neck. *ACM* or isogenic WT control hiPSC-CMs seeded on these substrates were grown to confluence for 6 to 9 days to allow for cell junction formation (Figure S7). We electrically paced the tissues by point stimulation at the larger base and recorded Ca^{2+} wave propagations through the neck using a Ca^{2+}-sensitive fluorescent dye, X-Rhod-1. Ca^{2+} wave velocities of WT hiPSC-CM tissues averaged 15.4 ± 3.0 cm/s at day 6 and 21.4 ± 5.6 cm/s at day 9 (Figures 7C and 7D; Movie S1). These velocities are substantially higher than similar monolayer tissues without geometric constraints (Park et al., 2019) and comparable with velocities recorded from three-dimensional engineered heart tissue (Chang et al., 2022; Zhang and Pu, 2018). In comparison, *ACM* tissues exhibited significantly slower average Ca^{2+} wave propagation velocities at both day 6 (7 ± 2.9 cm/s) and day 9 (10.7 ± 3.3 cm/s) (Figure 7D; Movie S1).

We investigated the effect of SB on Ca^{2+} wave propagation in control and *ACM* tissues. SB did not significantly affect the propagation velocity of control tissues at either day 6 or 9 (Figure 7D). In contrast, SB dramatically increased Ca^{2+} wave velocities of *ACM* tissues at day 9 to 18.1 ± 4.8 cm/s, a velocity comparable with controls (Figure 7D). This increase in propagation velocity of *ACM* tissues was not observed on day 6, consistent with our observations in cell pairs that SB significantly changed cell junction localization on day 9, but not day 6.

We analyzed the effects of SB on cell junction protein localization in engineered tissues. Immunostaining with cell junction markers demonstrated that SB improved plakoglobin and CX43 localization in *ACM* tissues from a diffuse cytoplasmic distribution toward a more distinct localization around cell borders (Figures S7D and S7F), suggesting that it has similar effects on cell junction protein localization in *ACM* tissues and cell pairs. Together, these results indicate that SB remodeled cell-cell junctions of *ACM* hiPSC-CMs and markedly improved Ca^{2+} propagation velocity of *ACM* tissues.

DISCUSSION

In this study, we evaluated the effects of an established *ACM* pathogenic variant (*PKP2*$^{R413X/+}$) on cell-cell junctions in engineered cardiac tissues. *PKP2*$^{R413X/+}$ cells expressed decreased PKP2 protein, which impaired sarcomere and cytoskeletal assembly, consistent with prior studies (Inoue et al., 2022; Zhang et al., 2021). Our findings further suggest that *PKP2*$^{R413X/+}$ impairs formation of cell-cell junctions in human iPSC-CM models and that these changes are mitigated by SB's activation of Wnt/β-catenin signaling.

We leveraged the accessibility of hiPSC-CMs to monitor the spatiotemporal assembly of human cardiomyocyte junctions and their derangement by a pathogenic ACM variant. Cells organize their cytoskeletal architecture in response to geometric cues in the ECM (Grosberg et al., 2011). As the cellular architecture develops and cell-cell interactions dominate cell-ECM interactions (McCain et al., 2012a), the directionality of stresses within the cell changes, affecting nuclear morphologies (Bray et al., 2010; Lee et al., 2015). Cytoskeletal alignment also mediates the formation and localization of adherens junctions between cells, thus regulating desmosome and gap junction localization (Peters et al., 1994; Saffitz and Kléber, 2004; Vite and Radice, 2014). In healthy hiPSC-CM cell pairs, ECM-induced actin alignment occurred within 24 h, nuclear elongation, and adherens junction (N-cadherin) localization at cell junctions by day 6, and gap junction (CX43) localization by day 9. These processes were all disrupted in *PKP2*$^{R413X/+}$ hiPSC-CMs, starting with delayed actin alignment, then reduced sarcomere and cell junction formation, and ultimately mislocalization of plakoglobin and CX43. The alterations to junctions observed at day 9 in ACM cell pairs, characterized by reduced plakoglobin and CX43 along with preserved N-cadherin, mirror steady-state observations in human ACM hearts (Asimaki et al., 2009; Austin et al., 2019).

ACM sequence variants have been associated with reduced Wnt/β-catenin signaling (Garcia-Gras et al., 2006; Zhurinsky et al., 2000). Therapeutic intervention through increased Wnt/β-catenin signaling, via inhibition of GSK-3, ameliorated ACM phenotypes in zebrafish, rat cultured cardiomyocyte, and mouse models (Asimaki et al., 2014; Chelko et al., 2016). Likewise, we observed reduced Wnt/β-catenin signaling in *PKP2*$^{R413X/+}$ mutant human iPSC-CMs, and this was ameliorated with SB treatment. Specifically, SB induced myofibrillogenesis, restored mechanical and electrical coupling in *PKP2* mutant cell pairs, and improved Ca^{2+} wave velocity in *PKP2* mutant tissues. Our findings suggest that aberrant myofibrillogenesis and reduced Wnt/β-catenin signaling contribute to maladaptive cell junction formation and abnormalities in excitation-contraction coupling.

(G–L) Day 9 WT and *ACM* hiPSC-CM cell pairs treated with veh or SB. Similar trends are observed with more pronounced significant improvements to sarcomere formation in ACM + SB were observed (L). Whereas SB improved cytoskeletal integrity in ACM cell pairs, it reduced these metrics (E, F, K, L) in WT cell pairs. Mean ± SD. Two-way ANOVA followed by Tukey's multiple comparisons test: *$p < 0.05$. Cell pairs were aggregated from three to five independent differentiations.

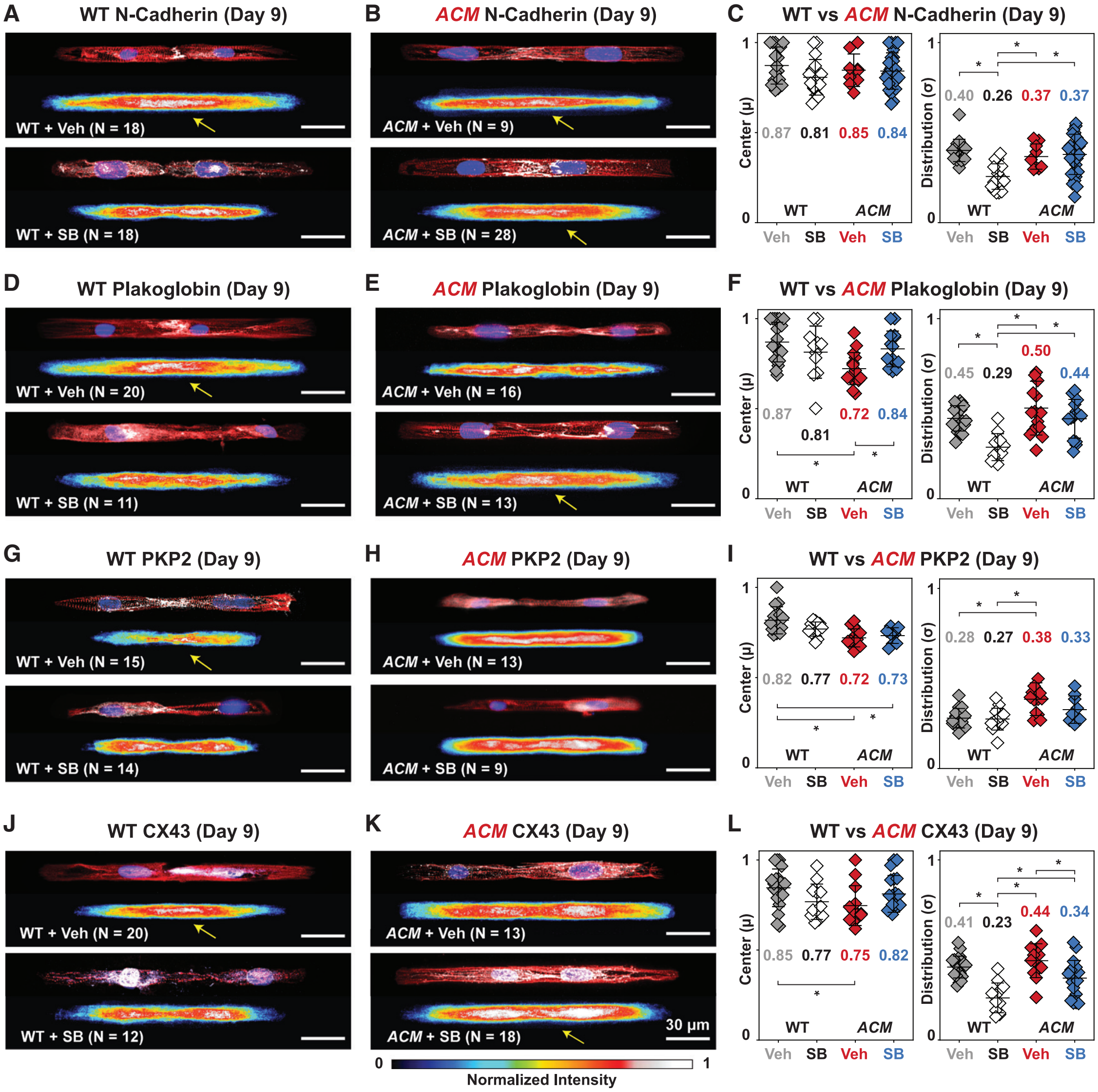

Figure 5. SB normalizes cell junction protein distribution in day 9 *ACM* hiPSC-CMs

See also Figures S5 and S6. WT or ACM hiPSC-CM cell pairs treated with DMSO (veh) or SB and stained at day 9 for nuclei, α-actinin, and cell junction proteins.

(A, B, D, E, G, H, J, and K). Representative images and corresponding averaged heatmaps. Yellow arrows indicate junctional localization.

(C, F, I, and L) Quantitative analysis of cell junction protein localization center (left) and distribution (right) in WT or *ACM* hiPSC-CMs at day 9. Significantly impaired plakoglobin and CX43 localization in ACM + veh were restored by SB. N-cadherin and PKP2 were unaffected by SB in *ACM* hiPSC-CMs. Mean ± SD. Two-way ANOVA followed by Tukey's multiple comparisons test: $*p < 0.05$.

Arrhythmias in ACM are likely influenced by multiple factors, including cell death, fibrofatty myocardial replacement (Austin et al., 2019), aberrant intracellular Ca^{2+} homeostasis (Cerrone et al., 2017), and altered cardiac sodium currents (Khudiakov et al., 2020; Sato et al., 2009). Our data demonstrate that ACM strongly reduces Ca^{2+} wave propagation, which would increase myocardial vulnerability to re-entrant arrhythmias. Reduced Ca^{2+} wave propagation was likely caused by impaired formation of cell-cell junctions and mis-localization of CX43. Previous studies have directly correlated CX43 immunofluorescence to cell-to-cell conductance across cardiomyocytes (McCain et al., 2012b). Treating *PKP2*$^{R413X/+}$ hiPSC-CMs with SB improved CX43 localization and restored Ca^{2+} wave velocities in *PKP2*$^{R413X/+}$ tissues, changes that would be anticipated to reduce the myocardial substrate's vulnerability to arrhythmia.

In contrast, we observed that GSK-3 inhibition and hyperactivation of Wnt/β-catenin via SB impaired the

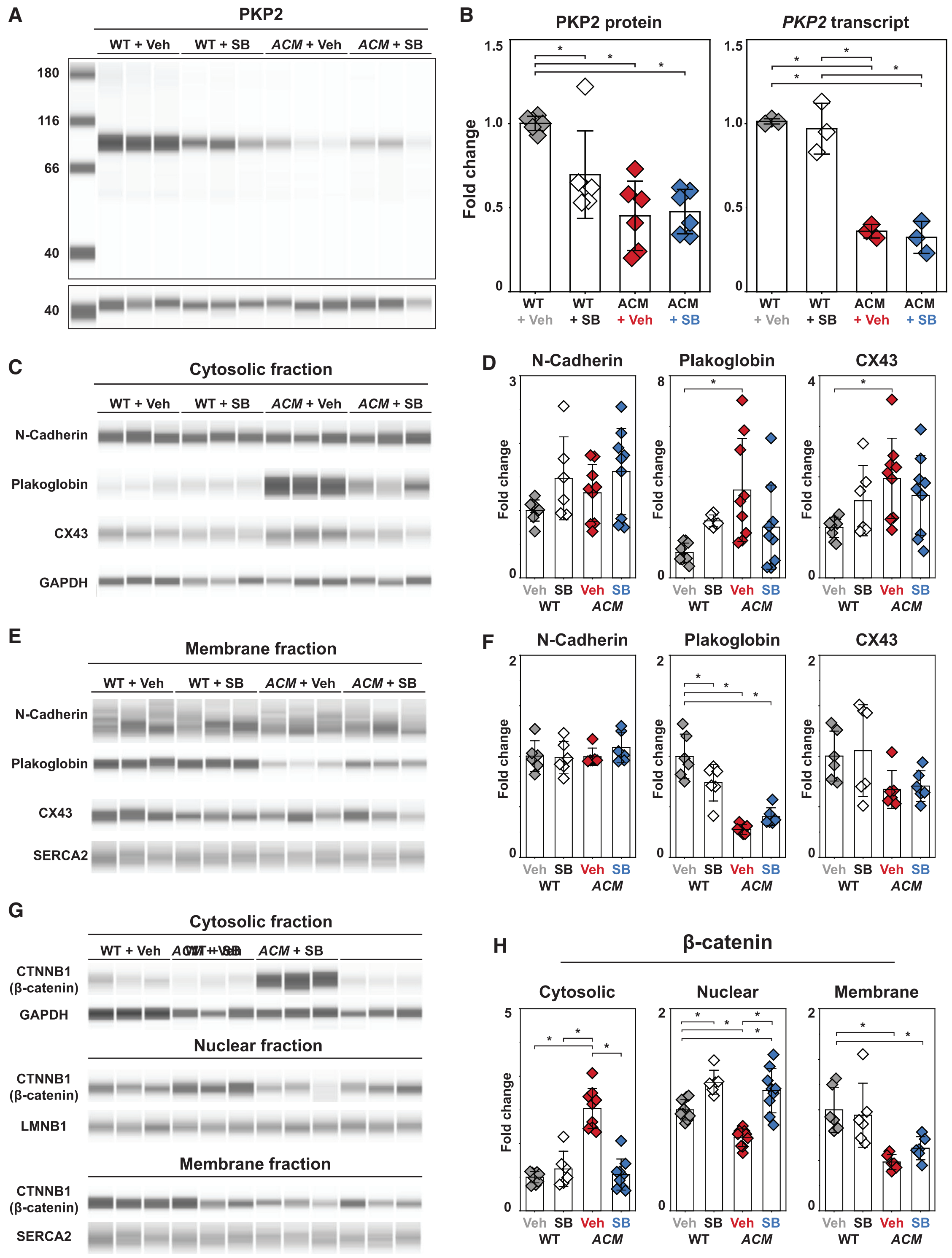

Figure 6. Abundance of proteins in cytoplasmic, nuclear, and membrane fractions of day 9 hiPSC-CMs

(A and B) See also Figure S7. Capillary western analysis of PKP2 and GAPDH. Relative changes to PKP2 protein and transcript levels with SB treatment in WT and ACM hiPSC-CMs. Transcript levels were measured by qRT-PCR and normalized to *GAPDH*.

(C–F) Capillary western analysis of cell junction proteins in cytosolic or membrane fractions. Relative levels of cell junction proteins were normalized to GAPDH (cytosolic fraction) or SERCA2 (membrane fraction) and expressed relative to WT + veh.

(legend continued on next page)

structural maturation of healthy hiPSC-CM cell pairs. Hyperactivation of Wnt/β-catenin initially disrupted cytoskeletal organization and mechanical coupling, followed by mislocalization of gap junctions in WT hiPSC-CMs. Regulation of Wnt/β-catenin signaling is critical for the differentiation of cardiac myocytes, and activation of this pathway in differentiated WT hiPSC-CMs promoted de-differentiation and proliferation (Buikema et al., 2020). However, in engineered tissues, we did not observe a deleterious effect of SB on cytoskeletal organization and cell coupling. This might suggest that cues in assembled tissues stabilize cell junctions and antagonize the de-differentiation effect observed in isolated cells (Buikema et al., 2020) or cell pairs. Consistent with this interpretation, SB did not adversely affect WT zebrafish or mice (Asimaki et al., 2014; Chelko et al., 2016). However, the deleterious effects that we observed on WT hiPSC-CMs raise concerns about cardiac effects of long-term exposure to SB. These add to existing concerns about oncogenic risks (Chelko et al., 2019; Zhan et al., 2017) and indicate that further studies of this class of compounds are required to understand their therapeutic mechanisms and potential adverse consequences in mature, *de facto* human cardiomyocytes. It is likely that other consequences of GSK-3 inhibition by SB also contribute. GSK-3 plays an integral role in cytoskeletal remodeling, and its perturbation modulated focal adhesion dynamics (Kobayashi et al., 2006), and actin and microtubule organization (Hajka et al., 2021).

In summary, our results demonstrate that $PKP2^{R413X/+}$ reduces Wnt/β-catenin signaling and perturbs cytoskeletal organization in human iPSC-CMs, leading to abnormal cell-cell junctions and impaired Ca^{2+} wave propagation. Furthermore, SB activated Wnt/β-catenin signaling, normalized cell-cell junctions, and rescued Ca^{2+} wave propagation in human $PKP2^{R413X/+}$ hiPSC-CM tissues, independent of changes to PKP2. These findings demonstrate our capacity to correlate structure-function relationships across spatial scales from genetic disease-causing variant to cell pairs and tissues, and to leverage the accessibility of hiPSC-CMs to analyze cardiomyocyte junction assembly *in vitro*. Future studies are required to determine the extent to which our findings for $PKP2^{R413X/+}$ extend to other variants in *PKP2* and other ACM genes.

EXPERIMENTAL PROCEDURES

Detailed experimental procedures are provided in the supplemental experimental procedures.

Resource availability

Requests for resources and reagents will be fulfilled by the corresponding authors.

Corresponding author

Further information and requests for resources, reagents, and materials should be directed to and will be fulfilled by corresponding authors Kevin Kit Parker (kkparker@g.harvard.edu) and William T. Pu (william.pu@cardio.chboston.edu)

Data and code availability

Analysis code generated and used in this study are available at https://doi.org/10.5281/ZENODO.7120682.

hiPSC-CM differentiation, culture, and micropatterning

The reference cell line WTC-Cas9 was derived from WTC-11 hiPSCs by inserting a doxycycline-inducible SpCas9 transgene (WTC-Cas9). The pathogenic $PKP2^{R413X/+}$ (*PKP2 c.1237C>T*) variant was introduced by CRISPR-Cas9 genome editing (Wang et al., 2017). Oligo sequences used are provided in Table S1. Cardiomyocytes were differentiated from iPSCs using WNT modulation (Lian et al., 2013) (Figure S2A).

PDMS stamps, created by photolithography (array of 14:1 rectangles 211 μm × 15 μm) were used to microcontact print ECM (fibronectin, Geltrex, or 1:1 fibronectin:Geltrex) onto PDMS-coated glass coverslips Figure S1). PDMS stamps (25-mm wide ridges, 4-mm grooves, and 5-mm groove depth) were used to micromold gelatin on a glass coverslip within a laser-cut acrylic mold, by modification of previously published protocols (Lee et al., 2022).

Ca^{2+} optical mapping and propagation velocity calculation

Samples were loaded with 2 μM X-Rhod-1 AM (Invitrogen, X14210). Relative cytoplasmic Ca^{2+} was imaged using a modified tandem-lens macroscope as described previously (Park et al., 2019) under 1 Hz electrical point stimulation. Data were analyzed using MATLAB (MathWorks) and the MiCAM imaging software (Scimedia) (Lee et al., 2022).

Immunostaining and data analysis

Samples were immunostained and imaged on a spinning disk confocal microscope (Olympus IX83, Andor spinning disk). Images were analyzed to quantify OOP and cytoskeletal alignment using previously published methods (Pasqualini et al., 2015; Sheehy et al., 2014; Wang et al., 2014), with slight modifications. Image preprocessing was performed with ImageJ/FIJI using the tubeness and OrientationJ plugins. Preprocessed images were then analyzed using MATLAB scripts (Pasqualini et al., 2015).

Protein localization analysis and heatmaps were performed using automated ImageJ/FIJI and MATLAB image processing scripts (Kim, 2022). Acquired cell pairs expressed sarcomeric α-actinin, a cardiomyocyte marker, covered most of the patterned area, and

(G and H) β-Catenin in cytosolic, nuclear, and membrane fractions was measured by capillary western. Relative β-catenin was normalized LMNB1 (nuclear fraction), GAPDH (cytosolic fraction), or SERCA2 (membrane fraction) and expressed relative to WT + veh. Mean ± SD. Two-way ANOVA followed by Tukey's multiple comparisons test: $*p < 0.05$. Data represent three technical replicates from two to three independent differentiations.

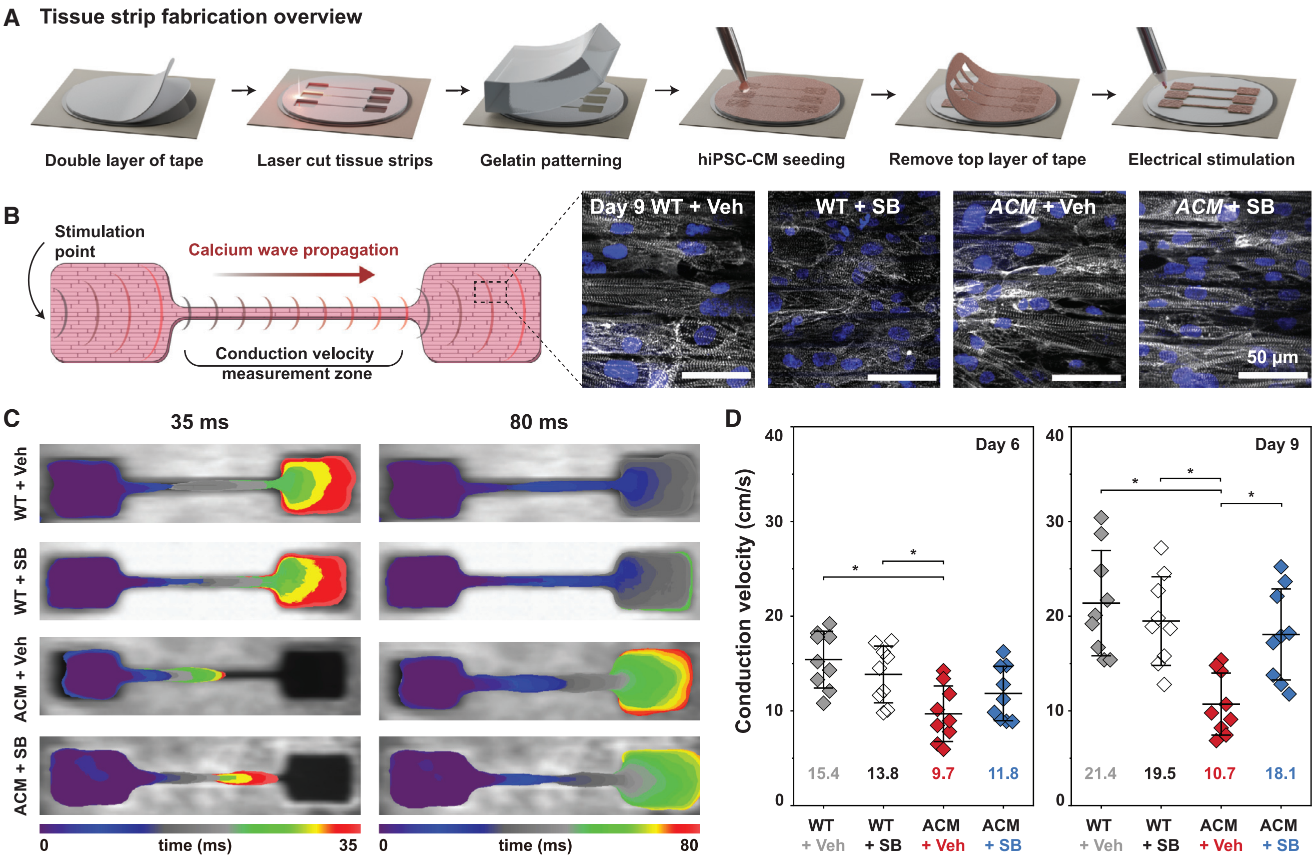

Figure 7. Ca^{2+} wave propagation velocity is reduced in *ACM* tissues and restored by SB
(A) Schematic illustration of engineered tissue fabrication.
(B) Design of tissue constructs for Ca^{2+} optical mapping experiments. Representative images of anisotropic tissues stained for sarcomeric α-actinin and nuclei demonstrate iPSC-CM alignment by micro-molded gelatin substrate.
(C) Calcium wavefront isochrone maps for day 9 WT + veh, WT + SB, ACM + veh, and ACM + SB tissues at two representative time points (35 and 80 ms).
(D) Ca^{2+} wave propagation velocities of WT and *ACM* tissues at day 6 and day 9, with and without SB treatment. Mean ± SD. Two-way ANOVA followed by Tukey's multiple comparisons test: $^{*}p < 0.05$.

contained two distinct nuclei. Cell junction markers were not used to decide to include or exclude a cell pair. Cells with nuclei <50 μm apart and horizontal cytoskeletal filaments spanning across both nuclei were excluded as likely binucleated cells.

Statistical analysis

Bar graphs indicate mean ± SD. Boxplots represent the interquartile range and median (box and center line) and 1.5 times the interquartile range (whiskers). Statistical analysis across calculated values were conducted using one-way ANOVA or two-way ANOVA followed by Tukey's honest significant difference test or test for multiple comparisons using OriginPro (ver 2023, OriginLab Corporation). Statistically significant p values ($p < 0.05$ and $p < 0.1$) are indicated within the graphs where appropriate.

SUPPLEMENTAL INFORMATION

Supplemental information can be found online at https://doi.org/10.1016/j.stemcr.2023.07.005.

AUTHOR CONTRIBUTIONS

S.L.K., M.A.T., K.Y.L., L.A.M., K.K.P., and W.T.P. designed research; S.L.K., M.A.T., and K.Y.L. performed research; S.C., J.F.Z., L.D.W., K.S., D.E.H., L.J.L., and X.L. contributed new reagents/analytic tools; S.L.K., M.A.T., and K.Y.L., analyzed data; and S.L.K., M.A.T., K.K.P., and W.T.P. wrote the paper.

ACKNOWLEDGMENTS

We thank A.G. Kleber for discussions and Michael Rosnach for illustrations. This work was funded by the Harvard Paulson School of Engineering and Applied Sciences, the Wyss Institute for Biologically Inspired Engineering, NHLBI and NCATS (UG3HL141798, UH3HL141798, R01HL167450, and T32HL007572), and the Kaplan Cardiology Fellowship at Boston Children's Hospital. This work was performed in part at the Harvard University Center for Nanoscale Systems (CNS), which is supported by the National Science Foundation (ECCS-2025158).

CONFLICT OF INTERESTS

The authors have no competing interests to disclose.

Received: January 12, 2023
Revised: July 21, 2023
Accepted: July 22, 2023
Published: August 17, 2023

REFERENCES

Aratyn-Schaus, Y., Pasqualini, F.S., Yuan, H., McCain, M.L., Ye, G.J.C., Sheehy, S.P., Campbell, P.H., and Parker, K.K. (2016). Coupling primary and stem cell-derived cardiomyocytes in an in vitro model of cardiac cell therapy. J. Cell Biol. *212*, 389–397. https://doi.org/10.1083/jcb.201508026.

Asimaki, A., Tandri, H., Huang, H., Halushka, M.K., Gautam, S., Basso, C., Thiene, G., Tsatsopoulou, A., Protonotarios, N., McKenna, W.J., et al. (2009). A New Diagnostic Test for Arrhythmogenic Right Ventricular Cardiomyopathy. N. Engl. J. Med. *360*, 1075–1084. https://doi.org/10.1056/NEJMoa0808138.

Asimaki, A., Kapoor, S., Plovie, E., Karin Arndt, A., Adams, E., Liu, Z., James, C.A., Judge, D.P., Calkins, H., Churko, J., et al. (2014). Identification of a New Modulator of the Intercalated Disc in a Zebrafish Model of Arrhythmogenic Cardiomyopathy HHS Public Access. Sci. Transl. Med. *6*, 240ra74. https://doi.org/10.1126/scitranslmed.3008008.

Austin, K.M., Trembley, M.A., Chandler, S.F., Sanders, S.P., Saffitz, J.E., Abrams, D.J., and Pu, W.T. (2019). Molecular mechanisms of arrhythmogenic cardiomyopathy. Nat. Rev. Cardiol. *16*, 519–537. https://doi.org/10.1038/s41569-019-0200-7.

Bhonsale, A., Groeneweg, J.A., James, C.A., Dooijes, D., Tichnell, C., Jongbloed, J.D.H., Murray, B., te Riele, A.S.J.M., van den Berg, M.P., Bikker, H., et al. (2015). Impact of genotype on clinical course in arrhythmogenic right ventricular dysplasia/cardiomyopathy-associated mutation carriers. Eur. Heart J. *36*, 847–855. https://doi.org/10.1093/eurheartj/ehu509.

Bliley, J.M., Vermeer, M.C.S.C., Duffy, R.M., Batalov, I., Kramer, D., Tashman, J.W., Shiwarski, D.J., Lee, A., Teplenin, A.S., Volkers, L., et al. (2021). Dynamic loading of human engineered heart tissue enhances contractile function and drives a desmosome-linked disease phenotype. Sci. Transl. Med. *13*, eabd1817. https://doi.org/10.1126/scitranslmed.abd1817.

Bray, M.-A.P., Adams, W.J., Geisse, N.A., Feinberg, A.W., Sheehy, S.P., and Parker, K.K. (2010). Nuclear morphology and deformation in engineered cardiac myocytes and tissues. Biomaterials *31*, 5143–5150. https://doi.org/10.1016/j.biomaterials.2010.03.028.

Buikema, J.W., Lee, S., Goodyer, W.R., Maas, R.G., Chirikian, O., Li, G., Miao, Y., Paige, S.L., Lee, D., Wu, H., et al. (2020). Wnt Activation and Reduced Cell-Cell Contact Synergistically Induce Massive Expansion of Functional Human iPSC-Derived Cardiomyocytes. Cell Stem Cell *27*, 50–63.e5. https://doi.org/10.1016/j.stem.2020.06.001.

Cerrone, M., Montnach, J., Lin, X., Zhao, Y.-T., Zhang, M., Agullo-Pascual, E., Leo-Macias, A., Alvarado, F.J., Dolgalev, I., Karathanos, T.V., et al. (2017). Plakophilin-2 is required for transcription of genes that control calcium cycling and cardiac rhythm. Nat. Commun. *8*, 106. https://doi.org/10.1038/s41467-017-00127-0.

Chang, H., Liu, Q., Zimmerman, J.F., Lee, K.Y., Jin, Q., Peters, M.M., Rosnach, M., Choi, S., Kim, S.L., Ardoña, H.A.M., et al. (2022). Recreating the heart's helical structure-function relationship with focused rotary jet spinning. Science *377*, 180–185. https://doi.org/10.1126/science.abl6395.

Chelko, S.P., Asimaki, A., Andersen, P., Bedja, D., Amat-Alarcon, N., DeMazumder, D., Jasti, R., MacRae, C.A., Leber, R., Kleber, A.G., et al. (2016). Central role for GSK3β in the pathogenesis of arrhythmogenic cardiomyopathy. JCI Insight *1*, 85923. https://doi.org/10.1172/jci.insight.85923.

Chelko, S.P., Asimaki, A., Lowenthal, J., Bueno-Beti, C., Bedja, D., Scalco, A., Amat-Alarcon, N., Andersen, P., Judge, D.P., Tung, L., and Saffitz, J.E. (2019). Therapeutic Modulation of the Immune Response in Arrhythmogenic Cardiomyopathy. Circulation *140*, 1491–1505. https://doi.org/10.1161/CIRCULATIONAHA.119.040676.

Corrado, D., Link, M.S., and Calkins, H. (2017). Arrhythmogenic Right Ventricular Cardiomyopathy. N. Engl. J. Med. *376*, 61–72. https://doi.org/10.1056/NEJMra1509267.

Cruz, F.M., Sanz-Rosa, D., Roche-Molina, M., García-Prieto, J., García-Ruiz, J.M., Pizarro, G., Jiménez-Borreguero, L.J., Torres, M., Bernad, A., Ruíz-Cabello, J., et al. (2015). Exercise triggers ARVC phenotype in mice expressing a disease-causing mutated version of human plakophilin-2. J. Am. Coll. Cardiol. *65*, 1438–1450. https://doi.org/10.1016/j.jacc.2015.01.045.

Garcia-Gras, E., Lombardi, R., Giocondo, M.J., Willerson, J.T., Schneider, M.D., Khoury, D.S., and Marian, A.J. (2006). Suppression of canonical Wnt/β-catenin signaling by nuclear plakoglobin recapitulates phenotype of arrhythmogenic right ventricular cardiomyopathy. J. Clin. Invest. *116*, 2012–2021. https://doi.org/10.1172/JCI27751.

Gerull, B., Heuser, A., Wichter, T., Paul, M., Basson, C.T., McDermott, D.A., Lerman, B.B., Markowitz, S.M., Ellinor, P.T., MacRae, C.A., et al. (2004). Mutations in the desmosomal protein plakophilin-2 are common in arrhythmogenic right ventricular cardiomyopathy. Nat. Genet. *36*, 1162–1164. https://doi.org/10.1038/ng1461.

Gessert, S., and Kühl, M. (2010). The multiple phases and faces of Wnt signaling during cardiac differentiation and development. Circ. Res. *107*, 186–199. https://doi.org/10.1161/CIRCRESAHA.110.221531.

Grosberg, A., Kuo, P.L., Guo, C.L., Geisse, N.A., Bray, M.A., Adams, W.J., Sheehy, S.P., and Parker, K.K. (2011). Self-organization of muscle cell structure and function. PLoS Comput. Biol. *7*, e1001088. https://doi.org/10.1371/journal.pcbi.1001088.

Hajka, D., Budziak, B., Pietras, Ł., Duda, P., McCubrey, J.A., and Gizak, A. (2021). GSK3 as a regulator of cytoskeleton architecture: Consequences for health and disease. Cells *10*. https://doi.org/10.3390/cells10082092.

Inoue, H., Nakamura, S., Higo, S., Shiba, M., Kohama, Y., Kondo, T., Kameda, S., Tabata, T., Okuno, S., Ikeda, Y., et al. (2022). Modeling reduced contractility and impaired desmosome assembly due to plakophilin-2 deficiency using isogenic iPS cell-derived

cardiomyocytes. Stem Cell Rep. *17*, 337–351. https://doi.org/10.1016/j.stemcr.2021.12.016.

Khudiakov, A., Zaytseva, A., Perepelina, K., Smolina, N., Pervunina, T., Vasichkina, E., Karpushev, A., Tomilin, A., Malashicheva, A., and Kostareva, A. (2020). Sodium current abnormalities and deregulation of Wnt/β-catenin signaling in iPSC-derived cardiomyocytes generated from patient with arrhythmogenic cardiomyopathy harboring compound genetic variants in plakophilin 2 gene. Biochim. Biophys. Acta, Mol. Basis Dis. *1866*, 165915. https://doi.org/10.1016/j.bbadis.2020.165915.

Kim, S. (2022). Cell Pair Heatmap Overlay and Intensity Profile Extraction/fitting. https://doi.org/10.5281/ZENODO.7120682.

Kim, C., Wong, J., Wen, J., Wang, S., Wang, C., Spiering, S., Kan, N.G., Forcales, S., Puri, P.L., Leone, T.C., et al. (2013). Studying arrhythmogenic right ventricular dysplasia with patient-specific iPSCs. Nature *494*, 105–110. https://doi.org/10.1038/nature11799.

Kirchner, F., Schuetz, A., Boldt, L.H., Martens, K., Dittmar, G., Haverkamp, W., Thierfelder, L., Heinemann, U., and Gerull, B. (2012). Molecular insights into arrhythmogenic right ventricular cardiomyopathy caused by plakophilin-2 missense mutations. Circ. Cardiovasc. Genet. *5*, 400–411. https://doi.org/10.1161/CIRCGENETICS.111.961854.

Kobayashi, T., Hino, S.i., Oue, N., Asahara, T., Zollo, M., Yasui, W., and Kikuchi, A. (2006). Glycogen Synthase Kinase 3 and h-prune Regulate Cell Migration by Modulating Focal Adhesions. Mol. Cell Biol. *26*, 898–911. https://doi.org/10.1128/mcb.26.3.898-911.2006.

Komiya, Y., and Habas, R. (2008). Wnt signal transduction pathways. Organogenesis *4*, 68–75.

Lee, H., Adams, W.J., Alford, P.W., McCain, M.L., Feinberg, A.W., Sheehy, S.P., Goss, J.A., and Parker, K.K. (2015). Cytoskeletal prestress regulates nuclear shape and stiffness in cardiac myocytes. Exp. Biol. Med. *240*, 1543–1554. https://doi.org/10.1177/1535370215583799.

Lee, K.Y., Park, S.J., Matthews, D.G., Kim, S.L., Marquez, C.A., Zimmerman, J.F., Ardoña, H.A.M., Kleber, A.G., Lauder, G.V., and Parker, K.K. (2022). An autonomously swimming biohybrid fish designed with human cardiac biophysics. Science *375*, 639–647. https://doi.org/10.1126/science.abh0474.

Lian, X., Zhang, J., Azarin, S.M., Zhu, K., Hazeltine, L.B., Bao, X., Hsiao, C., Kamp, T.J., and Palecek, S.P. (2013). Directed cardiomyocyte differentiation from human pluripotent stem cells by modulating Wnt/β-catenin signaling under fully defined conditions. Nat. Protoc. *8*, 162–175. https://doi.org/10.1038/nprot.2012.150.

Manring, H.R., Dorn, L.E., Ex-Willey, A., Accornero, F., and Ackermann, M.A. (2018). At the heart of inter- and intracellular signaling: the intercalated disc. Biophys. Rev. *10*, 961–971. https://doi.org/10.1007/s12551-018-0430-7.

Mazzanti, A., Ng, K., Faragli, A., Maragna, R., Chiodaroli, E., Orphanou, N., Monteforte, N., Memmi, M., Gambelli, P., Novelli, V., et al. (2016). Arrhythmogenic Right Ventricular Cardiomyopathy: Clinical Course and Predictors of Arrhythmic Risk. J. Am. Coll. Cardiol. *68*, 2540–2550. https://doi.org/10.1016/j.jacc.2016.09.951.

McCain, M.L., Lee, H., Aratyn-Schaus, Y., Kléber, A.G., and Parker, K.K. (2012a). Cooperative coupling of cell-matrix and cell-cell adhesions in cardiac muscle. Proc. Natl. Acad. Sci. USA *109*, 9881–9886. https://doi.org/10.1073/pnas.1203007109.

McCain, M.L., Desplantez, T., Geisse, N.A., Rothen-Rutishauser, B., Oberer, H., Parker, K.K., and Kleber, A.G. (2012b). Cell-to-cell coupling in engineered pairs of rat ventricular cardiomyocytes: relation between Cx43 immunofluorescence and intercellular electrical conductance. Am. J. Physiol. Heart Circ. Physiol. *302*, H443–H450. https://doi.org/10.1152/ajpheart.01218.2010.

Oxford, E.M., Musa, H., Maass, K., Coombs, W., Taffet, S.M., and Delmar, M. (2007). Connexin43 remodeling caused by inhibition of plakophilin-2 expression in cardiac cells. Circ. Res. *101*, 703–711. https://doi.org/10.1161/CIRCRESAHA.107.154252.

Padrón-Barthe, L., Villalba-Orero, M., Gómez-Salinero, J.M., Domínguez, F., Román, M., Larrasa-Alonso, J., Ortiz-Sánchez, P., Martínez, F., López-Olañeta, M., Bonzón-Kulichenko, E., et al. (2019). Severe Cardiac Dysfunction and Death Caused by Arrhythmogenic Right Ventricular Cardiomyopathy Type 5 Are Improved by Inhibition of Glycogen Synthase Kinase-3β. Circulation *140*, 1188–1204. https://doi.org/10.1161/CIRCULATIONAHA.119.040366.

Park, S.J., Zhang, D., Qi, Y., Li, Y., Lee, K.Y., Bezzerides, V.J., Yang, P., Xia, S., Kim, S.L., Liu, X., et al. (2019). Insights into the Pathogenesis of Catecholaminergic Polymorphic Ventricular Tachycardia from Engineered Human Heart Tissue. Circulation *140*, 390–404. https://doi.org/10.1161/CIRCULATIONAHA.119.039711.

Pasqualini, F.S., Sheehy, S.P., Agarwal, A., Aratyn-Schaus, Y., and Parker, K.K. (2015). Structural phenotyping of stem cell-derived cardiomyocytes. Stem Cell Rep. *4*, 340–347. https://doi.org/10.1016/j.stemcr.2015.01.020.

Peters, N.S., Severs, N.J., Rothery, S.M., Lincoln, C., Yacoub, M.H., and Green, C.R. (1994). Spatiotemporal relation between gap junctions and fascia adherens junctions during postnatal development of human ventricular myocardium. Circulation *90*, 713–725. https://doi.org/10.1161/01.CIR.90.2.713.

Saffitz, J.E., and Kléber, A.G. (2004). Effects of Mechanical Forces and Mediators of Hypertrophy on Remodeling of Gap Junctions in the Heart. Circ. Res. *94*, 585–591. https://doi.org/10.1161/01.RES.0000121575.34653.50.

Sato, P.Y., Musa, H., Coombs, W., Guerrero-Serna, G., Patiño, G.A., Taffet, S.M., Isom, L.L., and Delmar, M. (2009). Loss of plakophilin-2 expression leads to decreased sodium current and slower conduction velocity in cultured cardiac myocytes. Circ. Res. *105*, 523–526. https://doi.org/10.1161/CIRCRESAHA.109.201418.

Sheehy, S.P., Pasqualini, F., Grosberg, A., Park, S.J., Aratyn-Schaus, Y., and Parker, K.K. (2014). Quality metrics for stem cell-derived cardiac myocytes. Stem Cell Rep. *2*, 282–294. https://doi.org/10.1016/j.stemcr.2014.01.015.

Syrris, P., Ward, D., Asimaki, A., Sen-Chowdhry, S., Ebrahim, H.Y., Evans, A., Hitomi, N., Norman, M., Pantazis, A., Shaw, A.L., et al. (2006). Clinical expression of plakophilin-2 mutations in familial arrhythmogenic right ventricular cardiomyopathy. Circulation *113*, 356–364. https://doi.org/10.1161/CIRCULATIONAHA.105.561654.

Thomas, D., Choi, S., Alamana, C., Parker, K.K., and Wu, J.C. (2022). Cellular and Engineered Organoids for Cardiovascular

Models. Circ. Res. *130*, 1780–1802. https://doi.org/10.1161/CIRCRESAHA.122.320305.

Vite, A., and Radice, G.L. (2014). N-cadherin/catenin complex as a master regulator of intercalated disc function. Cell Commun. Adhes. *21*, 169–179. https://doi.org/10.3109/15419061.2014.908853.

Wang, G., McCain, M.L., Yang, L., He, A., Pasqualini, F.S., Agarwal, A., Yuan, H., Jiang, D., Zhang, D., Zangi, L., et al. (2014). Modeling the mitochondrial cardiomyopathy of Barth syndrome with induced pluripotent stem cell and heart-on-chip technologies. Nat. Med. *20*, 616–623. https://doi.org/10.1038/nm.3545.

Wang, G., Yang, L., Grishin, D., Rios, X., Ye, L.Y., Hu, Y., Li, K., Zhang, D., Church, G.M., and Pu, W.T. (2017). Efficient, footprint-free human iPSC genome editing by consolidation of Cas9/CRISPR and piggyBac technologies. Nat. Protoc. *12*, 88–103. https://doi.org/10.1038/nprot.2016.152.

Zhan, T., Rindtorff, N., and Boutros, M. (2017). Wnt signaling in cancer. Oncogene *36*, 1461–1473. https://doi.org/10.1038/onc.2016.304.

Zhang, D., and Pu, W.T. (2018). Exercising engineered heart muscle to maturity news-and-views. Nat. Rev. Cardiol. *15*, 383–384. https://doi.org/10.1038/s41569-018-0032-x.

Zhang, K., Cloonan, P.E., Sundaram, S., Liu, F., Das, S.L., Ewoldt, J.K., Bays, J.L., Tomp, S., Toepfer, C.N., Marsiglia, J.D.C., et al. (2021). Plakophilin-2 truncating variants impair cardiac contractility by disrupting sarcomere stability and organization. Sci. Adv. *7*, eabh3995. https://doi.org/10.1126/sciadv.abh3995.

Zhurinsky, J., Shtutman, M., and Ben-Ze'ev, A. (2000). Plakoglobin and β-catenin: Protein interactions, regulation and biological roles. J. Cell Sci. *113*, 3127–3139. https://doi.org/10.1242/jcs.113.18.3127.

Stem Cell Reports, Volume *18*

Supplemental Information

Spatiotemporal cell junction assembly in human iPSC-CM models of arrhythmogenic cardiomyopathy

Sean L. Kim, Michael A. Trembley, Keel Yong Lee, Suji Choi, Luke A. MacQueen, John F. Zimmerman, Lousanne H.C. de Wit, Kevin Shani, Douglas E. Henze, Daniel J. Drennan, Shaila A. Saifee, Li Jun Loh, Xujie Liu, Kevin Kit Parker, and William T. Pu

Supplemental Materials

Supplemental Figures

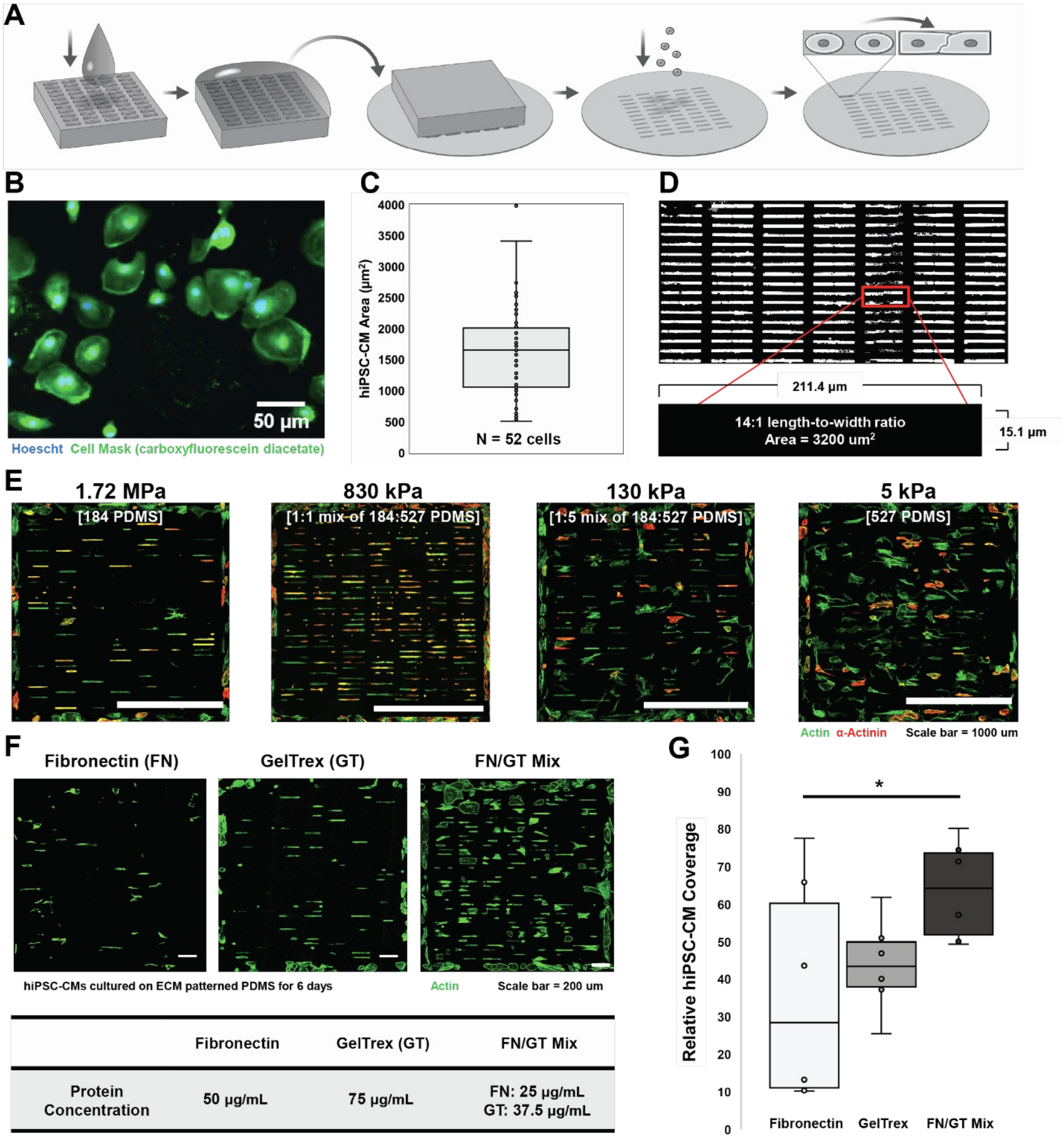

	Fibronectin	GelTrex (GT)	FN/GT Mix
Protein Concentration	50 μg/mL	75 μg/mL	FN: 25 μg/mL<br>GT: 37.5 μg/mL

Figure S1. Shape-controlled cell pair substrates for hiPSC-CMs. Related to Fig. 1. (A) Graphical overview of the process of microcontact printing to induce cell pair formation. ECM protein coated PDMS stamps are patterned onto PDMS coverslips containing and array of 14:1 aspect ratio rectangles. (B) Representative image of WT hiPSC-CMs grown on PDMS coated coverslips for 6 days post differentiation, stained with a cell mask and Hoescht for size analysis. (C) hiPSC-CM cell area analysis shows an average size of 1600 μm^2. N = 52 cells across 6 samples. (D) Representative image of a microcontact printed substrate stained with fibronectin with dimensions from CAD (computer-aided designs) images. (E) Representative coverage images using 4 different combinations of 184 PDMS and 527 PDMS with varying substrate stiffnesses. (F) Representative fluorescent images of hiPSC-CMs on microcontact printed substrates with various extracellular matrix proteins and their concentrations. (G) Cell coverage calculated as the total fluorescent actin area, relative to theoretical fibronectin printed values from the CAD design. N = 6 coverslips; > 100 field of views; 2 batches; *P < 0.05 by one-way ANOVA.

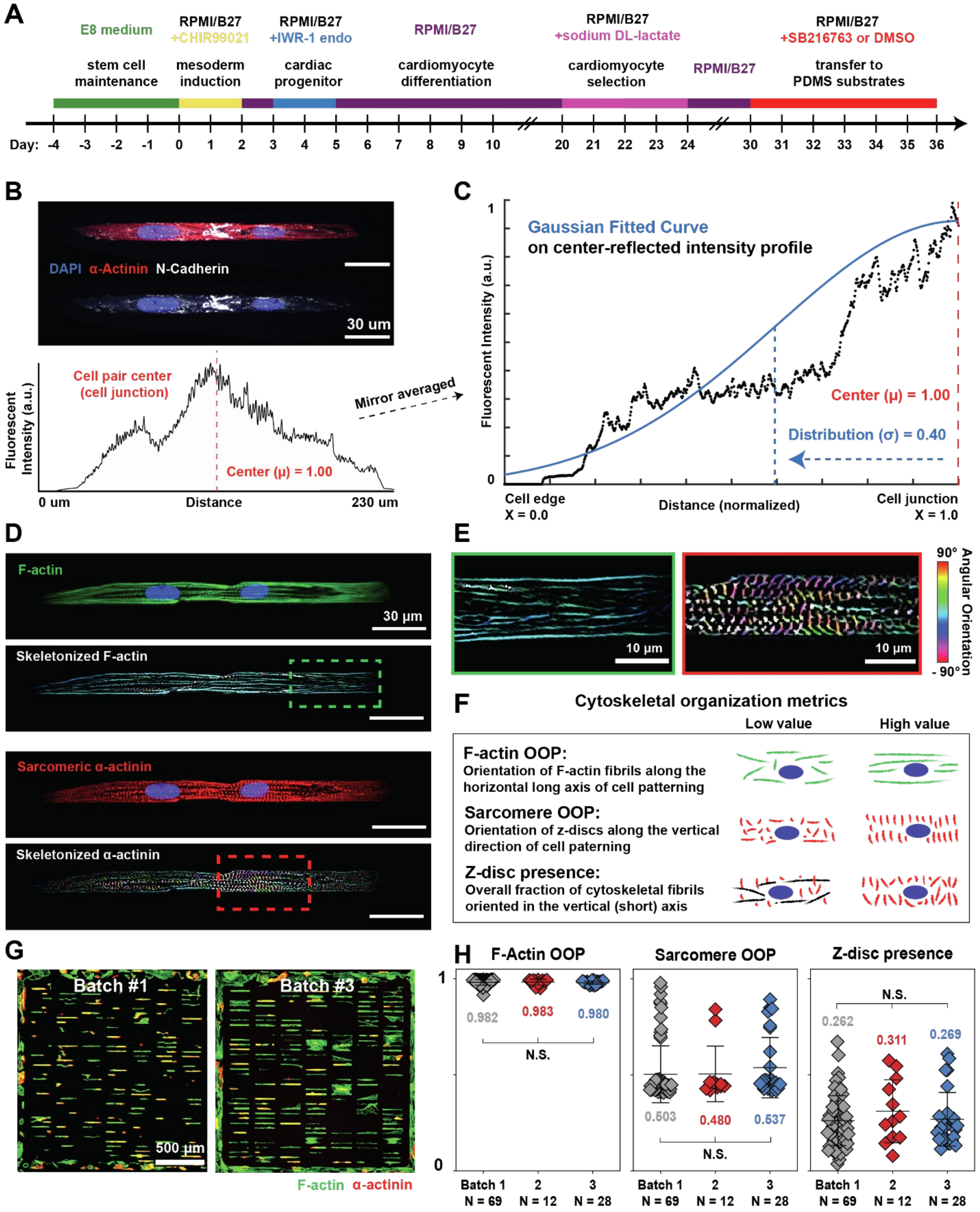

Figure S2. Quantitative metrics for assessing hiPSC-CM cell pair structural quality. Related to Fig. 1. (A) Schematic representation of the iPSC to iPSC-CM differentiation timeline and cell culture conditions. (B) Representative image of a cell pair immunostained for α-actinin (red), N-cadherin (white), and Nuclei (DAPI, blue). Graph below indicates the corresponding fluorescent intensity plotted across the cell pair with distance on the X-axis. (C) To calculate cell junction protein localization center and distribution, the normalized intensity profile for each cell pair was averaged across each cell pair. A Gaussian curve was fitted on the intensity profile to calculate the peak center (μ) and width (σ), defined as the Gaussian standard deviation. (D-E) Representative image of a hiPSC-CM cell pair immunostained for F-actin (green), α-actinin (red), and nuclei (DAPI, blue). Computed

skeletonized F-actin or α-actinin images are shown below, pseudo-colored based on angular fibril orientation. (F) Cartoon depicting cytoskeleton organization metrics. (G) Representative images illustrate relative coverages of two different WT cell pair patches. (H) Cytoskeletal organization metrics F-actin OOP, sarcomere OOP, and Z-disc presence calculated per batch for day 6 cell pairs. Cell pair cytoskeletal organization was consistent regardless of number of cells attached onto a substrate across multiple batches. Mean ± SD with statistically significant P-values (*P<0.05) indicated by one-way ANOVA followed by Tukey's HSD test.

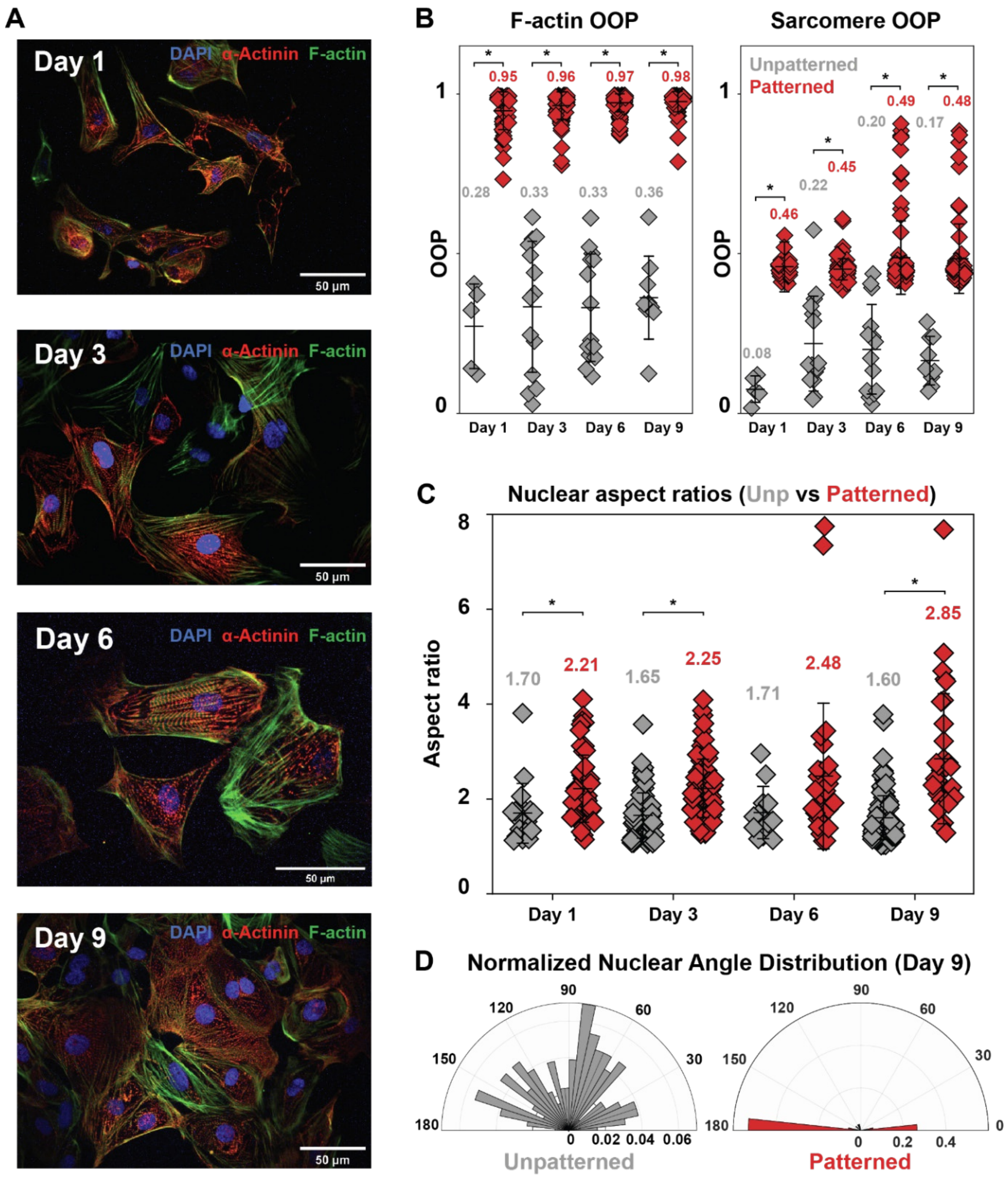

Figure S3. Pleomorphic structure of unpatterned hiPSC-CMs. Related to Fig. 1. (A) Representative unpatterned hiPSC-CMs were immunostained for F-actin (green), α-actinin (red), and nuclei (DAPI, blue) at day 1, 3, 6, or 9 on PDMS substrates. Scale bar, 50 μm. (B) F-actin and sarcomeric α-actinin OOP values of WT hiPSC-CMs without shape constraint (unpatterned, grey) or with constraint into cell pairs (patterned, red; WT data from figure 3). Patterned cell pairs had significantly greater organization at all time points examined. (C) Nuclear aspect ratios of pleomorphic unpatterned hiPSC-CMs and shape-controlled hiPSC-CM pairs over 9 days. (D) Nuclei of patterned cell pairs were aligned with the horizontal axis (the cell long axis), whereas nuclei of unpatterned cells did not exhibit a predominant nuclear orientation. Nuclear angle distribution was measured at day 9. Plots show mean ± SD with statistically significant P-values (*P<0.05) indicated by two-way ANOVA followed by Tukey's multiple comparisons test. N = 5, 15, 14, 9 field of views for UNP and 54, 86, 103, 69 cell pairs for patterned cells on days 1, 3, 6, and 9.

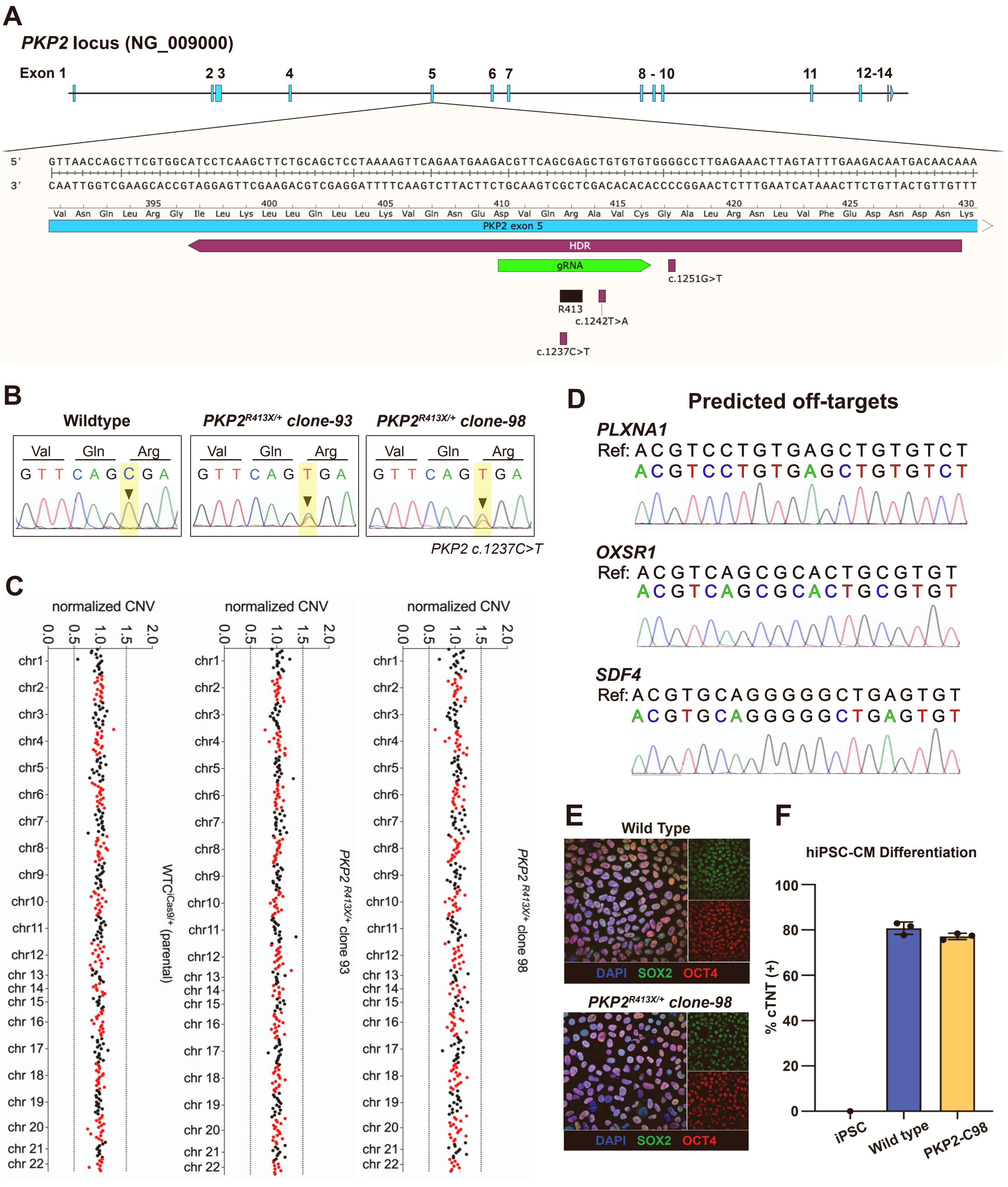

A
PKP2 locus (NG_009000)
Exon 1
2 3
4
5
6 7
8 - 10
11
12-14
PKP2 exon 5
HDR
gRNA
R413
c.1251G>T
c.1242T>A
c.1237C>T
B
Wildtype
PKP2R413X/+ clone-93
PKP2R413X/+ clone-98
Val Gln Arg
PKP2 c.1237C>T
C
normalized CNV
WTCiCas9/+ (parental)
PKP2 R413X/+ clone 93
PKP2 R413X/+ clone 98
D
Predicted off-targets
PLXNA1
Ref: ACGTCCTGTGAGCTGTGTCT
OXSR1
Ref: ACGTCAGCGCACTGCGTGT
SDF4
Ref: ACGTGCAGGGGGCTGAGTGT
E
Wild Type
DAPI SOX2 OCT4
PKP2R413X/+ clone-98
DAPI SOX2 OCT4
F
hiPSC-CM Differentiation
% cTNT (+)
iPSC
Wild type
PKP2-C98

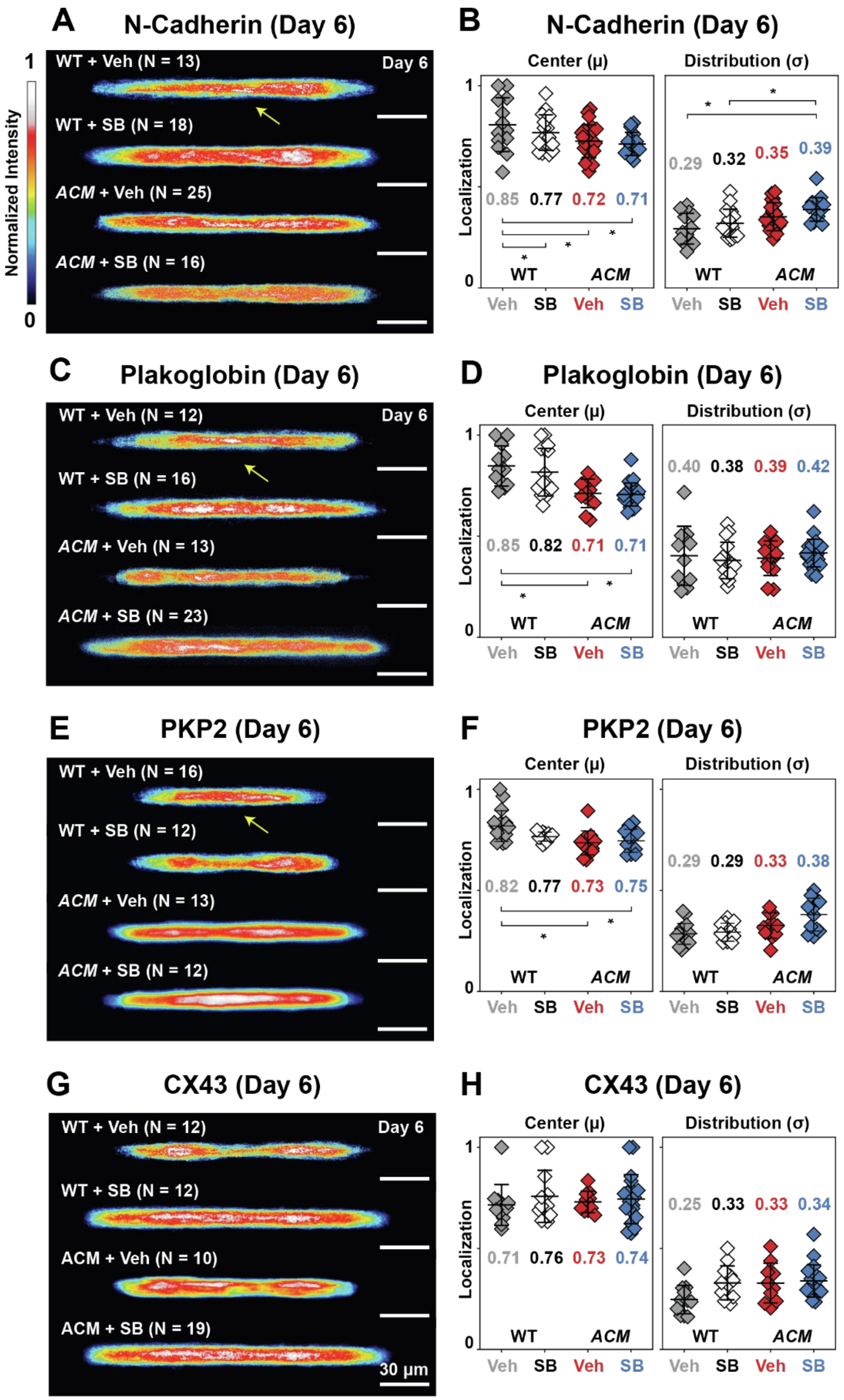

Figure S5. Effect of SB216763 treatment on WT and *ACM* hiPSC-CM pairs on day 6. Related to Fig. 5. WT or *ACM* hiPSC-CM pairs were treated with SB216763 or DMSO for 6 days. (A, C, E, G) Average localization heatmaps for the indicated junctional proteins on day 6. Yellow arrows indicate junctional localization and N, number of cell pairs analyzed over 3-5 independent differentiation batches. Quantitative analysis of N-cadherin (B), plakoglobin (E), and CX43 (F) localization at day 6 of culture with or without SB216763. Plots show mean ± SD. Statistically significant P-values (*P<0.05, by two-way ANOVA including Tukey's multiple comparisons test) are indicated.

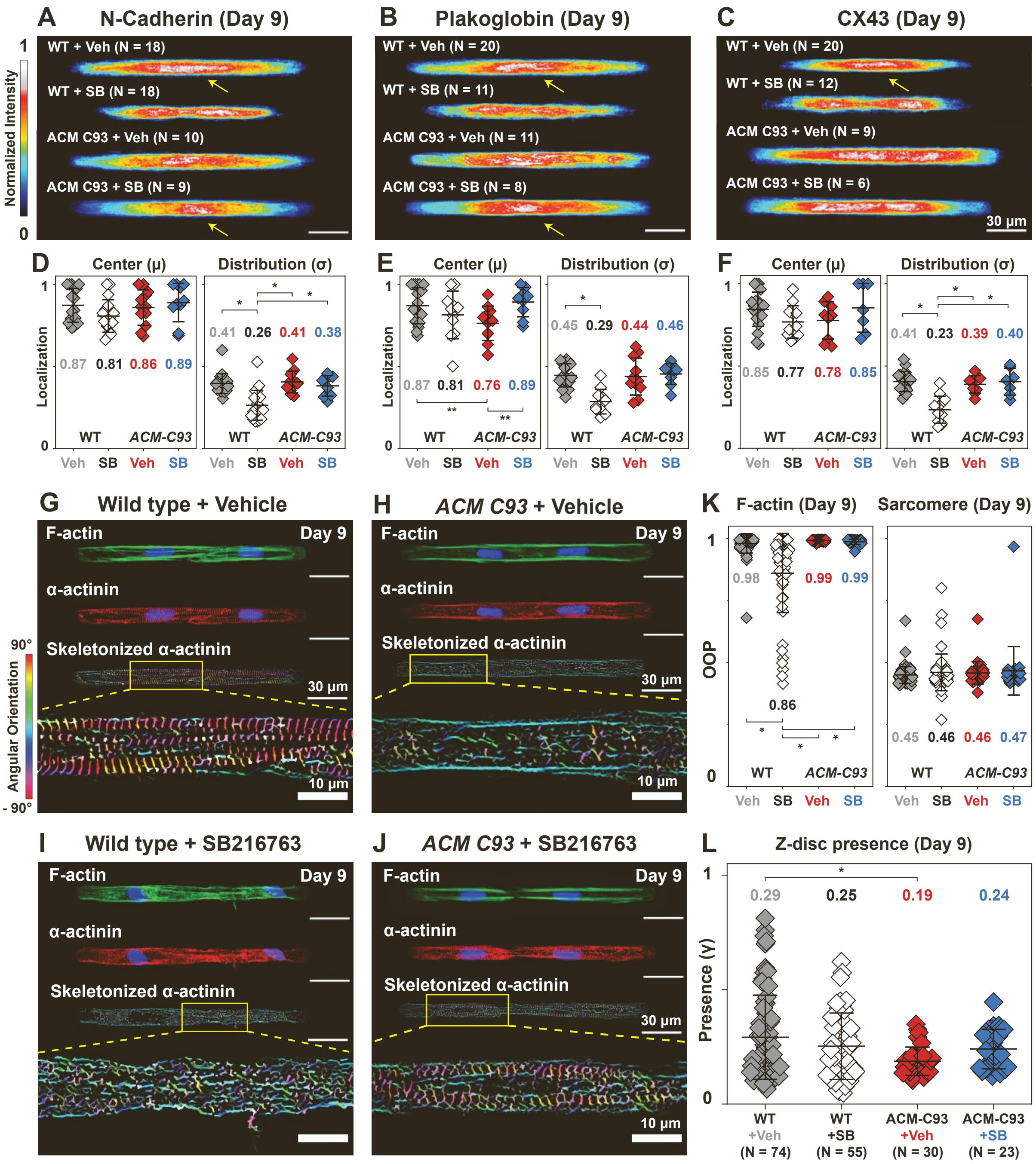

Figure S6. Analysis of hiPSC-CM pairs from an independent *PKP2*$^{R413X/+}$ ACM hiPSC line (clone 93). Related to Fig. 5. To validate observations made in the main *ACM* line (*PKP2*$^{R413X/+}$ clone 98), we studied a second line, clone 93. Measurements were made on day 9. WT data is from Figure 4 and 5. (A-C) Average localization heatmaps for the indicated junctional proteins. N, number of cell pairs analyzed. (D-F) Quantitative analysis of N-cadherin (D), Plakoglobin (E), and CX43 (F) localization. (G-J) Representative images of cell pairs stained for F-actin (green) and α-actinin (red). Images below show corresponding skeletonized fibrils, pseudo-colored based on angular orientation. (K-M) Quantified cytoskeletal structural metrics. Consistent with findings for *ACM clone 98*, SB216763 improved sarcomere OOP and Z-disc presence in *ACM clone 93*. Graphs show mean ± SD. Statistically significant P-values (*P<0.05 by two-way ANOVA including Tukey's multiple comparisons test) are indicated.

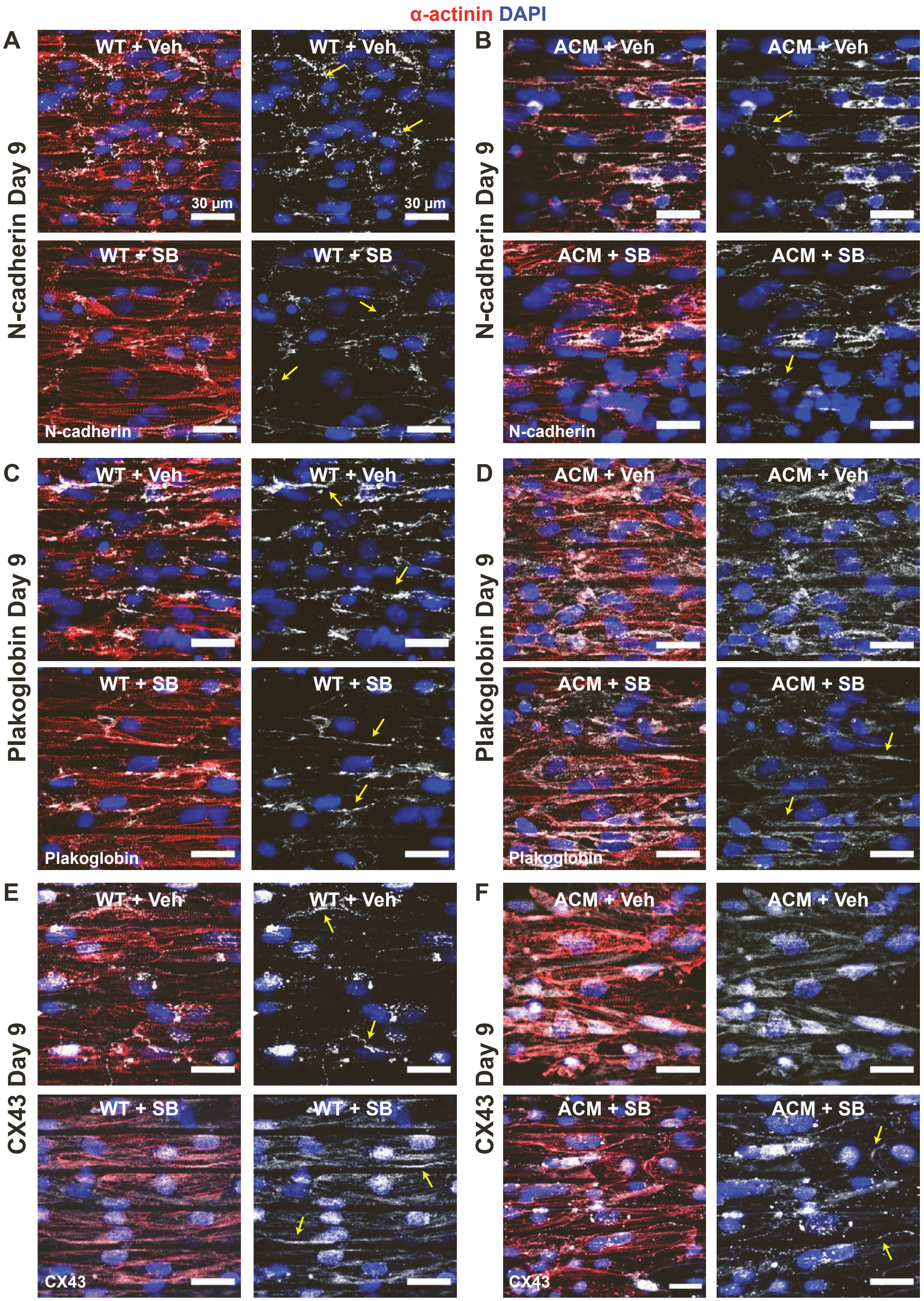

75 **Figure S7. hiPSC-CM tissues immunostained for junctional proteins. Related to Fig. 7.** (A-F) Representative images of day 9 hiPSC-CM tissues immunostained for sarcomeric α-actinin (red), nuclei (DAPI blue), N-cadherin (white) in WT (A) and *ACM* (B), plakoglobin (white) in WT (C) and *ACM* (D), CX43 (white) in WT (E) and *ACM* (F) with vehicle (upper panels) and SB216763 treatment (lower panels). Scale bar represents 30 µm, and yellow arrows indicate distinct plaques of protein expression on cell edges.

Target	Purpose	Direction	Sequence
PKP2 c.1237C>T	guide RNA	--	5’TTCTAATACGACTCACTATAGACGTTCAGCGA GCTGTGTGT GTTTTAGAGCTAGA-3’
PKP2 c.1237C>T	donor template	--	5’TGTTGTCATTGTCTTCAAATACTAAGTTTCTCA AGGCTCCACACACTGCTCACTGAACGTCTTCAT TCTGAACTTTTAGGAGCTGCAGAAGCTTGAGGAT-3’
PKP2^{R413}	genotyping	For	5’-GAAAAAGAGATGATGTAAGGCATCTGG-3’
		Rev	5’-GTTGTGCTACACATAGTTGTTGATGA-3’
OXSR1	off-target	For	5’-TTGGTCGCCATGCTTAGTGT-3’
		Rev	5’-CCTCGCCTCCTGCTCCTC-3’
PLXNA1	off-target	For	5’-CAGGGTCACCAGATCTGCC-3’
		Rev	5’-CTGGGCGGATGGGGAGAC-3’
SDF4	off-target	For	5’-AGCAGACAGGGAGGGGTG-3’
		Rev	5’-GGCACTGCCTCTGGGTGT-3’

Table S1. Nuclei acid sequences used for gene editing, genotyping, and analysis of off-targets. Related to Fig. 2. *PKP2*^{R413X} is caused by a single nucleotide polymorphism at *PKP2 c.1237C>T.* The underlined sequence in the guide RNA represents the target sequence for gene editing which is flanked by the T7 promoter (5’-) and the SpCas9 scaffold (-3’) sequence. The donor template is designed antisense to the *PKP2* locus and harbors three mutations: (1) the pathogenic *PKP2 c.1237C>T* mutation (red), (2) a silent mutation at *PKP2 c.1242T>A* (blue) to create a unique BstαI restriction enzyme site, and (3) a silent mutation at *PKP2 c.1251G>A* to ablate the guide RNA protospacer motif (green). *PKP2*^{R413X} primers amplify a 400bp fragment of *PKP2* exon 5 flanking the region targeted for gene editing. Primers for *OXSR1, PLXNA1,* and *SDF4* amplify 300-400bp fragments at predicted off target sites.

Supplemental Experimental Procedures

hiPSC-CM differentiation and culture

WTC-11 hiPSCs were obtained from the Coriell Institute (cat# GM252526) and genetically modified to express a doxycycline-inducible SpCas9 transgene in the AAVS1 safe harbor locus (called WTC-Cas9). In brief, the WTC-Cas9 line was created by transfecting pAAVS1-PDi-CRISPRn (Addgene #73500) and pSpCas9(BB)-2A-GFP (PX458) (Addgene #48138) in WTC-11 hiPSC and selecting for GFP positive clones with successful transgene integration. hiPSCs were maintained in Essential 8 (E8) medium (A1517001, Thermo Fisher). Isogenic hiPSCs harboring the pathogenic $PKP2^{R413X/+}$ (*PKP2 c.1237C>T*) variant were engineered using the CRISPR-Cas9 genome editing system as described previously [1]. In brief, a guide RNA (gRNA) was designed to target exon 5 of the human *PKP2* locus (Table S1) and *in vitro* transcribed using the EnGen sgRNA synthesis Kit (NEB, E3322S). A 100-nt donor template was then designed (Table S1) anti-sense to the gRNA and containing three sequence alterations: (1) the pathogenic *PKP2 c.1237C>T* variant, (2) a silent change at *PKP2 c.1242T>A* to create a unique BstαI restriction site for positive clone screening and (3) a silent variant at *PKP2 c.1251G>T* to prevent cleavage by the gRNA. At 50% confluency, Cas9 expression was induced in WTC-Cas9 hiPSCs by supplementing culture media with 2 μg/ml doxycycline (Sigma, D3072) for 18 hours. WTC-Cas9 hiPSCs were then electroporated with 5 μg purified gRNA and 5 μg donor template using an Amaxa Nucleofector system (Lonza). At 48 hours post-transfection, cells were sparsely seeded on a 10 cm plate for colony expansion and selection. Positive clones were verified using Sanger and amplicon sequencing. Potential gRNA off-targets were predicted using CHOPCHOP (https://chopchop.cbu.uib.no) [2] and three of the top predicted exon-coding off-targets were assessed by Sanger sequencing (Table S1). hiPSC pluripotency was verified by expression of OCT4 and SOX2. Karyotypes were assessed by copy number variation using the nCounter Human Karyotype Panel (NanoString Technologies).

To induce cardiomyocyte differentiation, we used WNT modulation [3] (Figure S2 A). hiPSCs were grown to 40-50% confluency. To induce differentiation, on differentiation day 0 (dd0) hiPSCs were cultured in RPMI 1640 medium (61870127, Thermo Fisher) supplemented with B27 minus Insulin (A1895601, Thermo Fisher) and 6 µM CHIR99021 (72054, StemCell Technologies). Cells were switched to RPMI/B27 and then RPMI/B27 supplemented with 5 µM IWR-1 endo (72564, StemCell Technologies) on dd2 and dd3, respectively. On dd5, cells were switched to RPMI/B27. On dd20, hiPSC-CMs underwent negative selection using 5 mM sodium DL-lactate (L7900, Sigma-Aldrich) for 2 – 4 days. After 30 days of differentiation, cells were dissociated by incubating with Accutase (7920, StemCell Technologies) for 30 minutes at 37°C. Cells were resuspended and pooled in equal volumes of Cardiomyocyte Support Medium (5027, StemCell Technologies), centrifuged, and resuspended for counting. Cells were then seeded onto PDMS coated shape-controlled substrates. For experiments involving SB216763 treatment, hiPSC-CMs were seeded and cultured in RPMI/B27 supplemented with 2.5 μM SB216763 or vehicle (DMSO) daily for 6 or 9 days.

PDMS substrate fabrication and cell pair microcontact printing

Microcontact printing for inducing cell pair formation has been reported previously [4–6]. Here we modified and optimized the protocols to tailor them for hiPSC-CMs (Figure S1). Photolithography masks with the desired pattern (array of 14:1 rectangles 211 μm x 15 μm for cell pairs and an array of 25 μm x 4 μm lines for engineered tissues) were drawn up in computer-aided design (CAD) format and converted to photomasks (Outputcity). A thin layer of SU-8 3005 photoresist was spin coated onto silicon wafers (Wafer World) and selectively polymerized by UV-light through the photomasks. The exposed wafers were developed using propylene glycol methyl ether acetate (PGMEA, Sigma). Polydimethylsiloxane (PDMS, Sylgard 184; Dow Corning) was cast and cured on the silicon wafers to create PDMS stamps for microcontact printing. PDMS coated coverslips were prepared by spin coating a 1:1 mix of 184 PDMS (10:1 base to curing agent) and 527 PDMS (1:1 part A and part B) onto glass coverslips. PDMS coated coverslips were treated for 8 mins in a UV Ozone cleaner (Jelight) immediately before microcontact printing with PDMS stamps incubated for an hour with fibronectin (FN) (50 μg/mL, BD Biosciences), LDEV-free GelTrex (GT) (1:200 dilution from stock, A1413302, Thermo Fisher), or a 1:1 mixture of these solutions. PDMS stamps upon contact were then removed, and the coverslips were incubated with room temperature 1% (w/v) Pluronic F-127 (BASF) for 10 minutes to prevent cell adhesion on unpatterned regions (Figure S1 D). After washing 3 times with phosphate-buffered saline (PBS, Invitrogen), coverslips were ready for cell plating. Fully dissociated hiPSC-CMs were then seeded at a density of 1E5 cells/ml in a 12 well plate.

Engineered tissue fabrication

Micro-molded gelatin tissues were fabricated by modification of previously published protocols for fabrication of gelatin muscular thin films [7]. A double layer of laboratory tape (General-Purpose Laboratory Labeling Tape, VWR) was affixed to an 18 mm square glass coverslip (Corning) or 1 mm thick Clear Scratch- and UV-Resistant Acrylic Sheet (McMaster-Carr) and an array of three tissue outlines (Figure 5 A) were laser cut with an Epilog Mini laser. The tissue chips were removed with tweezers, submerged in bleach for 10 minutes and washed with 70% ethanol for 30 min to remove any remaining residue. The chips were then washed with PBS three times and dried. A solution of 20% w/v gelatin (Sigma Aldrich) and 8% w/v microbial transglutaminase (MTG, Ajinomoto) were prepared with PBS and dissolved for 30 min at 65°C or 37°C, respectively. 5 ml of each solution (final concentration 10% w/v or 4% w/v total, respectively) were mixed and a 300 µl aliquot of the mixture was pipetted onto each tissue chip. PDMS stamps containing lines of 25 mm wide ridges, 4 mm grooves, and 5 mm groove depth were placed on top of the gelatin mixture aliquot and lightly pressed down with a 200 g scale calibration weight. These tissue chips were stored overnight at room temperature to cure, inside a glass container to prevent full dehydration. Then the calibration weights were removed, and the tissue chips were immersed in PBS for 30 min for rehydration and easy removal of PDMS stamps. These tissue chips were then submerged in 70% ethanol for 10 minutes under the UV light. The chips were then rinsed 3 times with PBS again and coated with a 1:100 dilution of GelTrex at 37°C for 1 hr or 4°C overnight. Fully dissociated hiPSC-CMs were then seeded at a density of 1E6 cells/ml for a total of 2E6 cells per well in a 12 well plate.

Ca^{2+} optical mapping and propagation velocity calculation

To prepare tissue substrates for Ca^{2+} optical mapping, day 6 and day 9 samples were incubated with 2 µM X-Rhod-1 AM (Invitrogen, X14210) in RPMI/B27 for 60 minutes at 37°C followed by dye-free media for an additional 15 minutes at 37°C. Relative cytoplasmic Ca^{2+} was imaged using a modified tandem-lens macroscope as described previously [8]. Videos were acquired at 400 frames per second using a high-speed camera (MiCAM Ultima, Scimedia) through a plan APO 0.63x or 1x objective, a collimator (Lumencor) and a 200-mW mercury lamp for epifluoresence illumination (X-Cite exacte, Lumen Dynamics). A filter set (excitation filter: 580/14 nm, dichroic mirror: 593 nm cut-off, emission filter 641/75, Semrock) was used for imaging. Propagation velocities were calculated based on post-processed data using MATLAB (MathWorks) and the MiCAM imaging software (Scimedia) [7]. A spatial filter of 3 x 3 pixels was applied to improve the signal-to-noise ratio, and the activation time of each pixel was calculated by the derivative of the fluorescent intensity (the average maximum upstroke slope) over a 10 second recording window. The propagating velocity was quantified in the narrow 3 to 5 mm region of the tissue while electrically pacing the tissues at 1 Hz with an IonOptix myopacer (10V amplitude, 10 ms pulse width) using two platinum electrodes (Sigma-Aldrich) with 1 mm spacing.

Immunostaining of hiPSC-CM cell pairs and tissues

Substrates were washed with PBS several times before fixation with 4% (v/v) paraformaldehyde with 0.05% (v/v) Triton X-100 in PBS for 10 minutes. To block non-specific binding, samples were incubated with 5% (w/v) bovine serum albumin (BSA) in PBS for 30 minutes. Samples were then incubated with primary antibodies against fibronectin (1:500; ab2413 abcam), sarcomeric α-actinin (1:500; ab9465 abcam), N-cadherin (1:500; ab76057 abcam), γ-catenin (1:500; MAB2083 Millipore), PKP2 (1:500; MABT394 Millipore), or CX43 (1:500; C6219 Sigma), in 1% (w/v) BSA in PBS overnight at 4°C. Alexa Fluor 488, 546, or 633 secondary antibodies (1:200 Invitrogen), DAPI (1:200 Invitrogen), and Alexa Fluor 488 or 633 conjugated Phalloidin (1:200 Invitrogen) were further incubated at room temperature for an hour and washed with PBS before mounting onto microscope slides with Prolong Gold anti-fade agent (Invitrogen). Slides were dried overnight and stored at 4°C until imaging on a spinning disk confocal microscope (Olympus IX83, Andor spinning disk).

Image extraction and Orientational Order (OOP) analysis

Image analysis to quantify OOP and cytoskeletal alignment were conducted with slight modifications to previously published methods [9–11]. Cytoskeletal actin and sarcomere OOP represent the overall angular alignment of processed fibrils, and Z-disc presence represents the fraction of cytoskeletal elements oriented in the orthogonal direction to the actin cytoskeleton (Figure S3 E). Preprocessing of images were performed with ImageJ/FIJI using the tubeness and OrientationJ plugins. Preprocessed images including the orientations of filamentous structures were then collated and the respective calculations conducted through MATLAB (Mathworks) [9].

Intercalated disc junctional localization metrics analysis

Image analysis for creating heatmaps and calculating protein localization were conducted through automated image processing scripts on ImageJ/FIJI and MATLAB [12]. Acquired cell pairs expressed sarcomeric α-actinin, a cardiomyocyte marker, covered most of the patterned area, and contained two distinct nuclei. Cell junction markers were not used to decide to include or exclude a cell pair. Suspected binucleated cells were excluded based on the criteria of nuclei being less than 50 μm apart, with horizontal cytoskeletal filaments spanning across both nuclei. Cell pairs were first automatically rotated and centered based on their fluorescent actin channel. For creating averaged heatmaps, auto-contrasting was applied across stacks of cell pairs and converted into a composite that depicted the average localization and distribution of a select protein (Figure S2 C). Sum Z-projections were then applied and pseudo-colored using a Royal lookup table where white represents completely saturated pixels. For calculating numerical quality metrics ranging from 0 to 1, individual fluorescent profiles of each channel were exported and analyzed separately. The 2D fluorescent intensity profile of the cell pair was split into a profile for each cell and averaged, with distance 0 representing the cell edge and 1 representing the cell junction (Figure S3 A and B). A Gaussian curve was then fitted on top of the averaged per cell intensity profile to extract values for the center of the curve and one standard deviation (the distribution/width). Calculated values for intensity center and distribution were tabulated and graphed.

Capillary westerns with subcellular fractionation for protein localization

Two million hiPSC-CMs were cultured in monolayers and treated with vehicle (DMSO) or 2.5 μM SB216763. On day 9, cells were harvested using Accutase and sub-cellular fractionation was performed using the Subcellular Protein Fractionation Kit (Thermo #78840). Protein samples (400 ng/lane) were analyzed by capillary western (ProteinSimple Wes device). Primary antibodies used were against N-cadherin (Invitrogen#33-3900, 1:100); plakoglobin (Millipore #MAB2083, 1:200); PKP2 (Millipore #MABT394, 1:300); CX43 (Abcam #ab11370, 1:400); GAPDH (Thermo# PA1-16777, 1:400); β-catenin (Abcam #ab32572, 1:400); Lamin B1 (Cell signaling #12586S, 1:1000); SERCA2 (Cell Signaling Technology #9580S, 1:400). Blots were normalized with respect to loading controls, GAPDH for cytosolic, LMNB1 for nuclear, and SERCA2 for membrane fractions. N-cadherin (CDH2) undergoes a series of post-translational modifications in its way to the cell membrane. Both 80 kDa and 100 kDa bands represent CDH2 still undergoing post-translational modification. Mean intensity values for both bands were combined before normalization to respective GAPDH bands.

Quantitative RT-PCR

hiPSC-CMs were seeded into 48-well plates at 100,000 cells per well and maintained for one week in RPMI/B27 medium. First-strand cDNA synthesis was then performed directly from cells using the FastLane Cell cDNA kit (215011, Qiagen) per manufacturer's instructions. Quantitative RT-PCR (qRT-PCR) was performed using Power SYBR Green PCR Master Mix (4368708, ThermoFisher) per manufacturer's instructions on a BioRad CFX384 qPCR Real-Time PCR machine. Relative levels of PKP2 transcripts were determined using the comparative C(T) method [13] and normalized to levels of GAPDH. Gene-specific primers used for qRT-PCR are found in Table S1.

Statistical analysis

Bar graphs indicate mean ± SD. Box plots represent the interquartile range and median (box and center line) and 1.5 times the interquartile range (whiskers). Statistical analysis across calculated values were conducted using one-way ANOVA or two-way ANOVA followed by Tukey's HSD test or test for multiple comparisons. Statistically significant P-values ($P<0.05$ and $P<0.1$) are indicated within the graphs where appropriate.

SUPPLEMENTAL REFERENCES

1. Wang G, Yang L, Grishin D, et al. Efficient, footprint-free human iPSC genome editing by consolidation of Cas9/CRISPR and piggyBac technologies. *Nat Protoc*. 2017;12(1):88-103. doi:10.1038/nprot.2016.152
2. Geiss GK, Bumgarner RE, Birditt B, et al. Direct multiplexed measurement of gene expression with color-coded probe pairs. *Nat Biotechnol*. 2008;26(3):317-325. doi:10.1038/nbt1385
3. Lian X, Zhang J, Azarin SM, et al. Directed cardiomyocyte differentiation from human pluripotent stem cells by modulating Wnt/β-catenin signaling under fully defined conditions. *Nat Protoc*. 2013;8(1):162-175. doi:10.1038/nprot.2012.150
4. McCain ML, Lee H, Aratyn-Schaus Y, Kléber AG, Parker KK. Cooperative coupling of cell-matrix and cell-cell adhesions in cardiac muscle. *Proc Natl Acad Sci U S A*. 2012;109(25):9881-9886. doi:10.1073/pnas.1203007109
5. Aratyn-Schaus Y, Pasqualini FS, Yuan H, et al. Coupling primary and stem cell-derived cardiomyocytes in an in vitro model of cardiac cell therapy. *J Cell Biol*. 2016;212(4):389-397. doi:10.1083/jcb.201508026
6. McCain ML, Desplantez T, Geisse NA, et al. Cell-to-cell coupling in engineered pairs of rat ventricular cardiomyocytes: relation between Cx43 immunofluorescence and intercellular electrical conductance. *Am J Physiol - Hear Circ Physiol*. 2012;302(2). doi:10.1152/ajpheart.01218.2010
7. Lee KY, Park SJ, Matthews DG, et al. An autonomously swimming biohybrid fish designed with human cardiac biophysics. *Science (80-)*. 2022;375(6581):639-647. doi:10.1126/science.abh0474
8. Park SJ, Zhang D, Qi Y, et al. Insights into the Pathogenesis of Catecholaminergic Polymorphic Ventricular Tachycardia from Engineered Human Heart Tissue. *Circulation*. 2019;140(5):390-404. doi:10.1161/CIRCULATIONAHA.119.039711
9. Pasqualini FS, Sheehy SP, Agarwal A, Aratyn-Schaus Y, Parker KK. Structural phenotyping of stem cell-derived cardiomyocytes. *Stem Cell Reports*. 2015;4(3):340-347. doi:10.1016/j.stemcr.2015.01.020
10. Sheehy SP, Pasqualini F, Grosberg A, Park SJ, Aratyn-Schaus Y, Parker KK. Quality metrics for stem cell-derived cardiac myocytes. *Stem Cell Reports*. 2014;2(3):282-294. doi:10.1016/j.stemcr.2014.01.015
11. Wang G, McCain ML, Yang L, et al. Modeling the mitochondrial cardiomyopathy of Barth syndrome with induced pluripotent stem cell and heart-on-chip technologies. *Nat Med*. 2014;20(6):616-623. doi:10.1038/nm.3545
12. Kim S. Cell pair heatmap overlay and intensity profile extraction/fitting. Published online September 28, 2022. doi:10.5281/ZENODO.7120682
13. Schmittgen TD, Livak KJ. Analyzing real-time PCR data by the comparative CT method. *Nat Protoc*. 2008;3(6):1101-1108. doi:10.1038/nprot.2008.73
